# Supplementary material for: Acceptability, feasibility and appropriateness of intensified health education, SMS/phone tracing and transport reimbursement for uptake of voluntary medical male circumcision in a sexually transmitted infections clinic in Malawi: A mixed methods study
Source: PLoS One. 2025 Jan 24;20(1):e0301952. doi: 10.1371/journal.pone.0301952 (PMC11760565; doi:10.1371/journal.pone.0301952)
Supplement: S1 Checklist — (DOC) [file pone.0301952.s001.doc]

**S1: Supplemental Material 1**

**Good Reporting of A Mixed Methods Study (GRAMMS) checklist**

**Acceptability, feasibility and appropriateness of the RITe intervention**

| **Guideline** | **Section: page** |
| --- | --- |
| Describe the justification for using a mixed methods approach to the | Methods- under |
| research question | Study design pg. 5-6 |
|  |  |
| Describe the design in terms of the purpose, priority and sequence of | Methods- study design |
| methods | pg. 5-6;  Data collection pg. 8-10 |
| Describe each method in terms of sampling, data collection and | Methods – Recruitment, sampling and sample size pg. 7-8;  Data collection pg. 8-10; |
| analysis | Analysis: pg.10-11 |
| Describe where integration has occurred, how it has occurred and | Methods- study design |
| who has participated in it | pg. 5-6;  Data collection pg. 8-10  Results: pg. 12-27 |
| Describe any limitation of one method associated with the present of | Discussion pg. 27-32 |
| the other method |  |
| Describe any insights gained from mixing or integrating methods | Discussion: pg. 27-32 |

O'Cathain A, Murphy E, Nicholl J. The quality of mixed methods studies in health services research. J Health Serv Res Policy. 2008;13: 92-98.
