# Supplementary material for: Acceptability, feasibility and appropriateness of intensified health education, SMS/phone tracing and transport reimbursement for uptake of voluntary medical male circumcision in a sexually transmitted infections clinic in Malawi: A mixed methods study
Source: PLoS One. 2025 Jan 24;20(1):e0301952. doi: 10.1371/journal.pone.0301952 (PMC11760565; doi:10.1371/journal.pone.0301952)
Supplement: S1 File — (DOCX) [file pone.0301952.s002.docx]

## **S2: Supplemental Material 2**

## **Survey: Acceptability, Appropriateness and Feasibility of the RITe at Bwaila STI Clinic**

**Note: All items in parenthesis refer to healthcare worker survey**

1. **Intensified Health Education**

**Baseline Acceptability (Men and Healthcare worker)**

|  | Completely disagree | Disagree | Neither agree nor disagree | Agree | Completely agree |
| --- | --- | --- | --- | --- | --- |
| 1. I would like to learn/educate men about VMMC | ➀ | ➁ | ➂ | ➃ | ➄ |
| 1. I approve learning/educating men about VMMC | ➀ | ➁ | ➂ | ➃ | ➄ |
| 1. Learning/educating men about VMMC at this clinic is a welcome idea to me | ➀ | ➁ | ➂ | ➃ | ➄ |
| 1. I find learning/educating men about VMMC appealing | ➀ | ➁ | ➂ | ➃ | ➄ |

**Baseline Appropriateness (men and healthcare workers)**

|  | Completely disagree | Disagree | Neither agree nor disagree | Agree | Completely agree |
| --- | --- | --- | --- | --- | --- |
| 1. Openly talking/educating men about VMMC is a good match to this clinic | ➀ | ➁ | ➂ | ➃ | ➄ |
| 1. Openly talking/educating men about VMMC is a good fit to me/for this clinic (probe-culture) | ➀ | ➁ | ➂ | ➃ | ➄ |
| 1. Openly talking/educating men about VMMC is suitable for me/this clinic (probe-religion) | ➀ | ➁ | ➂ | ➃ | ➄ |
| 1. Openly talking/educating men about VMMC is applicable to this clinic | ➀ | ➁ | ➂ | ➃ | ➄ |

**End-line Acceptability**

|  | Completely disagree | Disagree | Neither agree nor disagree | Agree | Completely agree |
| --- | --- | --- | --- | --- | --- |
| 1. I would liked to learn/educate men about VMMC | ➀ | ➁ | ➂ | ➃ | ➄ |
| 1. I approved learning/educating men about VMMC | ➀ | ➁ | ➂ | ➃ | ➄ |
| 1. Learning/educating men about VMMC at this clinic was a welcome idea to me | ➀ | ➁ | ➂ | ➃ | ➄ |
| 1. I found learning/educating men about VMMC appealing | ➀ | ➁ | ➂ | ➃ | ➄ |

**End-line Appropriateness**

|  | Completely disagree | Disagree | Neither agree nor disagree | Agree | Completely agree |
| --- | --- | --- | --- | --- | --- |
| 1. Openly talking/educating men about VMMC was a good match to this clinic | ➀ | ➁ | ➂ | ➃ | ➄ |
| 1. Openly talking/educating men about VMMC was a good fit to me/for this clinic (probe-culture) | ➀ | ➁ | ➂ | ➃ | ➄ |
| 1. Openly talking/educating men about VMMC was suitable for me/this clinic (probe-religion) | ➀ | ➁ | ➂ | ➃ | ➄ |
| 1. Openly talking/educating men about VMMC was applicable to this clinic | ➀ | ➁ | ➂ | ➃ | ➄ |

**Feasibility (healthcare workers only)**

|  | Completely disagree | Disagree | Neither agree nor disagree | Agree | Completely agree |
| --- | --- | --- | --- | --- | --- |
| 1. Intensified health education was implementable at this clinic | ➀ | ➁ | ➂ | ➃ | ➄ |
| 1. It was possible to routinely conduct intensified health education at this clinic. | ➀ | ➁ | ➂ | ➃ | ➄ |
| 1. Intensified health education was easy to conduct. | ➀ | ➁ | ➂ | ➃ | ➄ |
| 1. Intensified health education was doable at this clinic | ➀ | ➁ | ➂ | ➃ | ➄ |

1. **SMS/Telephone Tracing**

**Baseline Acceptability**

|  | Completely disagree | Disagree | Neither agree nor disagree | Agree | Completely agree |
| --- | --- | --- | --- | --- | --- |
| 1. I would like to receive/send SMS reminders/calls about VMMC appointments | ➀ | ➁ | ➂ | ➃ | ➄ |
| 1. Sending/receiving SMS reminders/calls about VMMC appointments is appealing to me | ➀ | ➁ | ➂ | ➃ | ➄ |
| 1. I approve SMS reminders/calls for VMMC appointments at this clinic (same for men and HCWs) | ➀ | ➁ | ➂ | ➃ | ➄ |
| 1. I welcome SMS reminders/calls for VMMC (same for men and HCWs) |  |  |  |  |  |

**Baseline Appropriateness**

|  | Completely disagree | Disagree | Neither agree nor disagree | Agree | Completely agree |
| --- | --- | --- | --- | --- | --- |
| 1. Receiving/sending SMS reminders/calls about VMMC appointments is a good match for this clinic | ➀ | ➁ | ➂ | ➃ | ➄ |
| 1. Receiving/sending SMS reminders/calls about VMMC appointments is a good fit to me/for this clinic (probe-culture) | ➀ | ➁ | ➂ | ➃ | ➄ |
| 1. Receiving/sending SMS reminders/calls about VMMC appointments is suitable to me/this clinic (probe-religion) | ➀ | ➁ | ➂ | ➃ | ➄ |
| 1. Receiving/sending) SMS reminders/calls about VMMC appointments is applicable to me/this clinic | ➀ | ➁ | ➂ | ➃ | ➄ |

**End-line Acceptability**

|  | Completely disagree | Disagree | Neither agree nor disagree | Agree | Completely agree |
| --- | --- | --- | --- | --- | --- |
| 1. I liked to receive/send SMS reminders/calls about VMMC appointments | ➀ | ➁ | ➂ | ➃ | ➄ |
| 1. Sending/receiving SMS reminders/calls about VMMC appointments was appealing to me | ➀ | ➁ | ➂ | ➃ | ➄ |
| 1. I approved SMS reminders/calls for VMMC appointments at this clinic (same for men and HCWs) | ➀ | ➁ | ➂ | ➃ | ➄ |
| 1. I welcomed SMS reminders/calls for VMMC (same for men and HCWs) | ➀ | ➁ | ➂ | ➃ | ➄ |

**End-line Appropriateness**

|  | Completely disagree | Disagree | Neither agree nor disagree | Agree | Completely agree |
| --- | --- | --- | --- | --- | --- |
| 1. Receiving/sending SMS reminders/calls about VMMC appointments was a good match for this clinic | ➀ | ➁ | ➂ | ➃ | ➄ |
| 1. Receiving/sending SMS reminders/calls about VMMC appointments was a good fit to me/for this clinic (probe-culture) | ➀ | ➁ | ➂ | ➃ | ➄ |
| 1. Receiving/sending SMS reminders/calls about VMMC appointments was suitable to me/this clinic (probe-religion) | ➀ | ➁ | ➂ | ➃ | ➄ |
| 1. Receiving/sending) SMS reminders/calls about VMMC appointments was applicable to me/this clinic | ➀ | ➁ | ➂ | ➃ | ➄ |

**Feasibility**

| 1. SMS/phone reminders were implementable at this clinic | ➀ | ➁ | ➂ | ➃ | ➄ |
| --- | --- | --- | --- | --- | --- |
| 1. It was possible to routinely conduct SMS/phone reminders at this clinic | ➀ | ➁ | ➂ | ➃ | ➄ |
| 1. SMS/phone reminders were easy to conduct | ➀ | ➁ | ➂ | ➃ | ➄ |
| 1. The clinic had the resources to support SMS/phone reminders | ➀ | ➁ | ➂ | ➃ | ➄ |

1. **Transport reimbursement**

**Baseline Acceptability**

| 1. I would like to receive transport reimbursement for my VMMC appointment. (HCW- I like the idea of providing transport reimbursement for men who will undergo VMMC). | ➀ | ➁ | ➂ | ➃ | ➄ |
| --- | --- | --- | --- | --- | --- |
| 1. Receiving transport reimbursement for my VMMC appointment is appealing. (HCW- Providing transport reimbursement for VMMC appointments is appealing) | ➀ | ➁ | ➂ | ➃ | ➄ |
| 1. I approve transport reimbursements for VMMC at this clinic. (both men and HCWs) | ➀ | ➁ | ➂ | ➃ | ➄ |
| 1. Transport reimbursements are a welcome idea to me. (both men and HCWs) | ➀ | ➁ | ➂ | ➃ | ➄ |

**Baseline Appropriateness**

| 1. Receiving/providing transport reimbursement for VMMC appointment is a good match for me/this clinic | ➀ | ➁ | ➂ | ➃ | ➄ |
| --- | --- | --- | --- | --- | --- |
| 1. Receiving/providing transport reimbursement for VMMC appointment is a good fit to me/this clinic (probe-culture) | ➀ | ➁ | ➂ | ➃ | ➄ |
| 1. Receiving/providing transport reimbursement for VMMC appointment is suitable to me/this clinic (probe-religion) | ➀ | ➁ | ➂ | ➃ | ➄ |
| 1. Receiving/providing transport reimbursement for VMMC appointment is applicable to me/this clinic | ➀ | ➁ | ➂ | ➃ | ➄ |

**End-line Acceptability**

| 1. I liked receiving transport reimbursement for my VMMC appointment. (HCW- I liked the idea of providing transport reimbursement for men who underwent VMMC). | ➀ | ➁ | ➂ | ➃ | ➄ |
| --- | --- | --- | --- | --- | --- |
| 1. Receiving transport reimbursement for my VMMC appointment was appealing. (HCW- Providing transport reimbursement for VMMC appointments was appealing) | ➀ | ➁ | ➂ | ➃ | ➄ |
| 1. I approved transport reimbursements for VMMC at this clinic. (both men and HCWs) | ➀ | ➁ | ➂ | ➃ | ➄ |
| 1. Transport reimbursements were a welcome idea to me. (both men and HCWs) | ➀ | ➁ | ➂ | ➃ | ➄ |

**End-line Appropriateness**

| 1. Receiving/providing transport reimbursement for VMMC appointment was a good match for me/this clinic | ➀ | ➁ | ➂ | ➃ | ➄ |
| --- | --- | --- | --- | --- | --- |
| 1. Receiving/providing transport reimbursement for VMMC appointment was a good fit to me/this clinic (probe-culture) | ➀ | ➁ | ➂ | ➃ | ➄ |
| 1. Receiving/providing transport reimbursement for VMMC appointment was suitable to me/this clinic (probe-religion) | ➀ | ➁ | ➂ | ➃ | ➄ |
| 1. Receiving/providing transport reimbursement for VMMC appointment was applicable to me/this clinic | ➀ | ➁ | ➂ | ➃ | ➄ |

**Feasibility**

| 1. Transport reimbursements were implementable at this clinic | ➀ | ➁ | ➂ | ➃ | ➄ |
| --- | --- | --- | --- | --- | --- |
| 1. It was possible to routinely provide transport reimbursements at this clinic. | ➀ | ➁ | ➂ | ➃ | ➄ |
| 1. Proving transport reimbursements was easy. | ➀ | ➁ | ➂ | ➃ | ➄ |
| 1. Proving transport reimbursements was doable at this clinic. | ➀ | ➁ | ➂ | ➃ | ➄ |
