# Supplementary material for: Acceptability, feasibility and appropriateness of intensified health education, SMS/phone tracing and transport reimbursement for uptake of voluntary medical male circumcision in a sexually transmitted infections clinic in Malawi: A mixed methods study
Source: PLoS One. 2025 Jan 24;20(1):e0301952. doi: 10.1371/journal.pone.0301952 (PMC11760565; doi:10.1371/journal.pone.0301952)
Supplement: S2 File — (DOC) [file pone.0301952.s003.doc]

## **S3: Supplemental Material 3**

## **Interview Guide: Acceptability, Appropriateness and Feasibility of the RITe Intervention at Bwaila STI clinic**

1. **Baseline in-depth interview guide for healthcare workers**
2. ***Demographics***

**Interviewer initials**: ________________ **Date of interview** _________________

**Respondent ID**: ____________________ **Age (years**) ___________________

**Sex** **** Male _(1)_ **** Female _(2)_

**Position**  Nurse _(0)_  HTS counsellor _(1)_  Clinician _(2)_  Other _(3)_

**Duration at current position**  <5 years _(0)_  5 – 10 years _(1)_  >10 years

1. ***Introduction***

*Interviewer: My name is __________ and I am working with UNC Project. Thank you for your willingness to speak with me today. I would like to ask you a series of questions to find out how you feel about an intervention being tried out at this clinic. The intervention is called RITe which stands for* ***R****eimbursement for transport,* ***I****ntensified health education and SMS* ***T****racing. We are trying this intervention to see if it can improve the men’s desire for medical circumcision and increase the number of men going for circumcision. All your responses are confidential. Your name is not on this form. If you cannot, or do not wish to answer a particular question, tell me and I will go on to the next one. Please answer questions honestly. Remember, there are no right or wrong answers to these questions, just answer them as best as you can. The questionnaire will be the same length of time regardless of the answers you give. Do you have any questions?*

*Personal Questions.*

1. Tell me more about your role at this clinic?

Probe: Can you briefly explain you daily activities

1. **Acceptability and Appropriateness**
2. How open are you to talk about circumcision?
   - 1. Please explain why?
3. How open do you think people (both males and females) at this clinic would be to talk about circumcision?
   - 1. Please explain why?
     2. How do you think men in particular will react to a discussion about circumcision, why?

*We are proposing to conduct intensified health education on circumcision at this clinic. Intensified health education will regular group health education talks on circumcision. The education will focus on what circumcision is, its proven benefits and common misconceptions about circumcision. We will also allow patients to ask questions about circumcision. We propose to also involve men who have successfully undergone circumcision and their spouses (women) to share experiences around circumcision.*

1. What are your thoughts about using intensified health education as a strategy for scale-up of VMMC at this clinic? Probe

*We also intend to send SMS reminders to men who have a circumcision appointment. The SMS text will be carefully worded or coded for confidentiality. The messages will be sent two days, a day before and on the day of the circumcision appointment.*

1. What are your thoughts about using SMS tracing as a strategy for scale-up of VMMC at this clinic? Probe

*We are also proposing to provide transport reimbursement to men who will undergo circumcision to help with expense incurred on the day of circumcision. The reimbursement will be an equivalent of $10 in Malawian Kwacha based on the National Health Sciences Research Ethics Committee guidelines. The reimbursement will be from a designated nurse within the STI clinic.*

1. What are your thoughts about using transport reimbursement as a strategy for scale-up of VMMC at this clinic? Probe

*Lastly, we intend to try implementing all the interventions discussed above together to see how they will impact of the number of men who will choose to get medical circumcision.*

1. What are your thoughts about these intervention components used in combination? Probe – would they work, would it be too much, which combination, if any?
2. How do you think these intervention components align with the activities of this clinic? Probe.
3. How do you think these intervention components align with culture or religious beliefs in Malawi? Probe.
4. Is there anything else you would like to share with me?
5. Do you have any questions or comments?

Thank you for your time and contribution.

## **Baseline focus group discussion guide for men**

- - 1. **Demographics**

| **Respondent ID** | **Respondent initials** | **Age** | **Marital status**  Single _(0)_  Married _(1)_ Divorced _(2)_ Widowed_(3)_ | **Area of residence** | **Occupation** | **Education level**  None _(0)_ Primary_(1)_  Secondary _(2)_ >Secondary _(3)_ | **Running water**  Yes _(1)_  No _(2)_ | **Electricity**  Yes _(1)_  No _(2)_ |
| --- | --- | --- | --- | --- | --- | --- | --- | --- |
|  |  |  |  |  |  |  |  |  |
|  |  |  |  |  |  |  |  |  |

- - 1. **Introduction**

*Modulator: My name is __________and I am working with the University of North Carolina Project. Thank you for your willingness to speak with me today. I would like to discuss about an intervention being tried out at this clinic. The intervention is called RITe which stands for* ***R****eimbursement for transport,* ***I****ntensified health education and SMS* ***T****racing. We are trying this intervention to see if it can improve the desire for men to undergo medical circumcision and increase the number of men going for circumcision. All your responses are confidential. Your names will not appear on any forms. Please feel free to discuss your honest opinions. Remember, there is no right or wrong answer, just discuss openly. Do you have any questions?*

*To begin, we want to discuss medical circumcision itself.*

1. What have you heard about medical circumcision?
2. Can you explain to me about the benefits of medical circumcision?
3. Can you explain to me what you heard about downsides of medical circumcision?
4. How would you feel about undergoing medical circumcision? Probe about pain and discomfort, wound care, disclosure to friends, relatives and partner
   - 1. **Acceptability and Appropriateness**

*Thanks for sharing. Now we want to focus the discussion on what we are planning for the clinic to get your reactions and ideas.*

*Firstly, we would like to conduct regular and more detailed education on circumcision.*

1. How do you feel about receiving education on medical circumcision at this clinic? Probe – What type of information on circumcision would you like to receive? What information about circumcision would be inappropriate? How do you feel about receiving information about circumcision from previously circumcised men? How do you feel about receiving information about the benefits of circumcision from a female partner of a circumcised man?

*Now I would like you to discuss how you would respond to a number of activities if you decided to undergo VMMC.*

1. How would you feel about receiving a SMS or a phone call from a healthcare worker at this clinic to remind you about an appointment at VMMC clinic? Probe. Is this fine with your culture? Is this fine with your religious beliefs?
2. How would you feel about receiving money after being circumcised to help with your transportation costs? Probe. Would you rather receive something else instead of money?
3. What are your thoughts about using education, SMS reminders and receiving money in combination? Probe. What combinations do you like the most?
4. Is there anything else you would like to share with me?
5. Do you have any questions or comments?

Thank you for your time and contribution.

1. **End-line in-depth interview guide for healthcare workers**
2. ***Demographics***

**Interviewer initials**: ________________ **Date of interview** _________________

**Respondent ID**: ____________________ **Age (years**) ___________________

**Sex** **** Male _(1)_ **** Female _(2)_

**Position**  Nurse _(0)_  HTS counsellor _(1)_  Clinician _(2)_  Other _(3)_

**Duration at current position**  <5 years _(0)_  5 – 10 years _(1)_  >10 years

1. ***Introduction***

*Interviewer: My name is __________and I am working with UNC Project. Thank you for your willingness to speak with me today. I would like to ask you a series of questions to find out about your experiences with the intervention we tried out at this clinic. The intervention was called RITE which stands for* ***R****eimbursement for transport,* ***I****ntensified health education and SMS* ***T****racing. We tried this intervention to see if it could improve the men’s desire for medical circumcision and increase the number of men going for circumcision. All your responses are confidential. Your name is not on this form. If you cannot, or do not wish to answer a particular question, tell me and I will go on to the next one. Please answer questions honestly. Remember, there are no right or wrong answers to these questions, just answer them as best as you can. The questionnaire will be the same length of time regardless of the answers you give. Do you have any questions?*

*Please note that this is a follow-up interview to the initial interviews conducted at the beginning on this study. Since you participated in the initial interviews, this interview aims to seek your responses on how you felt our study intervention performed.*

1. **Acceptability and Appropriateness**

*Now we want to focus the discussion on the intervention we implemented.*

*Intensified Health Education*

1. What were your thoughts about using intensified health education as a strategy for scale-up of VMMC at this clinic? Probe

*SMS and Telephone tracing*

1. What were your thoughts about using SMS tracing as a strategy for scale-up of VMMC at this clinic? Probe

*Transport Reimbursement*

1. What were your thoughts about using transport reimbursement as a strategy for scale-up of VMMC at this clinic? Probe

*Full intervention*

1. What were your thoughts about these intervention strategies used in combination? Probe – did they work, was it too much, which combination worked best, if any?
2. How did you think these intervention strategies aligned with the activities of this clinic? Probe.
3. How did you think these intervention strategies aligned with culture or religious beliefs in Malawi? Probe.

**Feasibility**

1. What were your thoughts about how education, SMS reminders and provision of transport reimbursement were implemented at this clinic? Probe. What could have been done differently?
2. What were your thoughts about making education, SMS reminders and provision of transport reimbursement part of routine activities at this clinic? Probe. What combinations worked well as routine practice? What combinations worked well with the available resources?
3. Is there anything else you would like to share with me concerning this intervention?
4. Do you have any questions or comments?

Thank you for your time and contribution.

1. **Follow-up focus group discussion for men**
2. **Demographics**

| **Respondent ID** | **Respondent initials** | **Age** | **Marital status**  Single _(0)_  Married _(1)_ Divorced _(2)_ Widowed_(3)_ | **Area of residence** | **Occupation** | **Education level**  None _(0)_ Primary_(1)_  Secondary _(2)_ >Secondary _(3)_ | **Running water**  Yes _(1)_  No _(2)_ | **Electricity**  Yes _(1)_  No _(2)_ |
| --- | --- | --- | --- | --- | --- | --- | --- | --- |
|  |  |  |  |  |  |  |  |  |
|  |  |  |  |  |  |  |  |  |

1. **Introduction**

*Modulator: My name is __________ and I am working with UNC Project. Thank you for your willingness to speak with me today. You have been invited today because you participated in the baseline interview on an intervention we tried out at this clinic. The intervention is called RITE which stands for* ***R****eimbursement for transport,* ***I****ntensified health education and SMS* ***T****racing. We tried this intervention to see if it improved the desire for men to undergo medical circumcision and increase the number of men going for circumcision. All your responses are confidential. Your names will not appear on any forms. Please feel free to discuss your honest opinions. Remember, there is no right or wrong answers, just discuss openly. Do you have any questions?*

1. **Acceptability and Appropriateness**

*Now we want to focus the discussion on the intervention we implemented.*

*Intensified Health Education*

1. How did you feel about receiving education on medical circumcision at this clinic?
2. What type of information on circumcision did you like to receive?
3. What information about circumcision did you find inappropriate?
4. How did you feel about receiving information about circumcision from previously circumcised men?
5. How did you feel about receiving information about circumcision from a female partner of a circumcised man?

*SMS and Telephone Tracing*

1. How did you feel about receiving a SMS or a phone call from a healthcare worker at this clinic to remind you about an appointment at VMMC clinic? Probe. Was this fine with your culture? Was this fine with your religious beliefs?

*Transport reimbursement*

1. How did you feel about receiving money after being circumcised to help with your transportation costs? Probe. Did you prefer to receive something else instead of money?

*Full intervention*

1. What were your thoughts about using education, SMS reminders and receiving money in combination? Probe. What combinations do you like the most?
2. **Feasibility**
3. What are your thoughts about how education, SMS reminders and provision of money were implemented at this clinic? Probe. What could have been done differently?
4. What are your thoughts about making education, SMS reminders and provision of money part of routine activities at this clinic? Probe. What combinations do you want to be routine practice?
5. Is there anything else you would like to share with me about the implementation of the intervention?
6. Do you have any questions or comments?

Thank you for your time and contribution.
