# Supplementary material for: Acceptability, feasibility and appropriateness of intensified health education, SMS/phone tracing and transport reimbursement for uptake of voluntary medical male circumcision in a sexually transmitted infections clinic in Malawi: A mixed methods study
Source: PLoS One. 2025 Jan 24;20(1):e0301952. doi: 10.1371/journal.pone.0301952 (PMC11760565; doi:10.1371/journal.pone.0301952)
Supplement: S1 Data — (ZIP) [file pone.0301952.s004.zip › Qualitative data/Baseline FGD Transcripts/FGD1_Transcript.docx]

1. I: Okay, to start, tell me what you have heard about medical circumcision.
2. R5: Okay, we have heard that you want to know the number of people undergoing medical circumcision versus the number of people having it done at home.
3. I: Okay, even previously, what have you heard about what circumcision is, or it’s benefits or anything.
4. R1: I heard that male circumcision is a way of reducing the risk of contracting sexually transmitted diseases, yes. Many people think that someone who has undergone circumcision is part of the youth, but that is not the case.
5. I: What do you mean when you say ‘part of the youth’?
6. R1: Umm, most girls in the communities think that a man who is not circumcised is not part of the men they can chat with; they cannot be in a relationship with a man who is not circumcised. But the main issue is that when you are circumcised and you have unprotected sex, you would not contract diseases.
7. I: Okay, he heard that it protects you from contracting diseases, what else have we heard?
8. R5: People say that once you are circumcised, you will not contract any disease. Even if you slept with someone who has HIV and is very sick, you would not contract it. You would not even contract things like gonorrhea or syphilis, you would be free of it all. If you are not circumcised, you can contract any disease and in short, you would die quickly.
9. I: Okay, I saw you also raised a hand.
10. R6: Yes, the question was on what we have heard about circumcision right?
11. I: Yes.
12. R6: What I heard was that circumcision is the removal of the foreskin…
13. R5: Removing the hat
14. R6: Yes, removing the skin for different reasons. From what I heard, when you are not circumcised, the tip of your penis is soft. When it is soft, if you are accidentally bruised while having sex and the one you are sleeping with also gets bruised and you both start to bleed, if either of you have any sexually transmitted diseases, then you will transmit it to each other.
15. I: Okay.
16. R6: [Hesitant] What I also heard…
17. I: Yes, I would like to hear everything you have heard before.
18. R6: Yes, some say that having sex with someone who is circumcised is more enjoyable unlike having sex with one who is not circumcised.
19. I: Okay.
20. R3: Just to add on the same point, what I heard about circumcision is that it helps prevent sexually transmitted diseases. When you remove the foreskin during circumcision, you make the skin that is left inside thick such that it would not bruise during sexual intercourse.
21. I: Okay, most of us are speaking of sexually transmitted diseases… oh sorry, please go ahead.
22. R4: What I heard on circumcision is that it is also a way of maintaining hygiene. When you are circumcised, you are clean and you would not contract diseases anyhow.
23. I: Okay, is there anything else we would like to add?
24. R1: Yes, I also just wanted to say that most people in the communities have established that circumcision is for Muslims, but that is not the case. Once the skin is removed, the things that are found in it would not be there and those things cause diseases. So, it is important for the young people to remove the mentality that circumcision is only for the Muslims in order to protect ourselves and the people we have sex with.
25. I: Okay, why do you think people say that circumcision is only for the Muslims? Anyone can answer by the way.
26. R1: I think it is because initially, it was taken as a religious practice for the Muslims.
27. I: Okay, what are the chances of someone who is non-Muslim undergoing circumcision compared to one who is a Muslim?
28. R1: Umm, most people say that the healing process is faster for medical circumcision and the pain is also bearable plus, it is also free. On the other hand, the Muslim circumcision requires you to pay something before you are released to go home and so you would need your parents to help you with that unlike the medical circumcision.
29. R5: Just to add, those who have undergone medical circumcision heal quickly whilst those who have done *jando* [term for circumcision done by Muslims] heal after 2 months or even one month. As such, medical circumcision is better.
30. I: Okay, so the healing process is faster, you do not have to pay; what else have we heard about medical circumcision?
31. R6: On the same issue of diseases, I heard that if you are not circumcised, there is a chance that you can cause cervical cancer to the woman you have slept with. If you are circumcised, you avoid that.
32. I: Okay, so that is another benefit.
33. R6: Yes.
34. I: Okay, what else have we heard on medical circumcision?
35. R1: Another benefit of medical circumcision is that the tools they use when circumcising this man are not the same tools that they will use for this one. That will help so that we do not share infections. With *Jando* however, they can use the same tools for two people and that puts people at risk of transmitting diseases.
36. I: Okay, that is another benefit. We have only spoken of the benefits though, what are some of the negative sides of medical circumcision?
37. R3: From what I heard, some people say that there is a risk of dying after circumcision. If your parents do not treat your right, they hurt you in the process and you can die early. Some people are scared and they say that ‘when you are circumcised, you spend three months nursing the wound’. So, the thought of being down for three months makes people worry to say ‘I sleep with different people so for me, staying without sex for three months would be hard’. That is how they reason, but that is not the case.
38. I: Okay, so they worry that the healing takes long and that would mean that they wait that long before they can sleep with a woman.
39. R3: Yes!
40. I: Okay, what else have we heard?
41. R2: I like to follow and learn about circumcision because I have not undergone it. I hear it has its advantages and disadvantages and so when we are in a group setting like this one, I like to hear about it so that maybe, I might also have it done.
42. I: Okay, so you have heard of its advantages and disadvantages right?
43. R2: Yes.
44. I: Tell us some of the disadvantages you have heard.
45. R2: Some people say that it depends on the kind of blood that you have. If your blood does not agree with the circumcision, your body might react and you might not heal as required.
46. I: Okay, if your blood does not agree with the circumcision.
47. R2: Yes.
48. R1: I also heard that its disadvantage is that… I will start with the advantage, the advantage is that you might not feel pain as the circumcision is being done but after some time, you feel so much pain that you fail to walk or do chores and that might be a problem.
49. I: Okay, where is the problem coming in?
50. R1: During circumcision, you do not feel pain. However, when you get home you might not be able to walk around because of the pain coming from the wound.
51. R5: For a grown married man like me, it is a bit hard to undergo circumcision. If I get circumcised, I would need to stay home. The problem with that is that I am the provider of the home and so if I am just staying at home, my children will suffer. I am saying that because I have seen that happen to a certain person. He was being fed like a dog on the chain, being given water and everything else. So, I would like to get circumcised but I take a step back because I think of all that and I how it would affect me.
52. R1: Yes.
53. R5: In my case, I think about it but I get discouraged because of what I have seen.
54. I: Okay, it looks like you have something to say?
55. R6: Just a comment, what he is saying is true. Some of us know about circumcision, but we have known it a bit late, while we are already old. So, with the responsibilities that we have, we know that if we get circumcised, we would need to sit down and we would not be able to go out to look for food and so our families would suffer. The desire to get circumcised is there, but once you are grounded at home, the children will sleep on an empty stomach.
56. I: Okay, number 4, I have not heard you speak for some time now. What do you say?
57. R4: [Chuckles] that is really the disadvantage, it takes time before the wound heals. For someone involved in hard labor, that does not work because they would just be seated at home waiting for the wound to heal. That is my comment.
58. I: Okay, is there anything we would like to add there?
59. R3: Mine will be in form of a question. Some people say that…if you are married and you get circumcised, how long would it take before you sleep with your wife? Maye it depends on when the wound heals?
60. I: Okay, who can help me respond to that?
61. R5: It would not be possible for you to sleep with your wife before your wound heals. That would be making the wound worse. Even if you use a condom, you will still feel pain at some point and you would bruise your wound making it worse. Instead of healing, you would just be making it worse than before.
62. R1: I also have a question… [speaks at the same time as number 6]
63. I: Sorry, let us start with him then we will come to you.
64. R6: Another thing that I think of, I have never experienced it, I just think of it. For men, our sexual desires are always there, they never go away. When that happens, we erect. So, with the wound that you have…besides that, we are also married and we are used to sleeping with our wives. Even if we do not erect at night, in the morning, we wake up and you find that you are already erect. With that, I think that the pain would just be too much.
65. I: Okay, you also had something to say.
66. R1: I had a question, I hear that with medical circumcision, the older you are, the more mature your penis is and I heard that the healing takes longer. Is that true?
67. I: Has anyone else heard of that before?
68. R5: I started thinking about circumcision when I had sores in my penis, small sores. When those sores burst, the wound was quite big, it covered the whole inside and I started to regret that I was not circumcised. To respond to what he said, I had sores and I was erecting but apart from that, I had just taken a new wife. I had sores and my wife also wanted me to make her pregnant and for me to have sex with her often. Not knowing what to do, I still had sex with her but the wounds never healed because in the process, I was bruising the wounds. However, because my wife was insisting, there was nothing I could do, I still had sex with her. The wound really troubled me and I told myself to just get circumcised to deal with it. Although there was that advantage, it would mean that I stay home for the whole day depending on when my body heals. That is the advantage but the disadvantage is that it takes time for you to heal and the family might suffer.
69. I: Alright, thank you very much. Thinking about it, if you were to get circumcised, would you tell your friends or other people?
70. R1: You would tell the ones you are really close and open with that you have been circumcised.
71. I: Okay, you would disclose to those you chat with.
72. R1: Yes, I would tell them.
73. I: Okay.
74. R3: I would tell my friends who are often discouraged by what they hear. I would tell them what I know what happens after circumcision so that they are also encouraged to undergo VMMC.
75. I: Okay, what do others say?
76. R2: Just to add, you need to tell your closest friend so that if anything happens, maybe if the wounds do not heal in time and you need help coming to the clinic, you need to tell your friend so that they can escort you to the clinic to meet a doctor.
77. I: Okay.
78. R5: It is not right for you to tell your friend because after you are circumcised, they will know that the reason you are not up and about is because of the circumcision you underwent. They might come to visit you and when they find you looking unwell they take that and start telling other people. When people hear it, they will start saying that ‘that man is stupid, why would he do that’. It is better to tell him after you have healed and not right after circumcision. You cannot come from circumcision and start saying that ‘I went to get circumcised so when you see me looking like such that is why’, you cannot. They may say bad things about you and you might fail to heal in the end.
79. I: Okay, let us start with number 4 then we will go to number 6.
80. R4: I will also add to what he is saying. It is not right to tell your friends because when you do, with the amount of time you spend at home, your friends might be discouraged to come for VMMC.
81. I: Okay.
82. R6: I feel it is right for you to tell your friend what you have gone through. That is because there are people who have undergone VMMC just that they did not disclose it. When you tell them, they might open up and tell you how they went through it and you would benefit from that.
83. I: Alright, I understand. Let us now move on to the strategies we want to try at this clinic. I will explain each strategy to you and I want to hear your thoughts on each one. First, we would like to conduct regular and more detailed education on circumcision. How do you feel about receiving education on medical circumcision at this clinic?
84. R5: The detailed education on circumcision from the hospital is good because you receive what you need to hear. If you heard it from a friend, you would not really know anything solid to convince you.
85. I: Okay.
86. R3: It is good because when learning about circumcision, we need to know the whole truth and not what we have heard before. We then need to take what is taught and share other people and we would be sharing things that we actually know because we have learnt it.
87. I: Okay, what do others say?
88. R4: It is good to have the education because you will make decisions based on what you know. If you have not been taught, you hear what people say and you tend to get scared. When you are taught however, the fear is removed since you are now clear on what happens.
89. I: Okay, what do others say?
90. R6: I have a question, this education will only be done here at the clinic or you will also go into the different locations or even the schools and where people gather.
91. I: Okay, I will turn that question back to you to say which form of education do you feel would be more effective to you?
92. R6: For me, I think both of them would work but they need to be done simultaneously. When people come to the clinic, they need to be taught. The people who come here come because they are sick. But, there are other people who are not circumcised and they are not sick so they are home or they are school. Then you would need to go to the schools to teach them and they will be compelled to come to the clinic. We are only here at the clinic because we have a problem.
93. I: Okay, so they should be done simultaneously; here at the clinic where people mainly come because they are sick and in places like schools. What do you think would happen if the education were only provided here at the clinic?
94. R5: Most people will not know. As my friend said, if not done in the schools or other places in the communities, most people will not know. They will only know if they come to the clinic. We are receiving this information because we have come to the clinic. As my friend has said, this education should be provided simultaneously. We the people who have come to the clinic might spread the message but the people in the community will not be convinced.
95. I: Okay, they will not be convinced if you tell them?
96. R5: They will not be so convinced.
97. I: Why is that?
98. R5: Because we do not have the resources that you have such that if we told them, they would just say okay and it ends there.
99. I: Why?
100. R5: Because even in our case, we came here, you explained to us, gave us the forms and signed and in the end, we have understood. Similarly, if you were to go to the locations, you would need to carry your forms, read them to the people and inform them. From that, they can make a decision out of understanding.
101. I: Okay, number 6, you also had something to say.
102. R6: I have forgotten [chuckles]
103. I: [chuckles] okay, while we wait for him to remember, what do others think of providing the adequate education? The entire line at the back has been quite. Number 2, what do you say?
104. R2: This education should reach every place. We know of this because we were sick and came here to the clinic and here we are. Those who are in the village would not know what is happening here. You need to make time to come to the communities and let the people know what is required. In that way, the education would be effective.
105. I: Anything to add?
106. R3: Yes, just to add on the benefit of spreading such a message, there are people in the communities who are sick but are unable to come to the clinic. So, if you have a special day to discuss these things, you teach each other a lot and people learn about circumcision. Especially in the schools, you need to make time to teach other people about this.
107. I: Okay, we have talked about adequate education, but what information should be included
108. R5: The information that will be given both here and in the communities should be on the advantages of circumcision, so that the young men realize the benefits. Explaining to them that if circumcised, this is the benefit that is there. You can even ask them some questions and if they fail to answer, you can come in and explain to them.
109. I: Okay, he says we need to explain the benefits of circumcision.
110. R4: The benefits of circumcision.
111. I: Okay, you also think the benefits of circumcision should be included?
112. R4: Okay.
113. I: I was asking what kind of information should be in this education, he has talked of the benefits of circumcision, what do you think?
114. R4: Oh okay, the information on circumcision should be made accessible to everyone who comes here. There should also be a way of making it accessible to those at home.
115. R3: ….
116. I: Sorry, he raised his hand earlier so we will start with him and then come to you.
117. R3: Okay.
118. R6: This information should not only focus on the benefits. They will be interested to come after hearing the benefits, but once they arrive and they experience the bad things, they will go around saying that ‘these people are liars. They only speak of the benefits and not the disadvantages.’ Instead, you should tell them that these are the benefits and these are the disadvantages. When the person is making the decision, they should know that I am going to get circumcised and these are the benefits I will experience and these are the negatives.
119. I: Okay, so they should know both the …
120. R6: They should know everything so that if something happens, you are not taken as the bad ones. Rather, their reaction should be that ‘I already knew about this.’
121. I: Okay, I see. Number 3.
122. R3: Yes, juts to summarize, the information should have both the advantages and the disadvantages just like he said. When the person is coming to the clinic, they should be aware of all things. But, we should still talk of the benefits because like we said, you prevent diseases by getting circumcised.
123. I: Okay, what kind of information should not be included?
124. R: [Silence]
125. I: There are things you hear and you know are a complete waste of time; what kind of information do you feel is unnecessary.
126. R4: There is none.
127. I: [Laughs] that is number 4.
128. R1: Yes, there is none. All the information is necessary.
129. I: Okay, even if we stand there for hours teaching, you think everything is necessary?
130. R1: [Chuckles] yes.
131. I: Number 2, what do you say?
132. R2: All the information is useful.
133. I: Okay, let us move on to the second strategy. For this one, say you have come to the clinic today and you have agreed to undergo circumcision, they give you an appointment date. So, after you have been given the appointment date, how would you feel about receiving an SMS from a healthcare worker at this clinic to remind you about an appointment at the VMMC clinic?
134. R3: I would feel good because normally, we forget. They can give us an appointment date but because we have a lot to do in the communities, we can easily forget. However, when they send you an SMS, they can easily remind you on what you need to do.
135. I: Okay, so in case you have forgotten they would remind you.
136. R3: Yes.
137. R5: They can tell you to come on a Wednesday and before that day comes, you receive a reminder telling you to come to the clinic without fail. If you are far from the clinic, maybe you are in Blantyre for instance; you can easily respond to that message and ask to reschedule your appointment.
138. I: Okay, so that is a benefit of these SMS reminders.
139. R5: Yes.
140. I: How do others feel about receiving these SMS reminders?
141. R1: It is good for them to remind you before the day has arrived. It might happen that you do not have money to come to the clinic and so when they remind you, you can start making plans to find transport money to use to go to the clinic and back. You cannot walk home after undergoing circumcision. That would be hard.
142. I: Okay, [interruption]. Okay, so it would help you plan. What do others say?
143. R: [silent]
144. I: Number 6, I see you are smiling, you have a response.
145. R6: It is quite good because they are reminding us. We stay in different areas and you can agree to go to the clinic but as the day comes, you might start to reconsider your decision of going to the clinic. After you receive the reminder, you can get encouraged and even though you changed your mind, you can still decide to go to the clinic.
146. I: Okay, number 4, do you have anything to add on this issue?
147. R4: No.
148. I: Okay, we are all speaking of the benefits of this strategy but let us also think of some of the disadvantages that would be there.
149. R1: The first disadvantage would be that some of us do not have phones. Apart from that…how can I explain this… someone else can explain
150. R3: Let me just continue from what he was saying, the disadvantage of sending the SMS is that in the communities, there are a lot of connection [network] issues. They can send the message but it will not reach the other person. It can also happen that you have forgotten about your appointment and you are having network issues or your phone is off because not everyone can afford to have money to charge their phone all the time and others actually do not have phones. In that case, you would miss your reminder and that is the disadvantage.
151. I: Okay, for those who do not have phones or for areas with network issues, what do you think could be done?
152. R5: If we have friends who have phones, we can take their phone number and leave it at the clinic. Then you will inform the friend to say ‘you might receive an SMS from the clinic reminding me to go to the clinic on such a day.’ When the message comes, you can boldly come to the clinic.
153. I: Okay, number 3, you also had something to say.
154. R3: Aaa no.
155. I: Okay, anything to add there?
156. R4: No.
157. I: What if someone read the message, how would you feel?
158. R5: It would be wrong if you did not tell them.
159. I: If you did not tell them that what?
160. R5: That you gave their number to the clinic team. If you have not told them, they might receive the message and just ignore it.
161. I: Let’s say the phone is yours but when the message was coming in, the phone was not in your hands, how would you feel?
162. R6: I think there is no problem because these things are not shameful. So whether your friend or your wife sees the message and you told them about it already, there would be no problem and they would inform you after the message has arrived.
163. I: Okay, according to our culture that would be okay?
164. R6: Yes, it is okay.
165. I: What about according to the different religious beliefs.
166. R4: It is also fine.
167. I: Okay, any other thoughts?
168. R6: The point we talked of at the start, there are other people who think of circumcision as something Muslims do. To remove such thoughts, if possible, you can also go through the religious leaders so that they also spread information on circumcision whether they are Muslim or not. By doing that, thinking of circumcision as a Muslim thing will no longer be there.
169. I: Okay, so passing through religious leaders. Just thinking about it, how much people would fail to go for circumcision because of religious issues
170. R5: There are a lot of people who would fail to come. First, it is because they do not know about the circumcision. It is possible that they have heard about it, but because of their religion, the do not really know of it and they do not receive the information. So, it is hard for someone to decide to undergo circumcision if they do not know about it. But, as my brother number 6 has said, it is also important to spread these messages in the church so that everyone knows of it. Some women are also encouraging. When they get home, they can remind the husbands to say ‘did you hear what the pastor or elder said’. As men, we can easily forget, but the women remind us and they even wish for the men to be circumcised.
171. I: Okay, are there any additional views?
172. R: [Silence]
173. I: Okay, how would you feel about receiving money after undergoing circumcision so that it helps with transportation? I remember number 1 said that after getting the SMS reminder, it would help him plan to find transport to come to the clinic. So, what do you think about getting money at the clinic to help you with transportation?
174. R5: ….
175. I: Sorry, let us start with the back line and then we will come back to you.
176. R5: Okay, let them start.
177. R4: It is good because with a wound, it would be hard to travel and as I said, you are not supposed to engage in hard labor after circumcision, so it would be good.
178. I: Okay, I would like you to explain your response further. You have said it is good because you do not need to engage in hard labor after circumcision. So, what would be the benefit of getting this transport money after undergoing circumcision?
179. R4: Okay, what I mean is that it will be easy for you to travel.
180. R1: I think…
181. I: Sorry, number 3 also raised a hand, hold that thought for a bit.
182. R3: Yes, I felt that he was not saying it all. The main advantage would be transportation. Some people who come to the clinic live far. They might undergo circumcision but for them to go back home, it would not be easy. So, the money would help them travel with ease.
183. I: Okay, number 2, what are your thoughts?
184. R2: Mm, let them speak first.
185. I: Okay, number 1.
186. R1: Yes, some people do not make enough money to travel from their homes to the clinic. They would need to borrow money to come here and when they come here and they get the refund, it would be of great help to them and they would pay back the money they borrowed.
187. I: Okay, it looks like we are all saying it is good. Number 5.
188. R5: It is good and it is possible. However, for a married man like me, it would not really work since I would still need to cancel some plans. It is possible for me to come here and they give me a refund afterwards but still, my family would need to eat. That is what would hold me back as a married man. For the young people however, or for the ones who live with their parents, it would be easier.
189. I: Okay, so the problem is still what you will eat after the circumcision.
190. R5: Yes. I come here, you give me transport and I get a bus home. Once I get home, I am grounded and that is where the problem is.
191. I: Okay, what do others say?
192. R6: I think it is good for them to provide transport money, it is good. However, I feel it is also not good. I think that it would be better if they refunded the money you used for transport when coming to the clinic but when going back home, they should drop you off. I am saying that because there are a lot of things that can happen. From here, you have undergone circumcision and you are in pain, then you get on a bus without any problem. From where the bus drops you off, you would need to get a bicycle in order to get home and with the potholes that are along the road, that would be painful. So, it is better if they give you the money you spent when coming to the clinic but they should drop you off at home afterwards.
193. I: Okay, he has brought in the issue of being dropped at home afterwards. What do others say?
194. R1: what he is saying is true.
195. R6: Let me also add on; when they drop you off, they know where you live, they will see where you live. If anything is to happen concerning the wound, they will not struggle to find you with assistance because the one who dropped you off knows where you live.
196. I: Okay, what are the other thoughts on transport reimbursement after circumcision or what would be the right amount of money?
197. R5: The amount would depend on where you are coming from. In my case, I live in area 24 and total transport cost is k400. When I get off the bus, I take a bicycle and that costs k500. So, you would just add the total costs.
198. I: Okay, so the amount will depend on where you live and how much it costs.
199. R[Chorus]: Yes.
200. I: Okay, most of us have agreed to that.
201. R3: Just to add, it should depend on where the person lives. In my case for instance, I live in Mchezi. They can give me k1000 for instance but the transport money would be finished before I get home. So, it should really depend on where you live.
202. I: Okay, if people in our areas or communities heard that the clinic is giving refunds, how would they react?
203. R6: People would have different views. Some would think it is good while others would say that ‘because you have been circumcised, you have sold your foreskin’. They would think that the government has come up with this system as a way of buying your foreskin.
204. I: Okay.
205. R5: The main issue is what I said earlier that once you get circumcised, you are not supposed to tell your friends. By telling them, you bring different issues on yourself. I saw this once back then, I have forgotten who they were but that was when the HIV self-test kits were introduced. They told us that we were free to take the kits home and bring them back after doing the tests. They said they would provide transport and after explaining, they gave me k1000. They said when I come back with my results, they would give me transport money. That was for me, I knew what happened at the hospital and it ended there. In the same way, there is no need to tell people that ‘I went for circumcision and they have given me transport money’, no, there is no one who is supposed to know. People will start saying that ‘the hospital has made this a business, why did they give you transport money, you are stupid’. In the end, adding on to the pain you are already feeling after circumcision, you might just hit them with a stone [out of frustration].
206. I: [Chuckles] okay, so you are emphasizing that there is no need to tell anyone.
207. R5: Yes.
208. I: Okay, number 1, you had something to say.
209. R1: What I wanted to say has already been raised.
210. I: Okay, the back line, what do you say?
211. R4: A lot has already been said, we might end up repeating the very same things.
212. I: Okay, apart from thinking that they have bought the foreskin from you by giving you the money...
213. R5: We call it the hat.
214. I: Okay, the hat, what else would people think?
215. R1: Apart from them thinking that you sold it, they would also think that you [hospital personnel] are doing that in order to make a lot of people get circumcised. That would raise questions of what you want to do with all the skin and that would make most people scared. In short, what should happen is the same as what I saw when I was living in area 12. They were simply spreading the news and those who wanted willingly went, there was no money given. A car was picking them up and dropping them off, giving the money would bring in wrong ideas in the people.
216. I: Okay, you are agreeing with what he raised on providing a car and not giving money.
217. R1: Yes.
218. I: Okay, number 6, you wanted to say something earlier.
219. R6: You are asking what would happen if the people realized that there is transport reimbursement after the circumcision.
220. I: Yes.
221. R6: There are two things that would happen; some would be interested because it would mean travelling to and from the clinic will be easier. Because of the money, people will come. On the other hand, it would also make people scared because they would think that the money is not being given in vain. They would think that the money is coming in as a way of getting a lot of foreskin from people.
222. R5: The main issue is that when you come to the clinic, if you have given someone transport money, you need to talk to them to say ‘we are giving you this money so that you can use when going back home’, do it secretly. You can ask them where they live, and how much money it would cost for them to go back and then you give it to them. There is no need to tell people about what is happening in terms of the money.
223. I: Okay, I will also ask about the different religions, if they knew of this, that people are receiving transport money after undergoing circumcision, what would happen?
224. R5: I think it would be the same.
225. R6: Religion?
226. I: Yes, what would happen with the different religions that we have if they learnt that there is transport being reimbursed.
227. R6: It would depend. There are some religions that are not very strict. But, religions like… I do not know if the Muslims make the people pay after the circumcision or if they are the ones who give out the money. If people pay them to get circumcised, they will not take this well because it would seem like there is a competition. For the other religions that are not against circumcision, they would not react.
228. I: Okay, any other thoughts number 3?
229. R3: No.
230. I: Okay, what are your thoughts about using education, SMS reminders and receiving money in combination?
231. R5: All of them are necessary because people who did not want will be encouraged, those who thought about it but were afraid will also be encouraged. Maybe they thought that things would not go well if they come to the clinic, they will be reminded. I think these things should go together; transport, Education and SMS.
232. I: Okay, number 5 says it is good.
233. R6: The strategies are good and it would be good for them to be done at once. However, just my thoughts…
234. I: Yes, we want your thoughts.
235. R6: Alright, there are other people who are married and they care for two children and the wife. If that man is at home nursing the wound, he will still be asked to provide something to eat. So, if it were possible, there should be a difference between the one who is married and one who is not married. For the married one, you would need to provide some money [chuckles] to help him whilst he is not working.
236. I: Okay, so the difference between one who is not married…
237. R6: Yes, the difference is that the one who is not married can sleep all day and they would still have something to eat because the parents will provide. For the other one, because he is sleeping at home, the children will not eat.
238. I: So this difference is not on the transport being reimbursed but this one who is married should be given money on top of the transport?
239. R6: Yes, if it confirmed that the person will be reimbursed, because initially my thoughts were that they should only be given the money they used to come to the clinic and they should be dropped off after the circumcision. However, if transport was being provided, and this person has responsibilities and there is proof of those responsibilities, that person needs to be assisted so that while he is at home, he should be able to eat. Because for you to have that wound and also be hungry at the same time, eeeh [chuckles] that would be a problem.
240. I: Okay, number 3.
241. R3: Yes, just to add, the strategies are good but we really to stress on the financial support for the family. They can be reimbursed the transport, but the main problem is how they will sustain their household. The transport reimbursement will only be useful for that same day and yet they will have to stay home for several days. Those other days are the ones that would be problematic. However, these strategies are good.
242. I: Okay, number 2?
243. R2: I feel that transport reimbursement is not a good strategy because people reason in different ways. As we already said, they would think that you are benefiting from our foreskin. But as he said, it would be better to pick the people up and drop them after they have undergone the circumcision. I feel that would work better in terms of the transport.
244. I: Okay, is there anything to add
245. R: [Silence]
246. I: [Chuckles] alright, I have one last question and everyone will have to respond to this one. Of all the strategies we have talked about; the SMS reminder, transport reimbursement and education; if you were to choose one or two which you feel would be the most effective, which ones would you choose and why? Everyone will respond to this one; where do we start?
247. R3: For me,
248. R1: Ask the question again.
249. R3: No, I have understood the question.
250. R1: He is just coming back.
251. I: Okay, I will ask the question again, lets let him finish speaking first before he forgets.
252. R3: Okay, I would choose the intensive education and transport reimbursement. That is because what you learn from the education will be true and not mere hearsay as it is in our communities. The benefit of the transport reimbursement is that it would help in making travelling to and from the clinic easier since you have a wound.
253. I: Okay, the question I asked was which strategies you would choose from the 3 we have discussed which are; education, SMS reminders and transport reimbursement, if you were to choose to combine two strategies or if you were to choose one which would be more effective, which one would you choose. Number 3 started and number 4 also has something.
254. R4: For me, I would choose transport reimbursement and SMS reminders.
255. I: Okay, why?
256. R4: Because the SMS would remind me if I have forgotten or if I am lazy because of the pain coming from the wound.
257. I: Okay, but remember that this message is coming in before you go for circumcision, it is reminding you of your appointment.
258. R4: Oh okay, then it is good because it will remind me if I forgot or if I was reluctant. The SMS will remind me.
259. I: Okay, and the transport?
260. R4: Yes, the transport will help me go back home easily after the circumcision.
261. I: Okay, 4 of us are left, which ones would you choose?
262. R: [R1 and R5 together] I would…
263. I: Let us start with number 1.
264. R1: Yes, I would choose the SMS reminders as well because when I receive the message that I need to come for circumcision, I would easily borrow money to come to the clinic because I know they will refund your transport. You would borrow the money, come here for assistance and then go back home.
265. I: Okay, number 5.
266. R5: Yes, what I wanted to say is the same thing number 1 has said. When you receive the SMS, you are reminded and if they are refunding transport, they are also encouraging you and removing any other thoughts that you had on how you would get home after the circumcision. You would not have any of those worries anymore.
267. I: Okay, so you are also agreeing to SMS remembers and transport reimbursement.
268. R5: Yes.
269. I: Okay, what would we choose?
270. R6: I would choose the education because there is the advantage that when one has come to the clinic and they are not yet circumcised, they will be taught and they can make a decision. When you are done, you can take the message to other people. I would also choose the one… what are the others?
271. I: There is SMS and transport reimbursement left, do not feel pressured to choose two.
272. R6: I have chosen the education then.
273. I: Is it because I told you not to feel pressured [chuckles]?
274. R6: I feel that the transport one would be better if they dropped us off after the circumcision. They should only give me the money I used when coming to the clinic.
275. I: Okay, you are the only one left number 2.
276. R2: The message is a good strategy because if you forget, you would be reminded. Secondly transportation is also important, like the dropping off at home needs emphasis.
277. I: Okay, on being dropped off, he said it is to avoid discomfort on the bike going home. Do we agree with the response?
278. R3: Yes, we agree.
279. R1: There are two reasons; first is because you will picked from your clinic to your home and secondly, giving out money would bring wrong ideas.
280. R5: I just want to add on the same transport. It can be provided, but the number of people who need that transport might stay far apart from each other. One in Chigwiri, the other in Mchesi, and etc. for all of those to be dropped off, the last one will get home late. So, the best way is to provide money and find out how much transport costs.
281. I: Okay, looks like you have something to say.
282. R6: Yes, let me add. When they were bringing in these strategies, they knew that we do not all stay at the same place. There was a time when circumcision was being done among the youth where I live. They would ask them to say ‘how many of you live this side’ and they all go into one car and they dropped them at their houses until the last one. It happened that way. Because our bodies are different, some may get circumcised and their penis starts to swell. The advantage in being dropped off is that when that happens and you cannot even get on a bicycle, you will call them and they will easily find where you live because they have been there before.
283. I: Alright, is there anything we would like to add?
284. R: [Silence]
285. I: To add to our discussion and the issue of circumcision, anything we would like to add.
286. R: [Silence]
287. I: Or any questions.
288. R3: My question is that because we are learning, I would like to know if it is true that you can stay 3 months or two before the wound heals?
289. I: Okay, I have heard your question. Is there any other question?
290. R2: My question is that now that we have had this discussion today, will they continue or this is the end?
291. I: This is the end, they are for one time only.
292. R1: To promote VMMC, it is supposed to be done even in the private hospitals for free.
293. I: Okay, anything else?
294. R: [Silence]
295. I: I take it there is nothing. This is also the end of what I had but I really appreciate you for your time today right?
296. R: [Silence]
297. I: Right?
298. R[All]: yes, thank you.

THE END
