# Supplementary material for: Acceptability, feasibility and appropriateness of intensified health education, SMS/phone tracing and transport reimbursement for uptake of voluntary medical male circumcision in a sexually transmitted infections clinic in Malawi: A mixed methods study
Source: PLoS One. 2025 Jan 24;20(1):e0301952. doi: 10.1371/journal.pone.0301952 (PMC11760565; doi:10.1371/journal.pone.0301952)
Supplement: S1 Data — (ZIP) [file pone.0301952.s004.zip › Qualitative data/Baseline FGD Transcripts/FGD2_Transcript.docx]

1. I: To start, we are talking about circumcision, tell me what you have heard about medical circumcision? Anyone can start; let us just remember to mention our numbers.
2. R6: I heard that medical male circumcision helps with preventing diseases like cancer for men and cervical cancer for women. This circumcision helps with these two things.
3. I: Okay, he has talked of preventing diseases like cancer, what else have we heard?
4. R4: It also helps with hygiene for the penis. As such, it is also, what makes it possible to prevent different diseases by 60%.
5. I: How does this hygiene come about?
6. R4: This hygiene because at the tip of the penis, there are things that are powder like. When the man has sex with a woman, he leaves those things at the cervix of the woman. When the things stay there for a long time it leads to cancer. However, when one is circumcised, those things are not there.
7. I: Okay, he has talked of hygiene, what else have we heard about medical circumcision?
8. R1: When you have been circumcised, like they said, you reduce your chances of getting diseases by 60%. As such, when having sex, you cannot easily contract diseases. When you are circumcised, the tip of your penis is as dry as the skin whilst if you are not circumcised, because of the skin covering your penis, it is soft on the inside and the bacteria can easily live there.
9. R6: [Raise your voice we cannot hear you.]
10. R1: [Maybe I should take off my mask.]
11. I: [Just carry the recorder in your hands, let us make sure it gets your voice]
12. R1: They said that when you are circumcised, you cannot contract diseases; your chances are lowered by 60%. The remaining 40% is if you have a wound on your skin. However, if you are circumcised, you do not have that wound anymore. That is what I was saying.
13. I: Alright, thank you.
14. R2: I just wanted to add on to that; when the foreskin has been cut off, the tip of the penis is usually dry. If the foreskin is still there, the tip is very soft such that when you have sex with a woman, the skin covers the penis and everything is stored inside. Bacteria is even found there and that cause several diseases. If it is dry however, bacteria cannot survive and so it dies. In that way, you are protected. That is why they say that you cannot contract diseases or that you reduce your chances of contracting diseases by 60% if you are circumcised.
15. I: Okay, apart from what we have heard today, was today the first time we have heard about circumcision?
16. R[All]: [Silence]
17. I: Was it our first time hearing about circumcision?
18. R[Chorus]: No.
19. I: Okay, in the communities where we live, what else do people say about medical circumcision?
20. R6: They say that one who is circumcised is a man. They say that when having sex with someone who is circumcised, the woman feels good. Adding to that, they say that the person cannot contract diseases easily. For one who is not circumcised, the women say that they cannot compare to one who is circumcised. In addition, in the communities, most people say that the circumcised person is better.
21. I: Okay, he has talked about it in terms of sex; what have others heard?
22. R5: In our communities, the issue of circumcision is controversial. People lie to each other and say that it is not good because sex is not enjoyable and some say you can still contract diseases even after you are circumcised. That makes people reluctant when in terms of whether to go through with it or not. Had it been that we come to the clinics, we would have known and heard of the benefits of circumcision like the ones we have heard.
23. I: Okay, I stopped you the other time, number 3.
24. R3: Some people are scared of circumcision, specifically those who live in town. Some have to wake up early in the morning to go to their businesses. They rely on what they earn each day for them to eat. So, most of them are scared that if they are circumcised, they would have to stay home, like they are sick and during that time, their households would have no food to eat. Most people in the communities are afraid of that. That they will have a wound and they will be sick for a long time and in that time, their families would have no food.
25. I: Okay, you also had something to say.
26. R5: It has already been mentioned.
27. I: It is what he has said?
28. R5: Yes.
29. I: Okay, the line at the back, I have not heard from you. What have you heard about circumcision?
30. R9: In terms of circumcision, the main thing we have heard in our communities is hygiene. The other thing is that you do not contract diseases easily. That there is a 60% chance for you to contract diseases unlike someone who has not been circumcised.
31. I: Okay, what else?
32. R7: I heard that when you are circumcised, it is very easy for you to impregnate a woman, unlike someone who is not circumcised. That is because the foreskin helps to hold back some sperms so that they are not all released at once when you are not circumcised. When you are circumcised, everything goes out at once with full force. That is what I heard.
33. I: Okay, is there anything else?
34. R6: Some are afraid of being circumcised because following cultural beliefs; they say circumcision is for Muslims only. Some are reluctant and they say that they cannot get circumcised because they are not Muslims.
35. I: Okay, others?
36. R2: Some people use their religions and are afraid to go through with circumcision while others use their culture or the areas where they come from. The *Tumbuka* for instance are not into circumcision whilst most Yao get circumcised a lot. The other thing is that some people worry to go through with circumcision whilst they are married. They say that ‘I am married and what if my wife will want to have sex with me and yet they are given a long time period in which I cannot have sex with any woman’. They worry that the wife might start having other affairs. Out of jealousy and fearing that the wife might start sleeping with other men, people are afraid to come for circumcision.
37. I: Okay.
38. R4: Another thing that I have heard in my community, most people say that it is hard for someone to come for circumcision because here at the hospital, when you cut off the foreskin, you do not give it back to the person or show them where you go with it. The thoughts that we have are that you use that foreskin and earn money out of it. That worries the people in the communities, for them to come to the hospital and get circumcised. As such, circumcision is taken as something that is wrong because some people use it to earn money.
39. I: Okay.
40. R8: In the communities, other people are also afraid of the circumcision because the wound takes time to heal and it just gets bigger. In the end, we heard that someone died because their wound took time to heal.
41. I: Okay, so it just grew bigger.
42. R8: Yes.
43. I: Okay, I see there are many things being said. What else have we heard; if there is anything else?
44. R[All]: [Silence]
45. I: We have talked about the benefits where we said it helps prevent diseases, hygiene; he also said that women like to have sex with someone who is circumcised. He also said it is easy to impregnate a woman and we talked about the healing time of the wound and how one cannot walk around as they please after circumcision. Not so?
46. R [Chorus]: Yes.
47. I: Okay, is there anything else we have heard?
48. R6: I also heard that when someone who is circumcised undresses in front of his friends, he does not release any odor because his penis is clean. If you are not circumcised and you undress, when the foreskin is peeled back, a bad odor is released.
49. I: Okay, that also talks about hygiene right?
50. R6: Yes.
51. I: Alright, thank you. How would you, seated here, how would you feel about undergoing medical circumcision? Just your thoughts.
52. R4: For me, from what I have heard about medical circumcision, I think it is wise and I can go through with it. First, I would be protecting my life and the life of the person I would have sex with.
53. I: Okay, that is number 4. What do others say?
54. R1: Just to add to what he has said, those are the benefits and I have made the decision to be circumcised so that I should also be protected from diseases.
55. I: Okay, what do others say? By the way, I do not want all of you to agree, everyone should feel free to voice out their views.
56. R3: As we said, that after circumcision, you stay at home for 3 weeks nursing the wound, from what we have heard that is. For someone who runs their business in town and they travel from their home to go to town every day, how do you assist them?
57. I: Okay, what type of assistance can you think of? As I said, we want to try these strategies; they are not in place yet. So, what type of assistance can you think of for the circumcision to still be done?
58. R3: I would have liked that for someone who [silence] for someone who is a vendor in town, I think that if there was a way so that when the person is circumcised and they are at home, if they heal without any problems and it does not take long, they can go back to their businesses. However, if they are having problems with the wound, they need to be visited and given assistance so that even while he is home, both he and his family should have something to eat until he heals.
59. I: Okay, if he heals without any problem then that is fine but if he is having problems with healing, he should be visited.
60. R3: Yes, he should be visited.
61. I: Okay, that is what number 3 thinks. What do others say?
62. R4: On the same, I think that even for those who go to work, if they do not heal or they are having trouble with healing, they should be assisted with transportation to work. That is because some companies do not easily understand the things that happen to a person. We have companies that are different from the government where if you do not show up to work for three or four days, they fire you. I think it would be wise that after someone is circumcised, they should also be assisted with transportation to where they work so that they can still earn a living for them and their families.
63. I: Okay.
64. R2: I think the medical people should really look into private companies. That is because in those companies, people really face hardship when they have a problem, especially one that they brought on themselves. When you come to the hospital and you volunteer to get circumcised, you really go through with the circumcision, when you face any challenges, the company does not assist you. So, these companies really need to be enlightened on the benefits of this circumcision to one’s life, that would help. Apart from that, there should be a way of helping these people when they are at home. We have talked of people who run businesses, they need to be assisted with something because the only way they have something to eat is when they come to town. If those people are home for 3 days, their household will suffer. If such people were receiving something to help them run their families, that would help.
65. I: Okay, I saw you raised a hand.
66. R10: My thoughts have already been mentioned.
67. I: Okay, what he has just said?
68. R10: Yes.
69. I: Alright, if all these things were considered, let just assume they were considered, what do you think of the numbers of men that come for circumcision; what do you think would happen?
70. R3: It would go up. People would come voluntarily and boldly because they know that if I face any challenge, assistance will be provided.
71. I: Okay, moving on, how do others feel about medical circumcision? My people at the back, what do we say?
72. R9: I have welcomed medical circumcision and I am ready to have it done. I have seen that there are benefits of protecting my life, having a hygiene penis and women would enjoy having sex with me.
73. I: Okay, that is number 9, number 10?
74. R: I will just add to what the others have explained. It is a very good thing to have circumcision. The worries that are there are there because we do not really know what happens. For us, for people who live alone and fend for ourselves, it is not that we do not want to have circumcision; we want to do it because we do not want to contract diseases easily. We just try to have sex once and the next thing we see is we have diseases and we do not want that. We see promiscuous people; they do not contract diseases easily, and years pass by before they contract diseases. For us who are not circumcision, we try to do the same a little but we contract diseases. Our worry is that when we go for circumcision and we have to stay home, what will happen since we are alone. So, we are forced not to go because we are worried. We say that if we go for circumcision, we will suffer for the days when we have to stay home. However, if we do not go, we will continue getting sick each time we have sex. We would like to go for circumcision, the only challenge is what will happen and how we will deal with the challenges that may come.
75. I: Alright. If we went through with circumcision, would we tell other people about it? Be it our relatives or friends.
76. R6: It is right to tell the relatives about it so that if anything happens, they would know what the cause is. Maybe they would even find a way of helping you like taking you to the hospital or finding medication to help with the problem. Unlike not telling them.
77. I: Okay, number 1, you also had something to say.
78. R1: Yes, it is good to tell your relatives or your friends who have not had circumcision. It would be a way of counselling them and telling them the benefits of circumcision so that they can make a decision to get circumcised. You are supposed to tell them and explain to them what you heard at the hospital, the benefits of circumcision.
79. I: Okay, he said they would know what to do if something happened and he says it could also be a way of encouraging the others to get circumcised. What do others say? Would we tell other people or not?
80. R10: It is good to tell our friends and our relatives about circumcision. On the part of the relatives, so that they should help you by bringing you to the hospital if something happens. For our friends, it is good to tell them so that if they are not circumcised, they should get circumcised and reduce their chances of contracting diseases.
81. I: Okay, number 8.
82. R8: It is good to tell them because they are the ones who would help you if anything happens to your wound, because you live with them. Second, you [medical people] and I know each other, so it would be good to tell them so that they are surprised when you come to visit me.
83. I: Okay.
84. R2: It is good to tell them, especially for those who are married so that the wife understands that from this day, there will be no sexual activity. She needs to know that you have been circumcised and she must understand that. In the same way, the relatives should know so that whenever something happens to the wife [if she complains or if she starts having affairs], they should know where that is coming from. That is for those who are married, thank you.
85. I: Okay, what do others say? Number 5, what do you say?
86. R5: I think it is good to tell them because others need to know whether circumcision is really good or not. If you do not tell your friends, they will never know if circumcision is good or not.
87. R4: For me, I think it is not a good thing in a way. For me, it is not good to tell them because; I heard one of us say that others have died because of the same circumcision. It could be that they did not do it well or you are the one who did not take good care of the wound and it became big. It is something that would scare off the ones who have not done it and using you as an example, they would be convinced that circumcision is not good. Sometimes, I think it is not good to tell people. Maybe after you healed and everything has turned out fine, maybe tell them that time.
88. I: [Chuckles] okay, so we should only tell them after healing so that they do not get worried.
89. R4: Yes!
90. I: Okay.
91. R6: It is good to tell them because when they see your way of walking has changed; they will assume you have contracted an STI. Because you did not tell them, they will spread stories and say that you contracted STIs. For you to ask someone out [to date someone], they will turn you down and say that you have STI when that is not the case.
92. R?: [Laughs in the background]
93. I: Okay [chuckles], what do others say?
94. R: [Silence]
95. I: We agree with what is said or that is all we had?
96. R2: I think that is all we can say.
97. I: Alright, [chuckles] thank you. Next, I want us to talk about the strategies we want to try out at this clinic, the ones I mentioned earlier. First, we would like to conduct regular and more detailed education on circumcision. How do you feel about receiving education on medical circumcision at this clinic?
98. R7: I did not get the question.
99. I: Okay, when we started, I mentioned three strategies that we want to try. I talked of Intensive education, SMS reminders and transport reimbursement. I have started with the detailed education and I said that this will be done regularly and that it will be detailed. This will take place here at the clinic. Therefore, what are your thoughts on receiving education on medical circumcision at this clinic; what are your thoughts.
100. R2: Okay [crosstalk with number 1 and 4] [chuckles]
101. I: Let us start with number 2, then number 1 and then number 4.
102. R2: This education is very important because it will remove all the misconceptions or things that are against circumcision. As we said, I said that people think that when the foreskin is cut, the government uses it for other purposes. When this education is there, those views that are against the true aims of circumcision will be corrected because people will know and realize that the aim of circumcision is to guard their lives. Not that the government benefits from circumcision, but it done to guard one’s own life.
103. I: Okay, number 1.
104. R1: I am happy with that because I was one of the people who were afraid of coming. Nevertheless, I have clearly understood the benefits and I am satisfied with them.
105. I: Okay, does that mean you are okay with the education?
106. R1: Very much!
107. I: Okay, number 4, you also had your hand raised.
108. R4: Yes, I equally agree with the education because it will still help. If I have been able to make a decision and I am satisfied from the little that I have heard, if we were to go deep into this study, it means there is a lot that would make someone interested in the circumcision. Things that would teach the person that they need to do the circumcision. I agree with that, and it is a good thing. It will help Malawians become aware.
109. I: Okay. Let us start with 5 then we go to number 9. [starts to rain]
110. R5: Yes, I also think that this education is good because if not, when we tell our friends about circumcision, we will be telling them our thoughts. However, after we are taught, we will tell them what you have taught us, the things we have learnt. In that way, the things we will tell them will be correct.
111. I: Okay, so you will tell the right things because you have been taught
112. R5: Yes, because we have been taught.
113. I: Okay, number 9.
114. R9: This education is good. It is good because we will know the benefits and the disadvantages of circumcision. We will stop listening to what people say because it is all lies.
115. I: I am having trouble hearing you; please also carry the recorder.
116. R9: I have said that it is good because we will know the benefits and the disadvantages. We will learn these things firsthand and we will not listen to the things being said out there.
117. I: Okay, so you will know the benefits and disadvantages firsthand unlike listening to what people say out there.
118. R9: Yes!
119. I: Alright, what do others say?
120. R8: This intensive education is good because it will be happening right here at the clinic unlike just taking you from the villages without understanding anything. After the education, you will be able to understand medical circumcision.
121. I: We are all saying it is good.
122. R [Chorus]: Yes.
123. I: What should be contained in this education? [background inaudible, too much rain][interview paused]
124. I: I was asking what information should be contained in this education. We have said that when a person understands, they will make a decision and those who have heard wrong things about circumcision would understand. However, what should be contained in this education for it to be relevant. Have we heard the question?
125. R3: It is important for you who are teaching these people to enlighten them on the things that happen and on the things spreading in the communities that make them afraid or scared. If you would enlighten them and remove the fear that they have so that they know the benefits of circumcision.
126. I: Okay, he has talked of telling them the things that happen so that the peoples fear concerning circumcision are removed. Number 5?
127. R5: The education also needs to…
128. I: Please carry the recorder.
129. R5: Need to show them videos explaining the things that happen and the benefits and well as the disadvantages. That would interest people and, they would easily understand what the benefits are and the disadvantages are.
130. I: Okay, so using videos so that people easily understand.
131. R5: Yes.
132. I: What other information should it contain? The information we think is relevant.
133. R4: I think there needs to be information on how they can care for themselves after circumcision. If they do not take good care of it, it makes circumcision look bad, as though it is not a good thing. Therefore, it should also contain how they would take care of themselves after the circumcision.
134. I: Okay, what do others say? Alternatively, what information is not important? What information is irrelevant? You have something to say.
135. R?: No.
136. I: Okay, what information do we think is irrelevant? Number 10?
137. R10: No.
138. R6: The irrelevant information would be someone talking about how to take care of teeth.
139. I: We should not talk of taking care of teeth.
140. R6: Yes, the issue is circumcision, so they should not talk about teeth.
141. R[Chorus]: [Laugh]
142. I: [Laughs] I hear what you are saying, so the focus should be on circumcision and not divert to other topics.
143. R6: Yes!
144. R3: The truth about circumcision should be discussed without adding anything extra or hiding anything so that people know the whole truth about circumcision. Tell them everything about circumcision.
145. I: Okay, what do others say?
146. R5: When giving this education, do not tell them that the wound gets bigger and things like that. When you tell them that the wound gets bigger if not taken care of, people will be afraid. They just need to be told that when the wound is taken care of, it does not take time to heal. People would feel good about that.
147. I: Alright, are there any other thoughts there?
148. R: [Silence]
149. I: So we move on.
150. R[Chorus]: Yes.
151. I: Okay, another strategy is receiving SMSs. We have left the hospital, after a person decides to have circumcision, they are given an appointment date. After the person goes home, they are now receiving messages from the hospital as a way of reminding them of their appointment date. How would you feel about this or what are your thoughts? Is this clear?
152. R?: Yes.
153. R7: It is a very good thing because after you have been circumcised and you are facing a certain challenge, it is easy to get in touch with the doctor who sent the message and tell him how he is feeling. In that way, it would help the client save money because if the problem is small, he will easily get assisted. It will be easy to get in touch with the person and it would also be easy to get assistance, you would be able to receive it while you are home. If you live very far from the hospital, you will be able to save money. As such, I think it is very good.
154. R4: I did not understand. Did you say before circumcision or after circumcision?
155. I: Before circumcision. They have given you an appointment date and before that day comes, you are receiving an SMS reminder. I am not sure what it will say, but it will remind you to come for your appointment on such a date. Do you understand?
156. R[chorus] Yes.
157. I: What are your thoughts on receiving such SMSs as a reminder for your circumcision appointment date?
158. R4: It is a very good thing to remind the person because it can happen that he was interested in coming for VMMC, but, because of the things happening around him, he can forget. Therefore, these messages are important because they would encourage the person and if they had any second thoughts, they should no longer be hesitant and they should come to the clinic.
159. I: Okay, number 6.
160. R6: The response I had is the same.
161. I: Okay,
162. R2: It is a very good thing. To be honest, when the person comes to the clinic and is given an appointment date, it is very likely that they would tell a friend to say ‘on such a day, I will go for VMMC’. Among the friends he tells, there are others who can discourage him. In being reminded, he can also get encouraged to come to the clinic for VMMC and not regard the thoughts of his friends who are discouraging him.
163. I: Okay, what do others say?
164. R: [Silence]
165. I: What are our thoughts on receiving SMSs from the hospital reminding us of our appointment dates?
166. R4: I think it is a good thing because you would have the opportunity to ask. Even though you have been educated, we still hear things in the communities and among those things; there may be things that are against what we have heard, depending on the different cultures as well. This can also give you an advantage of asking the doctors for clarity if there was anything discouraging you.
167. I: So you would ask them before you come to the clinic.
168. R4: Yes, before you come.
169. I: Okay.
170. R3: It is true and even the way we have been taught today, when we go home, we can explain to our friends that we came to the hospital and these are the things we were taught. Within the group, some may doubt and they might think we are lying. They might think we have been tasked or hired to coax people in the community. When you send the message and we show them, they can get interested in coming as well.
171. I: Okay, number 10.
172. R10: Umm, when the SMS has been sent, and you are home preparing to come for circumcision. We were told that if we have some wounds, or, if we were not careful and have contracted a certain disease or there is a problem at the tip of the penis, if you have such a problem, you need to excuse yourself and have them give you more days before you can go for VMMC. It would a problem for you to come for VMMC whilst you have a certain disease or you have developed something whilst you were home.
173. I: Okay, and in this case, what will happen after receiving the SMS?
174. R10: When you receive the SMS, you can excuse yourself and tell that you have a certain problem. In that way, you can come to the clinic or they would come to you with assistance so that you get better.
175. I: Okay, what do others say? He also brought up the issue of culture and the different things therein. On the issue of receiving these SMS reminders, how would that sit in with our culture? Alternatively, if people learnt that you are receiving SMS reminders from the hospital to go for VMMC, how would they react?
176. R6: Those who have done it can encourage you, because they know the benefits. Those who have never done it and only hear of its negatives, that it is painful, and others have said people have died because of the circumcision; those would discourage you. For others, because of the belief that the government runs a business with the foreskin, when that is not the case. Circumcision started a long time ago. For someone who has done it, and knows the benefits, they would encourage other people. It would be hard for someone who has not done it to encourage other people. They will ask how you are encouraging people and yet you have not equally done it. It becomes hard to encourage other people.
177. I: Okay.
178. R2: There are wrong perceptions that we have in our cultures as well. If you receive a message and you tell people that ‘I have received an SMS reminding me to go for circumcision’, people will call it satanic with the time that we are. The benefits are there, but the negatives are also there because most people believe that receiving an SMS that is encouraging you to do something is satanic, that they want you to be a Satanist as well. I think that is the disadvantage. The way people in our societies would react; others would have a negative reaction. That is the disadvantage. It is a good thing, but people react the wrong way. They think that the hospital wants to benefit at the end of it all.
179. I: Okay, you also had something to say.
180. R4: I think that in terms of culture, it would be good because others are so settled. For us Muslims for instance, we used to circumcision being part of our culture. Because of that, there may be differentiating or comparing which form of circumcision is better, in case other people heard of it. Comparing which circumcision is better than the one done per our culture. If you got circumcised and everything went well, most people would gain interest and might even stop doing it the cultural way. To add on that, another way would be getting our people from *Jando* and have them get circumcised here and have the ceremony at home, if everything went in order and the healing has gone well.
181. I: You have brought in the issue of religion as well,
182. R4: Yes my religion!
183. I: Would that not raise conflicts? If people are being taken and brought to the hospital for VMMC and then sending them SMS reminders?
184. R4: I think…
185. I: Start, and then 6 will come in.
186. R6: I just wanted to help you respond.
187. R4: I do not think any conflicts would come in, so long as the procedure is being done well. Communication between the two, so that only the circumcision is being done here, it would be a good thing for the circumcision to be done here because they have their way of doing it. I also heard that the way it is done here at the hospital is different from the way it is done at home. That we leave some of the foreskin whilst here, all of it is removed. If there were that opportunity for us Muslims, it would be a very good thing. Again, when we do it at home, there is some level of cruelty when they are doing it whilst here, you would feel good about it and even play while you are being circumcised. So, if there was communication between our cultural leaders and the hospital personnel, I think this would be a good thing.
188. I: Okay, so that the circumcision is done at the hospital and the ceremony is done at home.
189. R4: Yes, the ceremony should be done at home and the circumcision should be done here at the hospital.
190. I: Okay, number 6?
191. R6: I want to add on the same that there just needs to be understanding between the hospital and religious leaders like the Muslims who have places where they also do circumcision. If you people talked and agreed that the hospital does the circumcision and the ceremony is done as they do it, it would be a good thing. If the people came here without the knowledge of the religious leaders, the conflicts would be there.
192. I: Okay.
193. R2: I also think that there would be no conflicts because they all have the same aim, to cut the foreskin. Let us focus on that, cutting the foreskin. The ceremony is on the other side. After the person is circumcised, the ceremony can happen is it does with those who are Muslims or Yao. I do not think there is any problem; they would understand each other. So long as the foreskin has been cut.
194. I: Okay, so long as the foreskin is cut.
195. R2: Yes, that is the issue.
196. I: Okay, anything else?
197. R9: The circumcision that is done culturally and the one done at the clinic. You have said that to be circumcised here, you need to be 18 years old and higher. For the one culturally done, anyone can do it, even children as young as 8 or 10 years old. Would it not be different there; they take everyone while you only take those who are older.
198. I: Okay, so there would be a difference.
199. R9: I am asking if there would be no difference there.
200. I: Okay, who can help me with that one, someone who has understood.
201. R2: Let me help, what he means is that here at the hospital, there is an age range of who can be circumcised; those 15 years old up to those in their sixties. On the other hand, the Muslims can circumcise anyone from the ages of 5 or 6 or 7. Would it work out well there, since there is this difference in who is circumcised between you and them? They take everyone whilst the medical one depends on how mature the person is. That difference would cause some problems in understanding.
202. I: Okay, what problems would come up from that? Sorry, you had something to say earlier and so did you.
203. R2: I just wanted to repeat the question.
204. I: Okay, and there?
205. R: I wanted to respond to his question. When I was being taught yesterday, they told me that medical circumcision is not about age. It just depends on how you have accepted it individually or on the parent. If the parent agrees, they can be circumcised. That is why I think that there is no difference between medical circumcision and the one done culturally. They can take a four year old, just as we do it in our culture, and bring them here for medical circumcision. If there is an age range, this is where I will learn of that. But from what I learnt yesterday, I did not hear anything concerning age. [Number 9 goes to answer a phone call]
206. I: Okay, the one who asked the question has stepped out. Is there anything else to add there?
207. R?: No.
208. I: Okay, is there anything to add on the issue of receiving SMS reminders when we have been given a VMMC appointment date?
209. R: [Silence]
210. I: We continue.
211. R?: Let us continue.
212. I: Okay, the last one is on being reimbursed on the day of circumcision. What are your thoughts on being reimbursed after being circumcised to help with your transportation costs?
213. R6: That is good, especially when you do not have money for transport. Some people live far and it costs them a lot to come to the hospital. It might also happen that the time you remind them to come, they do not have any money. However, this would mean that if the person gets the reminder, they would boldly go and borrow money knowing that you will refund the money.
214. I: Okay, so it will be good.
215. R6: It would be good because they know that when I borrow money, I will refund that money when coming back.
216. R4: It is quite a good thing because of the money, giving people money to cover their transport costs. It would encourage some people to say ‘I am not interested in circumcision, I just want the money. They can circumcise me, so long as they give me the money.’ Therefore, it is a very good thing.
217. R?: [Laughs]
218. I: [Chuckles] okay, number 8.
219. R8: It is good to be refunded transport. That is because you come to the clinic and they circumcise you, you would not be able to walk back home, the same distance you covered when coming. It is better for them to give you transport money, you get a bus and you arrive home in good time. You would not be able to walk home after circumcision. Thank you.
220. R2: It is a very good thing and it is something that would encourage people. There are people who live far; some live in Mitundu, some in Malingunde and they cannot afford to just come for circumcision. However, if they knew that they will be refunded, the people would be encouraged to come here and have the circumcision done and go back home. They fail to at times because the money they have is only enough to take them to the hospital and not back home. Then they worry about how they will get back home especially after the circumcision. It would therefore encourage many people after they are told that they will get a refund. It is the same thing, with what the Malawi Blood Transfusion people were doing. When people donate blood, they were given transport money and in that way, many people went to donate. That is one obvious example, and I think that would encourage people.
221. I: Okay, we are only speaking of the advantages. What would be the disadvantages of this?
222. R8: I think the disadvantage with the refund is that some people might say ‘why are they refunding and yet the circumcision is for your own benefit. Do they want something from all this?’ some people would think in that way.
223. I: Okay, so the negative thoughts would come back.
224. R8: Yes.
225. I: Okay.
226. R1: I think there is no disadvantage.
227. I: Okay.
228. R2: The disadvantage is there because of peoples mentalities. They would think to say ‘these people are buying the foreskin. If they are giving money after circumcision, it means they are buying the skin’. There is no disadvantage; it only comes in because of the way people think. However, for someone who has been taught, there would be no problem. That is why we are having education, to teach the people and after they are taught, they will be aware.
229. I: Okay, what do others say on refunding transport money.
230. R: [Silence]
231. I: For those of us who agree with it, what would be a favorable amount? How much?
232. R6: It would depend on how far the person lives. In my case for instance, it costs me k200 to go where I live. There are some here and it would cost k1000 and others even more than that. As such, for us to put a limit, it would be a problem. Rather, it should depend on how much it costs for someone to get home. I can say the maximum should be k1000 and for me, I would have some change left but the same amount would not be enough to take another person home.
233. I: Okay.
234. R4: I think it would be better to have a maximum amount. However, let us just set a minimum. In my case, I can be given k200 because I live near while the person next to me is given k5000 because of where they live.
235. R[Chorus]: [Laugh]
236. R4: Even though this one lives far, I would be very hurt [laughs].
237. R[Chorus]: [Laughs]
238. R4: I would not feel good about it. Therefore, I think it would be good to have a minimum amount. If it is k5000, everyone get that amount and for those who live far, it will go up from there.
239. R[multiple]: Yes!
240. I: Okay, what do others say? I saw you raised your hand.
241. R1: It is what he has said.
242. I: Okay, he said it should depend on where you live but it seems we all agree to have a minimum amount even if you live so close.
243. R [Chorus]: Yes, that is true!
244. I: You might even be tempted to change where you live and lie [laughs]
245. R [Multiple]: Yes!
246. R6: That can happen. You tell them your transport fare is k200 and they give you whilst your friend next to you I getting k5000. You can start having second thoughts and even say ‘I made a mistake’.
247. R?: You can easily say that ‘I made a mistake, that is where my sister lives not me’.
248. R6: If not that, what would happen is the people who come after us will only come to steal. When we cope other people, we might tell them that ‘when you get there, tell them that you live at such a place so that they give you a lot of money.’ People would start mentioning and asking for a lot of money, which will not even make sense. You can coax someone and tell them to say they spend k20, 000 for transport. Thinking of that does not even make sense because it does not cost that much to get to Blantyre for instance. However, because they were told that they would be given whatever amount they mention, they will come with such a mentality and you would have to argue with people. When we go out and we tell people, we need to tell them the truth and if it is adding, they should do what they can. However, it should not be so obvious that the person is lying. That is shameful.
249. I: Okay, what do others say? Or we agree?
250. R[Chorus]: We agree.
251. I: Okay, if not for the money, what else would we like to be given? Instead of the money, what would we like? Is my question clear?
252. R[Chorus]: Yes.
253. R1: Maybe there should also be a car available to drop you off afterwards.
254. I: Okay, you were also saying something.
255. R: [Silence]
256. R2: I think the alternative was already there. While I was in Salima, cars were coming to pick up young men, have them circumcised at Mvera and then drop them back home. I think that would also be a good method. However, for it to be successful, a lot of civic education needs to be done. In the communities, people should be told the truth so that you can pick them up and bring the here. It is a good method and it would work. However, the money refund is more attractive to an individual. It would really make someone interested and they would come.
257. I: Okay, you have said that with picking the people up, there needs to be a lot of civic education. Why is that?
258. R2: Because even there, the people will be surprised why they are being picked and dropped off. They might start saying that you have a hidden intention. So, there is need for civic education.
259. I: Okay, others?
260. R5: I think if there was a way of going into the communities and setting up places for circumcision on specific dates, going into their communities. I think that would be a good method unlike having them travel to get circumcised.
261. I: Okay, so the circumcision should happen in the communities?
262. R5: Yes!
263. I: Okay, what do others say?
264. R: [Silence]
265. I: Number 7, anything to say?
266. R7: No.
267. I: For the same refund, let us think of it in line with religion. How would it work out if were giving refunds after circumcision.
268. R4: For us Muslims, we would really be concerned and our leaders specifically. That is because it would completely reduce the number of people being circumcised in our culture. Most people would rush to be circumcised at the hospitals because as we have already said, money is a problem. So, there would be disappointment among our leaders because of the money. Few people would go to them compared to those who would go to the hospitals.
269. I: Okay, number 2.
270. R2: It is true, but that is because we are saying that ‘the people are being given money’. However, when we go back to what you said, the statement is ‘they are being refunded transport’. Those two statements are different. If someone is being refunded, it means they volunteered themselves, came to the hospital and because of that, they are being refunded so that they can get home. If someone is to think wrong, it would be because they have understood. However, my understanding is that they are refunding transport.
271. I: Okay, so the issue there is to stress that ‘we are not giving money, we are refunding transport’.
272. R2: Yes.
273. I: Okay, anything else to add? What do the other say?
274. R: [Silent]
275. I: Okay, we were looking at each strategy individually. However, what are your thoughts on using all these strategies at once? The people come, the get the intensive education. For those who chose to get circumcised, they are given an appointment date and then they get an SMS reminder. When they come for circumcision, they are given a refund; all these things are happening at once. What are your thoughts on using all the strategies at once? How would that be?
276. R3: I think that if all those were implemented at once as you have said, you get the message, you get circumcised and then you get a refund. That would help and people would benefit.
277. I: Okay, so you think it would help.
278. R3: Yes.
279. I: Okay.
280. R2: I think the government would benefit. That is because all the diseases that come about because of not being circumcised will be reduced by 60%, not so. They will be reduced by 60%. That means that even the health workers who were bothered by these diseases will relieved of the pressure. The government will benefit, medicine will start being available in cases where it was scarce. If many people are not circumcised, the medicine will continue being scarce because there are many people contracting diseases. Therefore, I think that it is very important and the government will benefit. The country would also develop because one aspect will be relieved.
281. I: Okay, did you have something to say?
282. R1: It is a very good thing because even after I have been circumcised, I will encourage my friends who have not been circumcised. I will encourage them so that they should also come to the hospital for circumcision after I explain the benefits to them.
283. I: Okay. At the back, number 10.
284. R10: I agree with what my friend in front has said and I do not have anything to add. I just agree with what he has said.
285. I: Okay, he says the government would benefit, but how do you think the individual coming for circumcision would benefit.
286. R6: The person who has had VMMC can benefit because he would not be at risk of diseases like cancer, the same for your wife, you would not put her at risk of cancer because you have had circumcision. Someone who has not been circumcised would not benefit because they are at risk of suffering from cancer.
287. I: Alright, are there any other views on combining these strategies?
288. R2: There is a benefit for the individual. When we are saying that circumcision helps prevent diseases, only a healthy person engages in development. If you are constantly sick, there is no way you do any developments, you cannot take care of your family. Individually, if you follow this method and you are circumcised, all the strategies we have discussed, if the person is healthy and they are preventing diseases, that person will develop his home. He will even develop his society and his country. It will also benefit the person. Thank you very much.
289. I: Okay, anything to add on combining all the strategies?
290. R: [Silence]
291. I: There is nothing else right?
292. R: [Silence]
293. I: My last question, and everyone will be required to respond to this one. We have talked of three strategies; intensive education, SMS tracing and reimbursement. Of these three, if you were to choose one strategy or a combination of two strategies which you feel would be very effective, which ones would you choose to combine? Everyone will respond to this one.
294. R?: If you could repeat the question.
295. I: Okay, what I said was that we have talked of three strategies, we all remember them, not so?
296. R [Chorus]: Yes.
297. I: If you were to choose one strategy out of the three or two strategies which you feel would be very effective if combined, which ones would you chose to combine or which one strategy would you prefer?
298. R4: For me, I would prefer this method of reimbursing transport and the strategy of intensive education. If these two were combined, they are very effective and would help, even without the SMS.
299. I: Okay, why do you think so; what makes you think they are really effective?
300. R4: I think they are very effective because these two have the capacity to ensure that the person does not fail to come for circumcision. Besides that, with these two, they would not be reluctant [discouraged] to come for circumcision.
301. I: Okay, others?
302. R3: I agree with what my friend number 4 has said.
303. I: In your own words, what would you say?
304. R4: I also think this is what would make men come here, get circumcised and go back home without any problems.
305. I: Okay, others?
306. R9: Umm, I agree with the points these two have said. For someone who wants to be circumcised, with intensive education and transport reimbursement, there is nothing that would make them fail to come. They will know that when I go to the clinic, I will find transport there and I will be taught.
307. I: Okay.
308. R2: I also agree with these strategies. That is because when we look at each strategy individually, they have the ability to make someone come for VMMC. When we look at the refund, on in own, it is very effective. When we pair it with intensive education, the person would come for circumcision knowing what they are coming to do. that is because they will be taught and well informed on what they will do. as such, I think these strategies are effective and very helpful. That is what I think.
309. I: Okay.
310. R1: I also agree with these methods. That is because… the issue is the same, they will make other people come for VMMC and when I go home after VMMC, I would also tell my friends so that they are not scared of it. All these stories that they use the foreskin as bait to catch fish are not true. The doctor told us that we are free to take that skin so long as we do not throw it along the way. Instead we should throw it in a pit latrine or burn it. Thank you.
311. I: Okay, what do others say? There are still several of us who have not spoken
312. R7: I also agree very much. That is because once you have been given transport, that is one way of encouraging the person to come. If they have been given the intensive education, it is also good because they will have enough information to tell their friends who have not been circumcised. As such, there is no limitation there because the person is given transport and they are taught. That will encourage the person to tell other people to come for VMMC. Thank you.
313. I: Okay, number 10.
314. R10: It is true that when he have been taught, we will be bold and we will remove fear. Apart from that, when the refund comes in, we will be encouraged to come here for VMMC knowing that we will not have transportation challenges when going back home.
315. I: Okay, about 5 left.
316. R5: I think it is a very good thing to have the education and refund. Those things would encourage the person to come to come for VMMC. When they hear of refund and they have been taught, they will see that it is a very good thing and this will go well.
317. I: Okay, what would you opt for?
318. R6: What?
319. I: Which strategies would you choose?
320. R6: I would choose the refund of the money I used to come to the clinic and to go back home.
321. I: Just that one?
322. R6: Yes.
323. I: Okay, number 8 is left. And 9 as well?
324. R9: I already gave my response on this one.
325. I: Okay.
326. R8: These two are the most powerful strategies and the most enticing as well. the refund and intensive education. That is because you would not go through with VMMC if you have not had good education. If you are going for VMMC, then you have been taught, meaning you know what you are going to do where you are going. You know the benefits and you know the disadvantages. When you come here and you are given transport money afterwards, that is important. Thank you very much.
327. I: Alright, thank you. Do we have any question or something we would like to add?
328. R4: There could be a question. While we were discussing, number 9 asked a question on whether you have an age range of those you allow to get circumcised. We discussed that question, but I do not know how you can assist us on that.
329. I: Okay, the question on age.
330. R4: Yes.
331. I: I have taken note of it. Any other question? I have kept your question alright?
332. R4: Yes
333. R7: I heard that if they do not cut the skin right, you can become barren. Is that true or not? I would like to understand that.
334. I: Okay, that is 7.
335. R8: After VMMC, how long can you travel on foot?
336. I: Soon after VMMC?
337. R8: Yes.
338. I: Okay, I have also taken note of that. Any other?
339. R5: When a person comes for the education, will they be refunded or the refund is only for when the person has come for VMMC?
340. I: Transport is being refunded only on the day of circumcision. The education will be given whilst the person is already at the clinic seeking another service. Transport is being refunded only on the day you come for circumcision. Another question?
341. R2: On the issue of education, did they chose to say the education will only be done here or they will also go out to teach people while in their communities? Like going in schools or churches or communities? Going in the communities and calling the chiefs. You can talk of educating people and you wait for them to come to the clinic but the people will not come here. Therefore, there is another way of going where the people are found, to teach them. For example, at some point, a car was going around, and spreading messages and teaching people. So, how will the teaching be done; will it be here at the hospital or you will also be going to other places?
342. I: At the moment, the plan was to have the education right here at the clinic. However, since you have said that going where the people are found would help, we have learnt something from that. Any other question?
343. R1: Mine is just a comment not a question. I was someone who listened to what people say about circumcision. With the counselling I have received today however, I have understood and I have made the decision to undergo VMMC.
344. I: Alright, any other comment?
345. R7: Mine is a question; for a married man who just got circumcised, how many days or how many weeks is he supposed to stay without have sex?
346. I: Okay, I have also taken note of that. Anything else?
347. R: [Silence]
348. I: That is all right?
349. R6: Yes, that is all.
350. I: [Chuckles] number 6 is tired.
351. R6: [Laughs]
352. I: Alright, this is also the end of what I had. I have taken note of all the questions on circumcision. However, everyone has their area of expertise, which is why I simply took note of the questions. I will ask my colleague who is very informed in this area and he will address your questions. I really appreciate your time today; this is the end of our discussion. Thank you.
353. R [Chorus]: Thank you.

THE END
