# Supplementary material for: Acceptability, feasibility and appropriateness of intensified health education, SMS/phone tracing and transport reimbursement for uptake of voluntary medical male circumcision in a sexually transmitted infections clinic in Malawi: A mixed methods study
Source: PLoS One. 2025 Jan 24;20(1):e0301952. doi: 10.1371/journal.pone.0301952 (PMC11760565; doi:10.1371/journal.pone.0301952)
Supplement: S1 Data — (ZIP) [file pone.0301952.s004.zip › Qualitative data/Endline FGD Transcripts/FGD4_Transcript.docx]

1. I: we want to see if this intervention to see if it can improve the desire for men to undergo medical circumcision and increase the number of men going for circumcision. All your responses are confidential and I already mentioned that your names will not appear on any forms so let us all remember not to mention our names, right?
2. R[chirus]: Yes
3. I: Please feel free to discuss your honest opinions. Remember, there is no right or wrong answers, just discuss openly right?
4. R[chorus]: Yes.
5. I: The issue we are talking about here is circumcision and so first of all, what have we heard about medical circumcision?
6. R[all]: [Silence]
7. I: Anything we have heard about it whether good or bad.
8. R1: I heard that medical circumcision helps to reduce transmittable diseases by about 60%. I heard that from here when I came to inquire more about circumcision.
9. I: Specifically, which diseases are those?
10. R1: Like AIDS.
11. I: Okay, that is what he heard. What have the rest of us heard?
12. R2: When I arrived and they explained about circumcision, after hearing what they were saying, I was encouraged because it is one way of reducing sexually transmitted diseases. There are a lot of sexually transmitted diseases and this reduces the risk. When I heard that, I was interested to do the same.
13. I: Okay, these two have talked about reducing the risk, how is this risk reduced?
14. R2: The risk is reduced because when we are having sex, we transmit diseases to each other through the wounds and so this would help so that we should not contract anything from those wounds.
15. I: Okay, what have others heard?
16. R4: It also promotes hygiene.
17. I: Okay, explain what you mean by hygiene.
18. R4: After having sex, you can wipe yourself yes but some liquid still remains. If you are not circumcised, that liquid stays on the inside of you whilst if you are circumcised, you can easily wipe it all away.
19. I: Okay, others?
20. R5: On the issue of circumcision, I know that when you are circumcised, they remove the foreskin which covers the tip of a man’s penis. This foreskin is the one that carries viruses which start several sexually transmitted diseases. When you remove that foreskin, you ae protected 60% like he has said. That means that after you remove the foreskin, you are protected for the most part from contracting sexually transmitted diseases.
21. I: Okay, is there anything else? We are all only talking of the benefits, what disadvantages have we heard of?
22. R?: Aaa, about its disadvantages, I think you can only them individually…
23. R5: The disadvantage is there. People are used, there are other people who are married and others who are not. For the married ones, it is often hard for them to stay without sex for a long time. With regards to circumcision, the disadvantage might be there because you are used to having sex with your wife. It might happen that your wife has the desire for you to have sex and they might end up finding another man to have sex with. That might be the disadvantage, considering the time it takes for your body to get back to normal.
24. I: Okay, if you have heard, how long does it take before someone is healed?
25. R5: After circumcision, they give you 6 weeks.
26. I: Okay, he has talked of the length of time it takes to heal and that might make the wife to look for other men right?
27. R[Chorus]: Yes.
28. I: What else have we heard concerning circumcision, it can be both the benefits and disadvantages.
29. R[All]: [Silence]
30. R5: There is another disadvantage and I do not know if this was solved. There are other people whose bodies develop lumps as they are healing. That is a challenge to other people because as a result of the circumcision, they might develop something on their body.
31. I: Okay, is there anything else?
32. R2: I think one thing that people do not understand when they talk of reducing the risk of contracting diseases, they do not understand what that means and they live carelessly thinking they cannot contract diseases. Some might even rape children because they have not understood how it works.
33. I: Okay, so because they do not understand, they do careless things thinking they cannot contract diseases.
34. R2: Yes, they think they cannot contract diseases.
35. I: Okay, and for you who are here, how would you feel about medical circumcision? I don’t mean that when you agree you will get circumcised right away, am just asking.
36. R2: For me, when I heard about circumcision, I thought it is a good thing and that it would help me. However, the other main thing is to follow what I have been told on how to care for the wound so that it heals in time.
37. I: What good thing did you see, that made you decide to go through with it?
38. R2: The main thing was hygiene.
39. I: Okay.
40. R5: Everyone on earth wishes to have a long life. When you are practicing hygiene, it helps you to really live long. Circumcision is therefore helping us to prevent sexually transmitted diseases.
41. I: Okay, and s for you, how do you feel about circumcision? You have talked about long life, but how do you feel about circumcision?
42. R5: I think circumcision is very important. that is because we don’t realize it, some people think that circumcision is only for the Yao group of people and that might be why some people do not come to the hospital for circumcision, because they are not Yao. However, I think medical circumcision is good regardless of your tribe.
43. I: Okay, number 3, let us hear your views. How would you feel about medical circumcision?
44. R3: It is a good thing to do medical circumcision because it can help with your life. If you are circumcised, it would be a good thing because you would have a long life.
45. I: Okay, how would it help you have a long life? How would circumcision help with that?
46. R3: Umm… I can say that after you are circumcised, you shouldn’t rush into sleeping with women because that would bring in problems which would make your life shorter.
47. I: Okay.
48. R2: I just have a plea to health personnel, circumcision is one way of promoting hygiene and so if you could enlighten people on that, it would be helpful. People are contracting diseases because of lack of hygiene. They think hygiene only applies to things like food and not our bodies and yet even our bodies need it. So, reaching out to people on the issue of circumcision is one way that hygiene would reach the people and they would be protected from the diseases. The diseases that are really scary right now are the sexually transmitted diseases.
49. I: Aright, is there anything we would like to add on how we would feel about circumcision: number 1?
50. R1: I am one of the people who have that desire and I will come here one of these days to have it done because I know its benefits.
51. I: Alright, if we had circumcision, would we tell our friends or relatives?
52. R[crosstalk]: Yes, we would.
53. I: Number 1.
54. R1: I would tell them the benefits of circumcision so that I should convince them to come.
55. I: Okay, you equally agreed.
56. R2: I agree and I would carry the information and tell other people.
57. I: So you would tell people that you have been circumcised?
58. R2: Very much! I would tell them that these are the benefits I have experienced with circumcision and so you should do the same.
59. I: If you experienced negative things, would you also tell them?
60. R2: If I experienced any negatives I would tell them, but that would not be the first thing I would tell them. Why is that? Because it is possible that the negative thing has only happened to me and they will not experienced it. I will tell them so that they can experience the issues like hygiene that are there.
61. I: So, you would start with the benefits and then later on the negatives maybe?
62. R2: Yes, maybe I would tell them the negatives later. I would start by telling them the positive things first. As number 5 said, it depends on the type of body the person has. Some can get circumcised and heal without a problem, they would even heal before the said number of days while it takes others longer to heal because of the different types of bodies. So, if I have experienced a problem which might be because of my body, it is possible that they will not experience the same so why would I start with the negatives? When telling someone something, you don’t start with the …. It’s the same thing if we were chatting as friends, there might be misunderstandings within the chatting. When that happens though, I can not only focus on the misunderstanding that is there, I also need to look at the benefits. The same way, I wouldn’t say that because I have experienced such a problem them another person should not go, no! it is better I tell them the benefits and if there are any disadvantages I would tell them that those only comes about because of not following the counsel given.
63. I: Okay, number 4, you also agreed.
64. R4: On the issue of telling others, we would not start by telling the negatives. We start breaking the issue down to the person slowly so that they understand what we are trying to say. We would start with the benefits of the thing and it should be up to them. its not like we wouldn’t tell them the bad things, but as my friend has said, the bad things would not happen to everyone.
65. I: Alright, you also agreed, why would you tell others about your circumcision?
66. R5: The reason why I would tell them that I went for circumcision is that… I will talk of the same issue of hygiene; so that their body hygiene will be improved. The second thing, I will talk of the bible. On the issue of tribes, the belief that people have that only the Yao should be circumcised. When you carefully read the bible, all the people in the old testament needed to be circumcised. People believe that Christians should not be circumcised but only the Muslims do. I would love it if this went to the religious leaders so that they tell their people that they should be circumcised.
67. I: Okay, you also agree to the same, is there anything to add?
68. R4: No.
69. I: [Chuckles] okay. We will start talking about the strategies that I mentioned in order to get your thoughts on how you think they would work.
70. R[Chorus]: Okay.
71. I: Firstly, we would like to conduct regular and detailed education on circumcision. While you were waiting someone came and taught right?
72. R[chorus]: Yes.
73. I: So, we are thinking of having such education everyday but detailed. How do you feel about receiving education on medical circumcision at this clinic?
74. R2: It is a very good strategy because it’s not like the people that are here today are the exact same people that will be there tomorrow. Different people come here. So, to ensure that these messages reach as far as possible, there is need for the education to be detailed and to be done everyday so that those who will come today or tomorrow can still hear it. If it is only given to us, it means those who come like tomorrow will not have it. So, it is important these are delivered daily.
75. I: Because different people come every day.
76. R2: If it were also possible to send the information via phones, it would also help.
77. I: Via phones like in what sense?
78. R2: It can be done through messages because I don’t think you would manage to call everyone.
79. I: Okay, we will come back to this issue. What do others think?
80. R4: The regular education is a good strategy. To add on that, I think if they would also go around in the communities spreading the information. It is true that different people from different areas come here. however, not all of us are able to tell our friends. But, if the medical personnel went to area 36 for instance, it means everyone in that aread would know. If it is only being done here at the clinic, a few people will know about it compared to those who would know if they went in the communities.
81. I: Okay, and so you feel going in the communities would work better than having the regular education at the clinic?
82. R4: Yes.
83. I: Okay.
84. R5: I think that the community one would work. The reason for that is… I will give us an example of the community I am coming from. A certain boy has died because of sexually transmitted diseases. What delayed him was that he started with taking traditional medicine up until he has lost his life. What that means is that he did not have the right counsel on how he would be helped effectively. Looking into it as well, the boy was not circumcised. The main cause of that could be because he was not circumcised. It would therefore be good if you were diligent in that because although there will be others who will be reluctant, when you come and you share the information, those who are reluctant will remain that way whilst those who will use the information will also use it.
85. I: Okay, number 1, you also had something to say.
86. R1: I also wanted to say that yes we come to the clinic and we hear these things. When we have heard these things, we can be carriers of the information and tell other people so that the information is spread. The Ministry of Health also have their microphones, they could also go around saying the same things and encouraging people.
87. I: Okay, what kind of information would you like to hear? Circumcision is circumcision right?
88. R[chorus]: Yes.
89. I: But which information do you think is important, information that the people need to hear about circumcision.
90. R2: If they were going around the communities, the first thing is that they should explain the benefits of circumcision, that it reduces the risk of contracting diseases. From what I have seen, the benefits are more than the risks and so the people should be told the benefits that they would have after circumcision. That would help.
91. I: Okay.
92. R2: You could do it the same way the Malawi Blood transfusion guys do it, they go out in the fields. If you have noticed, people rush to them when they come. If people are willing to give their blood to sav other peoples lives, they would do more to save their own lives. Often times, people do not come for circumcision because they have not heard and they have not been enlightened of the benefits of circumcision.
93. I: Okay, so we should talk of the benefits of circumcision.
94. R2: Yes, the benefits that are there.
95. I: Alright, number 3, what else should be included in this education?
96. R3: There is nothing I can explain.
97. I: Okay, what do others think? What information should it contain or which information do you feel is irrelevant?
98. R2: The irrelevant information is if you approach the people and tell them of the risks. It would be hard to protect a lot of people in that way.
99. I: [Chuckles] okay, so we should still not talk about the risks.
100. R2: No, if we do that then we will not protect them. when people hear of risks, even if there is 90% benefits and 10% risks, people will hold on to the risks, that is what they do.
101. I: Okay, number 2 says we should not talk of the risks, what do others say?
102. R2: We should tell them the benefits they stand to gain because these bodies are different. People face risks depending on their bodies. You might tell them of the risks and yet their bodies would not have had that risk. They will just end up being discouraged and they will not come here. it is better you tell them information that will help them.
103. R5: On that, it is obvious that we do not need to talk of any of the negatives. We only need to tell them the benefits. After they have understood the benefits, they ask the facilitator some questions and it is up to the facilitator to know how to respond to the questions so that the people sgould still get circumcised.
104. I: Okay, he agrees with number 2, what do the rest of us say?
105. R[chorus]: It is true, what they are saying is true.
106. I: Okay, so we should only talk of the risks after they are here.
107. R?: Yes, if you talk of the risks you will discourage the person from coming.
108. I: Okay, so we only tell the people the benefits and in the end, they come for the circumcision but they experience the bad things, what do you think such person will tell others in their community?
109. R2: It would depend on the person’s reasoning. When VMMC was starting, they had done their research and you can differentiate the risks that are there against the benefits that are there. Either way, it is one way of protecting people because currently, sexual relationshsips are normal, and so this would help protect our bodies. It would therefore depend on the person. As for me, if I came here and experienced some negative effects, I would simply find a way of resolving whatever I am facing but I would not be telling people about it. I would tell them what they would benefit from the circumcision and if they asked why I am going through what I am going through, I would tell them that my body simply did not react well or that I did not follow what I was told.
110. R5: Just to add, it is important for people to know the benefits. That is because when a person realizes the benefits, I don’t think they would decide not to go through with VMMC, if they realize the benefits.
111. I: Alright, is there anything to add?
112. R?: Nothing else.
113. I: Okay, the first one was on the education but before that one, he brought up the issue of sending messages. Please explain how these messages should be sent.
114. R2: Okay, I have come here today and I am talking with you. another way would be to get numbers so that we can keep in touch. For example, we have airtel and there are groupd like ESCOM and others who link up through them. they just say that if you need such information, you can call this number. They don’t give personal numbers, but it is a number that someone can call freely. I am thinking that if VMMC also had such a thing so that those who are interested can join. We also have Nkhoma Synod for example, people join and when the day comes, people receive messages from them and the same can be done here.
115. I: Okay, these messages are being sent to those who have come to the clinic and they have left their numbers.
116. R2: Yes.
117. I: Which means these are the same people that got the education?
118. R2: Yes. I am here today and I have been taught, when I got home people can learn from me. When I go home, I will forget some of the things we have talked about today and that will be like a reminder.
119. R?: Like an agent.
120. R2: Yes!
121. I: Okay, I see. I wanted to hear from you first because the second strategy that we are trying to do is to send messages as a reminder of your VMMC appointments at the clinic. What are your thoughts on this?
122. R2: It is important…
123. I: Let us start with number 4 first and then we will come to you…just hold on to the thought.
124. R4: Yes, it is really important because often times people have a lot of things to do which causes them to forget. However, if they remind you of the appointment day, it is a very goo method. That is because you will be able to remember such that if you had other plans, you can cancel them.
125. I: Okay, and so it would be a reminder?
126. R4: Yes.
127. I: Okay, number 3, what do you say? Receiving SMS reminders of your VMMC appointment date. What are your thoughts?
128. R3: [Silence]
129. I: What do you think?
130. R3: [Silence]
131. I: There are no thoughts?
132. R4: [silence]
133. I: Okay, number 1?
134. R5: It is important to be reminded because as he has said, sometimes you might be busy with other things but you would easily reschedule the other things. You can equally share that messages with other friends and if they want to come, they can come.
135. R2: That is a very important thing but it does not need to end just because the person has been circumcised. That is because for people who are coming here, I have learnt about it today and I know the benefits as well as how to be protect after circumcision. So, you could take us as agents and stay in touch with us even after we have gone back home. You need to stay in touch with us even while we are right in the communities because we would tell people that ‘I am circumcised and look at how am fairing’. That would help because Malawi as a country is poor and the messages cannot reach everyone through the phones but through us who have been taught about it. Even for those who use microphones and they go around the communities, those people do it in passing whilst we would be stationed right in the communities.
136. I: You have talked of others not being able to get the messages because of phones. What do you think can be done for such people?
137. R2: Okay, they came to the clinic, they have been taught but the message does not go through to them.
138. I: Yes, it could be because they do not have phones.
139. R2: I would say that the person should just… for someone who is really willing to g for VMMC, they should just… or you could give them something like a reminder right here at the clinic.
140. I: Okay, any other thoughts?
141. R5: I wanted to bring in another issue, but since this is being discussed it is fine.
142. I: Let’s hear what you wanted to bring in.
143. R5: What if it was instituted as a law that after every 4 or 5 days from the day the child is born, they should go for circumcion so that as the child grows, they are already circumcised. It should not be an option but every Malawian should go through that. In time to come, this will not be an issue anymore. If it were passed as a law.
144. I: Okay, what do others think?
145. R2: It is a good thing but what happens is that when people bring in such issues, they are always in conflict with religion. I think the first thing that needs to be done is to approach the religious leaders because this issue is also a problem to them…
146. I: So you are agreeing with what number 5 said?
147. R2: Yes, I agree just that we would first nee to approach religious leaders.
148. I: Okay, but on the issue of SMS reminders, how do you think the religious leaders would react? This question is going to everyone; how do you think people would react to this based on the different cultures or religions? Someone other than number 2 should start.
149. R2: [chuckles] alright.
150. R4: I think it is a good things. Let us go back a little bit to when he said that those of us who have come here can spread the information when we go back home, I think that is a good thing. Even the bible says that you cannot teach the bible if you do not know the bible. Since we have come here and we have been taught, we can take the things that we have learnt and teach others. On the issue of the SMSs, it is a good thing because there are other people who live far from the clinic. On this one then, I think you would need to talk to the network providers so that they send the messages to everyone and not choose who receives them or not. There are people who do not come to the hospital often but they would go through VMMC if only they heard about it.
151. I: Okay, so the SMS should not only be sent to those who have been given appointments but to everyone?
152. R4: Yes, I think they should be sent to everyone.
153. I: Okay, so let’s think of people in the communities where we live, if someone received a message talking about circumcision, how would they feel?
154. R4: It is not something new. We receive different messages on the phones all the time and this is very important because first of all, it is promoting hygiene and reducing your risk of contracting HIV. Although it is not protecting you 100%, 60% is a lot more than the 40% that is left.
155. I: Okay, and so you are saying there is no problem.
156. R4: There is no problem.
157. I: Okay, number 5, it seemed you had something to say.
158. R5: Yes, as he has said, when sending these messages each network has to be used in sending such information. I believe there is a time when that happened, they were sending SMSs using the airtel network. These were reaching everyone but when someone receives the message and sees that it is about circumcision, they were just ignoring it. I think the messages should still be sent to the people and it should be up to them to go for VMMC or not. I want to also talk about the religious leaders. Those are very important people to use. you will draft your references and give it to them and when they meet on their forums, they agree on the things to implement after they have understood it. They would then take those references and present them to the congregation. If they have understood it, they will present it to them and later on that can be presented in parliament where they would agree because it has started from the church.
159. I: Okay, you said that these messages were sent previously but that people would ignore them when they see it is something to do with circumcision.
160. R5: Yes.
161. I: Why do you think they were reacting that way?
162. R5: First because of culture and the second reason is the religion.
163. I: On culture is it the same thing of it being thought of as something the Yao do?
164. R5: Yes, something for the Yao’s only.
165. I: Okay, and on religion?
166. R5: Most people feel that only the Muslims do it.
167. I: Okay, and how do you think they would react if they received these messages at present?
168. R5: They would react well.
169. I: Why do you say that?
170. R5: Umm, there were only a few churches in the past but there are a lot more now and when you compare it with the past, people rush to the religious leaders whenever they experience anything negative. That leader has a task of looking out for the wellbeing of his people. If the people are not cared for then he is not doing right. When the people go to him, he guides them and he can include the issues of circumcision in that.
171. I: Okay, is there anything we would like to add on the issue of circumcision?
172. R2: Yes, in short, I can explain that as health personnel, there are a lot of messages that you send out. the same way you send messages on other issues, you should do the same with this one regardless of how you think they might react. But for these messages to attract a good reaction, it should contain the benefits they stand to gain from circumcision because people will easily rush to it.
173. I: Okay, number 3 is looking very tired.
174. R3: Ehh, in all honesty, I am very tired.
175. I: Okay, so what do we do?
176. R3: I can leave?
177. I: If you want to, yes you can.
178. R3: Okay, because I am very sleepy and tired so …. [walks out of the discussion]
179. I: Another strategy is to reimburse money after being circumcised to help with your transportation costs. How would you feel about receiving money after being circumcised?
180. R4: They thought very wisely of that.
181. I: Why do you say that?
182. R4: Because after circumcision you are in pain and so the money will help you get home safely. Apart from that, it will also help who live very far to come for VMMC knowing that transport will be provided. That is because some people are reluctant to say ‘I should go to the clinic and back as far as it is, it is better I just stay home’. So, they thought really well on this one especially for those who live far from the hospital.
183. I: Okay, number 1, any thoughts?
184. R1: it’s the same thing he has said that that was a wise thought. As he has said, you are in pain afterwards and you might have trouble walking such that if you force yourself, you might just make the wound worse. If you have transport however, there would be no problem and you would also encourage others to say they will give you back the transport.
185. I: Okay, so it would also encourage others.
186. R1: Yes.
187. I: Okay, do we agree?
188. R2&4: Yes.
189. R5: I think that apart from the transport, people walk around in the communities and they circumcise. So, we can talk about transport reimbursement but if there was something in place which they would receive after the VMMC.
190. I: What should the something be?
191. R5: Ehh!
192. R2: In line with what he is saying, most people would not make a decision to go through with VMMC if they have not been taught. The best way would be to spread the information so that they are first taught. When they are being taught, you can tell them to say ‘whether you get circumcised or not, there is transport’ so the issue of transport should be in two ways, first the education then the transport because people would only be interested after they have learnt and after the interest is already there, the transport should come in. without the education, their minds will go back to the misconceptions.
193. I: Okay, going back to the issue of money, how much should it be or what can be given instead of the money?
194. R2: I think if the money remained, it would be good because that is one thing that people would run to after they have been taught…
195. I: And so I want the amount of money which you feel could be suitable.
196. R2: Ehh, help me out there.
197. R?: [Laughs]
198. I: The question is open to everyone.
199. R?: I think it will be hard to come up with a figure because for someone who lives in Mtandire, it would not make sense to give them mk5000 but it would make sense to give that amount to someone coming from Kasungu. So, what I think is that you should ask the person where they live and give them according to where they live.
200. I: Okay, lets imagine people telling each other that ‘when you go to the clinic, they ask where you live and then they give you transport money’, don’t you think everyone will say they live in Mulanje? Just thinking about it.
201. R[multiple]: [laughs]
202. R2: Another way would be that when the person is coming, they should bring their old health books.
203. I: Okay, but most people buy books at the gate.
204. R2: Yes, but I think using the old books would be better. Another way would be that if the person says that they are coming from Kasungu, there is a hospital there that does VMMC and so they would not come here. the same thing with places like Dowa, meaning those who would come here live within Lilongwe. Maybe you could take k2000 out of the transport and give it to someone who has taken part in the education unlike asking them where they live because yes, people will always mention areas that are far. I recall a time when I went to donate blood, and they were equally reimbursing transport. I was at Mponela at the time and I had been there for 3 days. They were asking where we live and others were mentioning Dowa or Kasungu but I told them that I live in Kasungu but I was already in Mponela for other reasons and not for the blood donation. When I told them that, they said I had done well and gave me k1000. They appreciated that I was honest because others would have insisted on being reimbursed transport for Kasungu. So, it would depend on the reasoning of the person.
205. I: Okay, are there any other thoughts?
206. R?: On the issue of transport, the main thing is that for those who are already in Lilongwe, it is a city and when we go to Area 25, those areas already have clinics where they can be assisted, the same with Likuni.
207. I: Alright, you mentioned that people might rush to the clinic when they hear there is money involved. However, let us think of our communities, if they heard that there is reimbursement after circumcision, how would they react?
208. R2: Umm, mainly…
209. I: Let us start with number 5.
210. R5: People are different and the way they reason is also different. With that, people might react different. Some may say it is satanic, but, the main thing is the education.
211. I: So they would say it is satanic.
212. R5: Yes, there would be others who would say that but in the midst of that, some people will still come.
213. I: Okay, so some will talk but others will come.
214. R5: Yes.
215. I: Number 2, you had something to say.
216. R2: I will just comment on what he has said. When things are happening, there are people who say it is satanic but there are still people who are interested in the thing. It all depends on the reasoning of the person and so some people miss out on things that would help them because of reasoning like that. What is needed is for the education so that people know about it.
217. I: Okay, we previously talked of the Muslims being thought of as those who go through circumcision but not limiting it to the Muslims only, how do you think the different religions or culture would react if they knew that transport is being reimbursed at the clinic?
218. R2: That is why I said that the religious leaders should know. Approaching the leaders and explain to them. its not like this money is being given as payment for circumcision, but the aim is so that the person can travel easily to their home. If someone is from Area 25, they cannot walk back and so this strategy would help those people with transportation…
219. I: And so we need to approach the religious leaders and explain to them.
220. R2: Yes, and that is the easiest approach. Even when you are talking about issues to do with the antenatal, you still approach the chiefs first and tell them what you have come with as well as its benefits so they understand, the same thing with this one.
221. I: Okay, I understand. We were talking of these strategies one at a time, being implemented separately, but what are your thoughts about using education, SMS reminders and receiving money in combination
222. R2: Okay, these things are happening because the person has volunteered to come here, they have been taught and they have decided to undergo VMMC. When I leave the clinic, I can meet other people and others would discourage me or encourage me. When you send the SMS however, I would be encouraged and I would still come for VMMC.
223. I: Okay, if you were to choose one strategy or a combination of strategies which you feel would help increase the uptake of VMMC, which one or which ones would you choose? Everyone will respond to this one, let us start with number1.
224. R2: Sorry, let me just cut it, I think it would be good if you repeated the strategies we have discussed.
225. I: Okay, I hear you. the strategies are Reimbursement for transport, SMS reminders and Intensified education. If you were to choose one or a combination of two which would work better, which one or which ones would you come?
226. R1: I would choose all of them.
227. I: All the three?
228. R1: Yes, because if they have been taught, they need to come and get circumcised and you would reimburse them.
229. I: Okay, you have left the SMSs out.
230. R1: For the SMSs, it is good to remind them because if there are things that might have stopped them from coming, when they are reminded and they know that they will be reimbursed, they will come.
231. I: Okay.
232. R4: I also opt for all of them. that is because when they receive the reminder, the will want to learn about it and then they will opt for circumcision. When they are circumcised, they will be reimbursed and they will go back home. So, all those strategies need to go together.
233. I: Okay.
234. R2: I also agree with what they have said.
235. I: [Chuckles] you are all copying each other.
236. R2: [chuckles] no, let us explain the reasons. A person needs to be informed for them to decide. The SMS reminds the person of the decision they have made because you cannot force anyone to get circumcised. The transport is very important so that the person easily travels.
237. I: Okay, so you are choosing all of them.
238. R2: Yes, [chuckles]
239. I: Number 5.
240. R5: All those strategies need to go hand in hand. First, there needs to be funding and after that, we need the education.
241. I: What do you mean by funding?
242. R5: The money for transport should be there, then the person should be told and then they should get circumcised.
243. I: Okay, which means you are not including the messages right.
244. R5: No, the messages should be there because they will reach out to everyone.
245. I: There is no strategy which you feel would work on its own, they have to be all three?
246. R1: Yes.
247. I: Number 1 says yes.
248. R: The two strategies, the transport reimbursement and the education, not including the messages because as much as we are talking about it, some people don’t have phones. It can happen that the day the message is sent is the day your phone is off and it is hard to borrow and use someone’s phone. I think the two things are very important; the education and the transport.
249. I: Okay, number 4.
250. R4: The strategy that would help is the reimbursement. Of course, the others are helpful but as my friends have said, some do not have a phone. they did not come here and so they have not been taught either. However, if I am circumcised, I can tell another person that I have been circumcised and such happened. From that, they would individually be interested in it even if they have not been taught.
251. I: Alright, is there anything we would like to add or share with me on the issue of circumcision.
252. R2: What I can add on what we have learnt here is just to encourage the health personnel to have time to reach out to people and tell them of the benefits of circumcision. Most of us are here and we have not been circumcised because we did not know. So, the same information which we have accessed needs be taught to other people in other areas so that they realize it. They need to walk around so that other people an also have it.
253. I: Okay, is there anything else?
254. R5: This issue of circumcision is big and everyone needs to know about it. Like I said, if a law was passed so that in future, as every child is growing, they will have already been circumcised and that would help us. If we only focus on us who are here, what of the generation to come? It is therefore good to fix these things now.
255. I: Alright, there is nothing else?
256. R2: Yeah, I think that is all. There was a time I was blessed and I composed a song about circumcision and you should listen to it one day. I even performed it just that it was back in the village [chuckles]
257. I: [chuckles] well, thank you very much for the time we have had today. This is the end of the questions that I had.
258. R[chorus]: Thank you.

THE END
