# Supplementary material for: Acceptability, feasibility and appropriateness of intensified health education, SMS/phone tracing and transport reimbursement for uptake of voluntary medical male circumcision in a sexually transmitted infections clinic in Malawi: A mixed methods study
Source: PLoS One. 2025 Jan 24;20(1):e0301952. doi: 10.1371/journal.pone.0301952 (PMC11760565; doi:10.1371/journal.pone.0301952)
Supplement: S1 Data — (ZIP) [file pone.0301952.s004.zip › Qualitative data/Endline FGD Transcripts/FGD3_Transcript.docx]

1. I: Alright, first of all, tell me what you have heard concerning medical circumcision.
2. R3: Concerning medical circumcision, most times we hear about the circumcision but what happens at times is that people sometimes say that when the circumcision happens, the hospital people benefit more out of it than us. they say that it is some kind of business that the government is running.
3. I: Okay, do they ever say how the government is benefiting?
4. R3: At times they say that after the circumcision is done, no one knows where they throw the foreskin, only the medical people know. Even if you tell them to put the foreskin in a gab and give it to you, that is still not possible meaning that is their benefit.
5. I: Okay, so they use the foreskin for business?
6. R3: Yes.
7. I: Okay, what have others heard concerning medical circumcision?
8. R1: I just want to agree with what my friend has said and to add what I know. Most people, I think that is why you said that the people who come fr circumcision are less than the actual population, the reason could be what he has said. Most people, especially those from rural areas have wrong mentalities or ways of understanding where they say that when you go through medical circumcision, they sell the foreskin to western countries and others say that the foreskin is used to catch sharks. I think such issues are the ones that discourage people. However, even I was that kind of person who believed what was being said. After getting counselling several times, I am now aware to say these things are good. Sometimes we took it as part of a specific culture but these things help us reduce the risk of contracting sexually transmitted diseases, cervical cancer and the like. Currently, I understand what male circumcision entails so that we should prevent sexually transmitted diseases and to prevent cervical cancer in women.
9. I: Okay, he heard that the foreskin is used for shark fishing, what else have others heard concerning medical circumcision?
10. R2: The main things have been said by him, that is also the point I wanted to talk about.
11. I: Okay, apart from people not knowing what happens to the foreskin after circumcision, what else have we heard concerning medical circumcision? What do people say about medical circumcision or the benefits of it.
12. R3: When we listen to the counselling concerning medical circumcision which medical personnel give, they say that medical circumcision is good for men. The first reason is that it protects you from contracting diseases. It does not mean that you cannot contract diseases, but rather, you have some protection. What happens is that when you have sex, you do not have anything left over after that because you no longer have the skin that covered the penis. In that way, you protect yourself and you also protect the woman you are sleeping with from cervical cancer.
13. I: Okay, what other benefits have we heard of? the benefits or disadvantages of medical circumcision. This line going this side, what have we heard?
14. R4: Mine will the same, the benefits are what he has said that you are protected from diseases. So, I will ask to say if you have been circumcised, can you really not contract any diseases?
15. I: Anyone who can respond to that.
16. R1: Male circumcision helps reduce your risk of contracting diseases and not that you cannot contract disease, the chances of you contracting diseases are just low. If you are not circumcised, there are some viruses which might cause diseases when you sleep with a woman. When you are circumcised, there is a big chance that you would not transmit anything to the woman because the viruses hide in the foreskin. When you remove the foreskin, you cannot transmit anything to her.
17. I: Is that clear?
18. R4: Yes, it is clear.
19. I: Okay, what were your thoughts initially, that you would still contract diseases after circumcision or not?
20. R4: There was also a risk that even if I am circumcised, I would still get diseases but I thought that was not true.
21. I: What was not true?
22. R4: That you would contract diseases after being circumcised.
23. I: Okay, let’s hear from this side, what have you heard about medical circumcision?
24. R6: We wanted to know the truth because we only hear rumors from people that after we are circumcised, if someone had viruses for instance, just like he said that viruses hide in the foreskin and that after circumcision, the viruses go away. So, we wanted to know the real truth on whether the viruses really go away or not.
25. I: Okay, and it is still not clear from his response? Because your question is similar to the one number 4 had.
26. R6: Yes, now I am sure.
27. I: Meaning people say the viruses go away after circumcision?
28. R6: Yes, and that you would not contract any diseases because you are circumcised.
29. I: Okay, what else do they say? I want to hear everything.
30. R5: Is it true that someone who has HIV cannot be circumcised? Why is that?
31. I: That is what you heard?
32. R5: Yes, I have heard people say that.
33. I: What reasons do they give?
34. R5: That is what I wanted to know because when I came here for circumcision, I came and I entered the room together with some friends I had come with. My friend went in first and he tested HIV positive and they told him that he could not get circumcised. He also had some sores. From that, I was discouraged and we just when back home.
35. I: The person had HIV or the sores?
36. R5: He has HIV and the sores.
37. I: Okay, and the reason why they could not circumcise him was because of the sores or because he had HIV?
38. R5: He said it was because of the HIV and not the sores.
39. I: Okay, I think we can keep that question and ask the guy who was teaching us to explain further. But if you think about it, why do you think they would not allow someone who is HIV positive to get circumcised?
40. R5: I think it is because the person already contracted HIV. The ones that get circumcised do it so that they should not contract the diseases. This one is already damaged and so there is no need to get circumcised anymore.
41. I: Okay.
42. R6: From what he has said, if someone has sores and does not have HIV, is it possible to circumcise the person when they have sores?
43. I: What do you think? Would it be possible or not?
44. R6: No.
45. I: It would not be possible?
46. R6: I think they would refuse to circumcise him because of the sores.
47. I: Why exactly would they refuse to circumcise him?
48. R6: Because he has the sores. They would think that he has another disease like HIV. That is why they would not circumcise him.
49. I: Okay we will equally ask the other guy that question. However, we are still on the same question. Is there anything else we have heard about medical circumcision?
50. R5: Yes, I will still ask because I have heard from several people that when you are circumcised, they use the foreskin for medical uses. When you tell them to give you the skin, they do not give it to you.
51. I: Okay, that is something you have heard.
52. R5: Yes.
53. I: Okay, and so it is similar to what he was saying and you have added the issue of using it for medicine. anything else we have heard?
54. R3: After hearing a lot of things that people say…
55. I: What do people say?
56. R3: As I explained about using the foreskin for business and the like… I made the decision to let my first born child get circumcised first so that as he grows, he should tell me what he is feeling. He is in form 2 now and so I ask him to say I got you circumcised as a young boy, what benefit do you see of being circumcised? He does not explain clearly because I am his father but he says that ‘when I am at school and I ask my friends, they tell me that the benefits of circumcision are that you can sleep with several women and you don’t feel any pain because the foreskin is not there. However, if you are not circumcised, you tire easily and you feel pain even after only sleeping with the woman once or twice, you cannot sleep with another woman again. I was not convinced with his response because I wanted him to tell me some tangible benefits. So, this could be my opportunity to ask from you because the rest is just hearsay. You have learnt of things however and you know the benefits as well as the risks so I would like to turn the question back to you to say what are the benefits and what are the risks because everything has its advantages and disadvantages.
57. I: Okay, I have hears that and I will equally keep this question because as I said, there is someone who is experienced in this field and it is the man who we were with earlier. Didn’t he teach today?
58. R?: He started but then he was interrupted and did not continue.
59. I: Okay, and so we will ask him the questions that we have and he will explain. We have talked of several things and he said that he came for circumcision but changed his mind after his friend was turned back. But, how do you feel about medical circumcision at the moment? I am not saying you will get circumcised right away, I just want to hear what you feel about medical circumcision.
60. R5: in my case, am just waiting for schools to close and I will come for VMMC.
61. I: Okay, why?
62. R5: Because the women that I meet… I heard a certain guy ridiculed by women to say ‘you are not circumcised, you are dirty! Your foreskin keeps things and you will infect us!’ and so I decided to get circumcised.
63. I: Okay, so that the same does not happen to you?
64. R5: Yes.
65. I: Okay, what do others say?
66. R?: I agree with what he has said because I experienced that as well. some women say that a circumcised man is different from a circumcised man.
67. I: What is the difference?
68. R?: The difference is that the uncircumcised man cannot last long while having sex with a woman and the pleasure is different between the two.
69. I: Okay, and so that the reason why you would want to get circumcised.
70. R?: Yes, so that they do not feel like ‘that man is not good enough in bed’ or ‘that man is Chewa and he knows nothing’. I don’t want them to say that.
71. I: Okay, how do others feel about circumcision? Let us start with number 1 then we will go to number 3.
72. R1: From the counselling that I got, I realized that circumcision is good because there are some things that we are protected from. If you are not circumcised for instance, there are certain things that you feel. because you have the foreskin, you can feel itchy and you keep things in the foreskin like grain. When you are circumcised however, that grain is no longer there. I also heard that when you feel itchy, sometimes it is because of a certain disease which we do not know about. When we are circumcised however, you prevent some diseases. So, I would get circumcised so that I prevent the sexually transmitted diseases.
73. I: Okay, number 3 you also had something to say.
74. R3: Yes, I also agree with what my friend has said. First of all, circumcision seems to be very good because it keeps us hygiene. Am not saying that you do not contract diseases when you are circumcised, but, you prevent some diseases. If you were to look, most uncircumcised men are contracting diseases easily, one moment you test positive for Syphilis and the next you test positive for Gonorrhea. That means it is possible that circumcision has benefits where first, you are safeguarding your own life and secondly you are keeping yourself clean. You are the only one responsible for your life and so you are protecting yourself by doing this.
75. R1: Just toad, one other thing that has made me want to do it is that since I am married, I will also protect my wife from cervical cancer in the process. To be honest, cancer is a very bad disease and I heard that when you are circumcised, you protect the woman you are sleeping with from cervical cancer. So, I felt it is a good thing to protect my wife because I am married and to make sure I live a healthy life as well.
76. I: Okay, are there any other thoughts on circumcision? Number 4 and number 6, any thoughts?
77. R: [Silence]
78. I: Alternatively, what have we heard about the pain after circumcision?
79. R6: I heard that after circumcision, it takes up to a week… it varies from person to person but I heard that after circumcision, you are in pain for over a week. Because of that, most of us are reluctant because after circumcision we would need to stay home and yet most of us are used to going to work or even school. That is something hard for us when it comes to VMMC.
80. I: Okay, because you would have to stay home for a week after circumcision instead of going to work.
81. R6: Yes.
82. I: Okay, what do others say? There is a lot we can talk about like would we tell our friends or relatives after we did VMMC?
83. R[Multiple]: Yes.
84. I: Okay, 1 and 2, why would you tell them?
85. R2: You would tell them because what made you go for VMMC in the first place are benefits that you were told. Equally, after you have been circumcised, you need to tell them the benefits that you found because we need to share these benefits.
86. I: Okay, so you would tell them because you equally learnt of the benefits.
87. R2: Yes, because of the benefits. With regards to what he is saying that people stay home for a week or 2 weeks for others, that could be true but it could also not be true. That is because it depends on how you are caring for yourself after circumcision. If you are not following what they told you and you are not bathing, instead of the wound healing, it takes longer. With the current state of livelihood, for you to stay a week or more at home, everything stops. If you are married, food will be a problem and if you look after children, they will equally suffer. As a result, some start to think that ‘yes, I have heard of the benefits and I will go to the clinic for VMMC but I carry bags in town to earn a living. If I go for circumcision, how will I survive for the days that I have to stay home?’ as a result, instead of doing that which will benefit you, you are forced to keep going to work so that you can assist your family since you have a responsibility at home.
88. I: Okay, any other thoughts there?
89. R?: The issue is what he has explained. There is not a day that we don’t come to town because we need to support our lives. So, what he is saying is true.
90. I: Okay, I understand.
91. R6: When we are home after being circumcised, It is very hard because you think of what you will eat if you are just staying home. A lot of things will suffer as well because you ae not going to work after circumcision. You will have done yourself some good but to other people it will not seem so good. They will look at the good thing you have done as something bad because you are just staying and not doing anything at home.
92. I: Alright.
93. R1: I just want to comment on what he has said. I think the one thing that makes people not to come for circumcision is lack of proper information. Like he is saying, the things that happen after circumcision, people say that the pain one goes through after circumcision is unbearable. Some people say that you lose a lot of blood after circumcision and that discourages a lot of people. I think that all this hearsay, since the actual information is not reaching them, especially the people in the rural areas. Not many of the people who live in the rural areas would come here, because they have a wrong mentality. They say ‘when you are circumcised the pain is unbearable and you lose a lot of blood’. People who live in the rural areas are the kind not to change their mind when they are settled on a particular thing. So, I think that is one thing that discourages people from circumcision.
94. I: Okay, yes, you also have something to say?
95. R4: Just to add on what he has said, at times, people do not come here for VMMC because of having to stay home for two weeks after circumcision. So, looking at the time you have to lose when staying home, it is better to use that time to find food for the family to eat. In my case, that is really true. It is really true. The second thing is that I have gotten some points on the benefits of circumcision. I have been taught and so I will be courageous and take part in VMMC.
96. I: Okay, he said that people do not have information because all they know is hearsay, they did not hear it directly from the hospital. One strategy that we are planning to try out is to conduct regular and more detailed education on circumcision. How do you feel about receiving education on medical circumcision at this clinic?
97. R2: I think that the education would be good so that people know the benefits of circumcision. However, people from the rural areas have a few questions in their mind. They ask themselves the questions and respond to it themselves as well. to give an example, there was a car which was furring children from Likuni and Chinsapo to Airwing. People would ask themselves to say ‘they have taken our children and they have used a very expensive car, could cost millions. However, the car is not running on water, it needs fuel. The driver who is driving is also just employed, he needs to get paid monthly. So, what are they benefiting from all this that they are doing? Such is question that if someone asked you in public, in front of other people, you would not easily respond to, you would stammer. So, when the education is being provided, the one teaching should know that these people have such questions and even they don’t let the questions out, they have them in their heart.
98. I: Okay, so do you think this education should be there as in would it change anything or not?
99. R2: It was depends on the persons understanding. Let us take the issue of the COVID vaccine. There are some people who refused to et a dose, they did not even get the first does. When you ask some, they tell you that I am not done with child bearing and some say that I just married my wife, so we do not have any children. Even the wife will strongly deny it, it all depends on their understanding. The same with this, it all depends on how the people will understand it individually and how they will use the information.
100. I: Okay, what do others feel about receiving education on medical circumcision at this clinic?
101. R1: I think that for the education to be given at this clinic…I think it will take too log for it to reach the population you would prefer. If say Malawi has a population of 2 million and you want at least 1 million, if the education is only being given at this clinic, it will take you time. the number of people that come to this clinic is less than the number of people who do not come. So, if the education was being given elsewhere as well… I feel that if you travelled out of the clinic it would really help so that those who are scared to come to the clinic will hear the message directly from you.
102. I: Okay, so what you are saying is that if only done at this clinic, it will take us a long time but if we go out in the communities it will help.
103. R1: Yes, very much.
104. I: Okay, what do others think about this education being given at this clinic. Number 5, let us hear from you.
105. R5: [Silence]
106. I: It looks like you were not here.
107. R?: On that one, you need to go in the communities because some people are scared to come here for VMMC and then go back home on foot or walk to the clinic and back home on foot after VMMC. It would be better if you were picked from home, circumcised and then dropped off back home.
108. I: Okay, the going out he is talking about is to teach about VMMC and not to do the actual VMMC when we travel. Right?
109. R?: Yes.
110. I: So what you are saying is that people should be picked from the community to the clinic because of the transport issues?
111. R?: Yes.
112. I: Okay, and so let’s say the people have been picked and they are now at the clinic, do you think the education on medical circumcision would help or not?
113. R?: [Silence]
114. I: Anyone can respond to this question.
115. R1: I agree with what he has said because if we are only being given the information when we come to the clinic, then it will just be hospital education because people will only come to the clinic if they are sick. The best way, which would really be helpful is to reach out… you can travel from here to Mbingwa village and plan for a soccer match between teams, teams that are famous and has players which the people in the rural area have never seen. People will rush to see the players and to watch the football. When the people come, before the match starts, the first thing is to give education. The same way others bring in dramas, after the drama is finished, they bring in some sort of counselling for the people. Their main aim for coming is not for the education, they have come for the football.
116. I: Okay, but whether we are in the communities or we are here at the clinic, what information about circumcision should we include?
117. R: [Silence]
118. I: Just your thoughts, we keep talking of information but what should we really explain or talk about concerning the circumcision.
119. R: [Silence]
120. I: 6 says no. what about number 2; what information do you think is useful?
121. R2: Encouraging others to come for circumcision.
122. I: SO how do we encourage them? what do we say?
123. R2: You should explain to them in detail on what happens. you should tell them exactly what you have told when teaching us today. What was said is right and so you should tell them the same and if they are willing, they will come.
124. I: Okay, so I want you to mention the things which we should tell them in detail. Give me examples.
125. R2: [Silence]
126. R1: The benefits of circumcision.
127. I: Okay, number 3?
128. R3: I wanted to say the same; the benefits of circumcision. When explaining the benefits… for instance, you cannot tell someone that South Africa is beautiful and yet you have never been there. When you are telling them that Cape Town is beautiful and it has some nice tall buildings, it should mean that you have been there and you have seen them.
129. I: Okay, meaning those who have been circumcised are the ones who should talk about it?
130. R3: Yes, they should do the explaining.
131. R1: How would one talk about circumcision if they have not done it.
132. I: Would not work?
133. R1: No, the issue is what I said earlier that you need to give the people the right kind of information because most people are blind and that is why they are reluctant. However, reach out to them, there are a lot of ways through which you can reach out. as I said, if you are only giving the education here at the clinic, not many people will hear it. For example, there were a lot of us there but out of all the people that were there, only the few of us are here. why do you think that is the case? It is because we know the benefits, we were told the benefits. So, if you reach out to the rest as well, the numbers will increase.
134. I: Okay, apart from the benefits, what else should the education include? Anyone can respond, maybe we can focus on this side, you have been very quiet. What else should we include in the education apart from the benefits or what should we not tell them concerning circumcision?
135. R? You should not tell them the things that are wrong.
136. I: Okay, what do you mean?
137. R?: What he said earlier that people lose a lot of blood and things like that. People will be scared to come here if they hear that.
138. I: Okay, so we should not tell them the negative things concerning circumcision, we should only tell them the benefits?
139. R?: Yes.
140. I: Okay, you also had something to say number 3?
141. R3: Yes, I wanted to also talk a little about what he has said. There are always advantages and disadvantages to things and the one who would know about these are the ones who are circumcised. So, these people or us who want to be circumcised need to… because the disadvantages are less than the advantages that are there which makes me think that the circumcision is good because first, you protect yourself, if you are married, you protect your family like your wife and you also keep yourself clean. It seems the benefits are a lot. For the disadvantages, people explain that when you are circumcise, the wound takes time to heal and it is uncomfortable or that you lose blood. However, even if you lose the blood, you still heal after some days. There is no one who dies because of circumcision.
142. I: Okay, so do you think we should include the disadvantages or not?
143. R3: I think that we should not tell them. if they want to know the disadvantages, they should use the same way I used for me to know the disadvantages.
144. I: Meaning they should know the disadvantages after circumcision?
145. R3: Yes, and not telling them before they come of its disadvantages.
146. R1: To add on what he is saying, when we are coming to the hospital, we do not know the medicine that you will give us before hand. I might be sick and not know if I will need an injection or the type of medicine I will be given. I accept the injection if they say I need to be injected because I want to get better. It’s the same with these people, after you have given them enough information, they cannot worry about the pain because that is an obvious thing. I will keep going in circles but the main point is that you should give them adequate information.
147. I: Without telling them the disadvantages.
148. R1: No, do not tell them because that will simply agree with what they already hear from people and they would decide not to come.
149. I: Okay, I will talk about two other strategies that we are thinking of trying out. the first is receiving SMS reminders. Say the person comes to the clinic and they want to get circumcised, they are given an appointment date and they go home. While home, before their appointment day, they should receive an SMS reminding them of their appointment. How do you feel about this strategy of receiving an SMS from the clinic reminding you of your VMMC appointment? How would you feel?
150. R1: I would like to hear the other one first.
151. I: [Chuckles] let us first hear your views on this first one. Receiving a reminder of your VMM appointment date.
152. R3: The message is a good thing, reminding them because they already came to the clinic. I will go back a little, I have already said that the people from the rural areas have questions and their questions might be a lot but because they don’t ask anyone those questions. They ask themselves and respond to the questions themselves as well. they can receive the message reminding them to come for VMMC and they would ask to say ‘why are they sending these messages? What do they benefit from this? I thought I am the one going to benefit from this but it is possible that they are the ones going to benefit because they are spending money and sending me messages.’
153. I: Okay, so the issue would still go back to the hospital benefiting somehow from the circumcision?
154. R3: Yes, they would because what they focus on is the benefit. I have heard that before to say ‘a very beautiful car came and they have picked up children including mine but what are they benefiting? Between someone who is seeking care and someone providing care, who has to put in more efforts?’ There are some questions which even if they were to ask you and you tried to respond, you would not respond right.
155. I: Alright, he thinks it might raise issues in terms of what the hospital is benefiting. What do others say, how would you feel about receiving SMS reminders?
156. R5: I would equally ask myself to say ‘why are they actually reminding me’? There is something going on.
157. I: Okay, anyone else?
158. R1: Just to add, I think the SMS would not really be helpful. That is because when we take a person from the rural areas, they use phones yes but all they know is to take calls. Even if there is a message, it is hard for them to check it. Apart from that, it could just worsen things, like airtel money for instance where people are stealing from each other by using messages, I think people are now ignoring messages from sources they do not know. Out of 20 people whom you have told that you will send messages, I think only 5 would follow through because messages are not usually attended to.
159. I: Okay, how would this go with culture?
160. R1: In terms of culture, it is what I said that if you were to approach the people. Approaching them is in different ways, one way for instance is to approach the chief of that area and you talk to him. You will introduce yourself and tell him what you want to do. that is why advisors that want to work in a village approach the chief first. When the chief says that come tomorrow at such a time, the people do come. I therefore think that if you approach the chiefs, it would be more effective than the SMS reminders.
161. I: Alright, the other strategy involves reimbursing transport. He spoke of how some people are reluctant to come because they will have to go back home on foot. So, if there was this transport reimbursement, how do you think it would go?
162. R5: That would go very well because the person will be assisted with that.
163. I: Okay, and so you are okay with this one? I remember you said no to the SMS reminders.
164. R5: Yes, if the people are told that when you go to the clinic you will be reimbursed, it would work.
165. I: Okay.
166. R6: This would help us because most people will be interested and they would come because they would say that ‘our friends went for VMMC and they were reimbursed.’ In that case, they wouldn’t be reluctant or lazy. They would come because they would know that after they circumcise us, they will give us transport money.
167. I: Okay, what do others think about receiving transport?
168. R3: This strategy could work depending on how the person who receives it understands it.
169. I: The one who receives the money?
170. R3: Yes, I remember there was a time when my neighbor had a child at KCH. What happened was that there was an organization which was helping women and they were giving them mk40,000 if I am not mistaken. As a result, women started running away with their children before they are healed because they would say that ‘this thing is strange, even my parents do not know it. I came here for assistance and instead of me being the one to produce the money, they are giving me money and telling me that they will follow up until I deliver. Other children however did not make it out alive, they died. Others would receive the money and they would… you know how life is at the hospital. My point is that it depends on the way they have understood it when they are told that they will be reimbursed. This method is helpful to those who have accepted it. They can get circumcised and maybe this should come out of you sympathizing with them. maybe after they are circumcised, you can pretend to ask what to do [how the person will get home] and from that angle, you can offer them the transport and not telling them that ‘go to the clinic, you will be given transport there.’ That will raise questions on why they are being given transport and yet they are the ones helping them.
171. I: Okay, are there any other thoughts?
172. R1: Yes, adding on what he has said, I was also looking at the aims, with what he is saying…I might disagree a little with what he has said. On this issue, considering what we have also been discussing, I think these strategies including the reimbursement one, with people, where there is money is where there are issues like unity and other things. The two things we talked about, going to the people in the rural areas and the reimbursement one, there is no human being who would refuse money. If people heard that transport is being reimbursed at Bwaila after circumcision, they would come. I feel like these two strategies; going to them and reimbursing, these two would get people interested in circumcision.
173. I: Okay, and not the SMSs?
174. R1: No.
175. I: Alright, with the reimbursement, what amount would be the most appropriate or what would we rather get instead of the money? Is the question clear?
176. R1: Yes.
177. R3: Just repeat it again because understanding differs, even in a classroom.
178. I: [Chuckles] okay, what amount would be appropriate to reimburse or if not the money, what would you have in place of the money?
179. R3: I think if we put a figure, we would be doing wrong. But, if the person has been taught and they are convinced to come for circumcision, they will come to the clinic not knowing that they will be given any money. After the person is circumcised, out of the pity of the one who circumcised the guy, they can give him money to say ‘add this to your transport money’.
180. I: Even if we give him mk100?
181. R3: No, that one is too little.
182. I: Yes, that is where the issue of how much is coming in.
183. R1: I think that currently in Lilongwe, the most expensive place to travel to are areas like area49, which costs mk700. If you gave the person mk2000, that is a lot to the person. Within Lilongwe, the most expensive transport cost is mk700 and if you give them mk2000, they will have some change. For people who live close like Mchesi, those who walk to the clinic will be at an advantage. In all that, the number of people coming will increase.
184. I: Okay, what do others say? The people this side, we are almost done but it will take longer if we don’t respond.
185. R5: I agree with what he has said.
186. I: The mk2000?
187. R5: Yes, because if you gave a lot more than that people would overthink.
188. I: Okay, is there any alternative to the transport?
189. R3: No, it should be the same money. I will agree with him to say everyone wants money. However, the money should not be as much as mk20,000 or so because people will start to question to say ‘have I sold my foreskin after all’?
190. R1: That is why I suggested the mk2000 because even travelling to Lumbadzi is mk2000.
191. I: Okay, number 6, you were saying something.
192. R6: I agree that they would think that they have sold the foreskin because of the money they are given. They would be doubtful and question their decision to do VMMC.
193. I: Alright, he said he would prefer to combine reaching out to people and the reimbursement. I have talked of 3 strategies; reimbursement, SMS reminders and education. Of these three strategies, which ones do you think would be the best combination or which one do you think would effectively increase VMMC uptake among men? Everyone will respond to this, we start with you.
194. R1: Like I said, I think the strategies that would work are the reimbursement. If the people come to the clinic you reimburse them but if you go out to the communities then you do not give them anything. So for me, it is the two strategies of reimbursement and education.
195. I: Okay, number 2, which strategy or which strategies do you think would work?
196. R2: [Silence]
197. I: We will come back to you, let us go to number 3.
198. R3: Three strategies have ben discussed. The first is education where you go out in the communities and start with dramas so that people come. The second strategy was sending SMS…
199. I: And the third one is reimbursement. What do you think about combining or if they were all being implemented at once?
200. R3: What I am seeing is that there are other things that when you discussing, a majority of people like them or not. In parliament for instance, they can pass one budget and decline another. Then, what happens is that when you try to implement the strategy that you chose, it does not work…
201. I: So I am asking which strategy of the three you think would be most efficient or…
202. R3: I think the strategy that would work of all the strategies that we have discussed… you need to try these out, implement them. when you implement them, you will not which strategy is working and the one that is not. Afterwards, you will take the strategy that is working and take that one.
203. I: Okay, but from our discussion of the three strategies, I want your thoughts on what would work… alternatively how do you feel about implementing these strategies at once? Your thoughts,
204. R3: Umm, if this were implemented it would be quite good.
205. I: Why?
206. R3: Because if this were implemented… I think I will pass on this question
207. I: Okay, number 4, how do you think implementing all these would go?
208. R4: [Silence]
209. I: Or which strategy or strategies would you choose to combine or implement individually?
210. R4: None.
211. I: Okay, number 5?
212. R5: I would opt for the education and after the person comes, reimbursing them.
213. I: Why these?
214. R5: Because when you teach the person how everything go, they will go home and then decide to come. When they come, you reimburse them.
215. I: Okay, what are your thoughts number 6?
216. R6: Aa, what they are saying is true. If you teach the person, they can also teach others and they can come back to the clinic with the friends so that even the friends can get better understanding.
217. I: Okay, I don’t know if there is any question or anything you would like to add. Before that, number 2,
218. R2: [Chuckles] it has already been said.
219. I: But to you, which of the strategies would you prefer to be implemented at this clinic?
220. R2: The strategy is the same one.
221. I: Which same one?
222. R: Reimbursing transport so that more people can come. Some live far from the clinic and the thought of walking to and from is just not right.
223. I: Alright, I understand. Is there anything you would like to add?
224. R5: there is. it is possible that what I want to say has already been said, but the issue is having a lot of community education. That is because the ones that live close to town are often busy, since they are here in town to earn a living. For them to eat, they need to work. These are not easy to reach. However, the hardest group of people to teach are the ones in the rural areas. What is needed is to find 2 experienced people who would go in the communities and you would approach the chief to say ‘we wanted to talk to it. The king would then ask his young men to so go spread the news in the communities and the people would come. When they are all gathered, you teach them the benefits, the advantages and as already said that they will experience the negatives after they have been circumcised.
225. I: Alright, as I said, the questions that I had are finished. thank you very much for your time.
226. R[Chorus]: Thank you very much.

THE END
