# Supplementary material for: Acceptability, feasibility and appropriateness of intensified health education, SMS/phone tracing and transport reimbursement for uptake of voluntary medical male circumcision in a sexually transmitted infections clinic in Malawi: A mixed methods study
Source: PLoS One. 2025 Jan 24;20(1):e0301952. doi: 10.1371/journal.pone.0301952 (PMC11760565; doi:10.1371/journal.pone.0301952)
Supplement: S1 Data — (ZIP) [file pone.0301952.s004.zip › Qualitative data/Endline IDI Transcripts/Transcript 13.docx]

1. I: So first of all, tell me more about your role at this clinic.
2. R: I am a (withheld), I test for HIV as well as provide HIV counselling.
3. I: Okay, apart from testing and providing counselling, is there anything else you do?
4. R: We also give out Oraquick. That is all.
5. I: Okay, but you meet every client that comes to this clinic or you choose who to counsel and who not to?
6. R: We meet every client because this is the entry point of this clinic.
7. I: Okay, so how open do you think both the male and female clients would be to talk about circumcision?
8. R: I did not get the question.
9. I: How open do you think the male and female clients who come here would be to talk about circumcision?
10. R: [Chuckles] it depends…it also depends on how open you as the provider are. if the provider is open to the patients, the patients also open up to you. as such, it all depends on you the provider, on how open you are with them. if you seem tough to them, it is hard for them to open up.
11. I: Okay, I see. So, just thinking about it, for every patient that you meet, even if they are open or willing to open up, so long as the provider is not open, the patient will not open up either?
12. R: That is what we can say. For most patients to open up, it all depends on you as the health worker. In HTS we say that for someone to open up and tell you their risks depends on how open you are with them. if you are not open or if you don’t show that you have welcomed them, they feel like ‘how can I open up and tell her my concerns’. I think the same would be the case with circumcision, the openness of us the providers. There are other people who are not open, maybe due to the different traditions, but there are not many people who are like that.
13. I: Okay, and let us go specifically to the men, how do you think they would react to someone talking about circumcision in a setting like the waiting area or the shelter if that is what it is called.
14. R: Yes, the shelter.
15. I: Yes, how would they react?
16. R: I think that they react well to it. That is because when the talk has been given and they come into our clinic rooms, some ask to say ‘they talked about circumcision while we were outside, what can I do to so get it’. I can say that ¾ of the people react well to it.
17. I: Apart from the health provider being open, why else do you think they react well?
18. R: Currently, circumcision is not something new, I can say it is something that everyone is talking about. Because of that, it is a habit now to say there is thing called circumcision and so it is everywhere. That is why it is not something new for the patient.
19. I: Okay, is there any other reason? [chuckles]
20. R: no [laughs]
21. I: How open are you to talk about circumcision?
22. R: [Chuckles] I have no problem with that because it is part of my work. I just have to tell them what circumcision is and the role it plays.
23. I: Okay, yes it is part of your work, but if you had an alternative, would you choos to talk about circumcision?
24. R: [chuckles]
25. I: Why?
26. R: I thought we want people to get circumcised! That is all… let’s just say because we want people to get circumcised. I know the benefits of circumcision and so we just have to give them information on circumcision and sensitize them to come.
27. I: Alright, on this issue of talking about circumcision, we are thinking of having intensified education at this clinic. This education will stress on what circumcision is, it’s benefits, as well as misspelling any misconceptions that are there. We might even invite men and their wives who have undergone VMMC to come and talk about it.
28. R: Alright.
29. I: What are your thoughts on using intensified education as a way of increasing VMMC uptake at this clinic?
30. R: It is fine. It is fine because when you give most patients examples or testimonies if I can call them tat concerning VMMC to say ‘I also got circumcised’, some get motivated and start to see it as something that is possible. So, it is fine.
31. I: Okay, and you are buying the idea of bringing in someone who underwent VMMC?
32. R: Yes!
33. I: Okay, and for the education, where we are just teaching, do you think it would increase uptake of VMMC or not?
34. R: the education…. [chuckles] this is hard
35. I: The intensified education, do you think it can help increase the uptake of VMMC?
36. R: Yes, it would increase uptake. That is because when teaching, we teach what circumcision is and its benefits. This family that is coming in for instance just motivates the people or encourages them to go for circumcision since other people have misconceptions around circumcision. They say that ‘we heard our foreskin is taken abroad and they use it for different things’. This couple therefore comes in to misspell the misconceptions around circumcision so that people can actually get circumcised.
37. I: Okay, thank you. Secondly, we plan to send SMS reminders to men who have a circumcision appointment. The SMS text will be carefully worded or coded for confidentiality. The messages will be sent three times; two days before the appointment, a day before and on the day of the circumcision appointment. What are your thoughts about using SMS tracing as a strategy for scale-up of VMMC at this clinic
38. R: It is also a good strategy. It is a good strategy because most people are here to earn money. With that, they might not keep their appointment date and so reminding them is a good strategy. However, you might have some challenges at times. That is because patients are difficult people at times. When reminding them, we should just expect to be insulted in terms of how they will respond to the SMS. Some might respond rudely. However, it is a good strategy because everyone forgets.
39. I: Okay, and so it would help in reminding the one who has forgotten?
40. R: Yes! A lot of people are busy looking for money and so they cannot easily remember their hospital appointment dates.
41. I: Okay, apart from receiving insults as responses to the messages, what other challenges can you think of with this strategy?
42. R: What?
43. I: Apart from the people who might not respond right, what other challenge can you think of regarding this strategy.
44. R: Aa, that is all. That they might respond rudely to the messages. They might call back or send a message in a very bad tone because humans are unpredictable. I am saying this because I have some experience. At Lighthouse, we have what we call…what do we call it? Active Index Testing, AIT. We call them so that we can test them for HIV. In the process, before you even introduce yourself to other people or explain why you have called, they start insulting you without even knowing why you have called. Others will still insult you even after you ae told them why you called. So, we should expect the same because the way we reason as people is different.
45. I: Okay, what solution can you think of to that?
46. R: [Laughs] there is no solution since we still need to remind them.
47. I: Okay, and there is no alternative as well?
48. R: No, there is not. That is because even if we give the people referrals, they tear them apart and throw it away. This one is a good strategy just that… I cannot think of any alternative.
49. I: Okay, we are also proposing to provide transport reimbursement to men who will undergo circumcision to help with expense incurred on the day of circumcision. The reimbursement will be an equivalent of $10 in Malawian Kwacha based on the National Health Sciences Research Ethics Committee guidelines. The reimbursement will be from a designated nurse within the STI clinic. What are your thoughts about using transport reimbursement as a strategy for scale-up of VMMC at this clinic?
50. R: It is fine. It might also… people tell each other things and when they tell each other to say ‘there is money being given’, the turnup for those who are willing to get circumcised will be high. everyone is need of money at the moment and with this opportunity to earn money without working for it, a lot of people would come to get the money [chuckles].
51. I: Okay, and you have said that people tell each other.
52. R: Yes, they would tell each other; people tell each other.
53. I: Okay, and if the community heard that there is reimbursement being given after circumcision, apart from other people coming to the clinic, how else would they react?
54. R: Some will be quick to think that it is satanic. The same issue of misconceptions that we talked about, not everyone has heard about circumcision, there are other people who do not know about it, they do not have enough information on circumcision. When they hear to say ‘we went to the hospital for circumcision and they gave us money,’ there will still be some people who will discourage them to say ‘that money is satanic. The foreskin they have cut off will be used for this and this’. Such people will be there.
55. I: Okay, what else do you think?
56. R: Nothing [chuckles]
57. I: Alright, in the end, we will want to implement all these strategies at once. We started with Intensive education and then SMS reminders and finally transport reimbursement right?
58. R: Yes.
59. I: But eventually, we will want to implement all the strategies at once. What are your thoughts on implementing all these strategies at once?
60. R: What?
61. I: Okay, the three strategies I have explained were being implemented one at a time, right?
62. R: Yes.
63. I: First was education, then we came to SMSs and then transport reimbursement.
64. R: Yes.
65. I: And so eventually, we will want to implement all of these at once and not one at a time. Do you understand?
66. R: Yes.
67. I: So, what are your thoughts on that? Implementing all the strategies at once; how do you think that would work out?
68. R: [Silence]
69. I: AT first, when a person comes, they get the education on circumcucision and that is all. The next person who comes will only be part of the SMS reminders and the last one will only be reimbursed without the other two. So, what we will want to do is that someone will come, get the education, then get the SMS reminders if they are willing to go for VMMC and then they will get reimbursed after they have undergone VMMC. What are your thoughts on that?
70. R: It has no problem [laughs].
71. I: Okay, why do you say that?
72. R: Because it will reduce the workload.
73. I: It will reduce the workload?
74. R: I don’t know whether I should say it will reduce the work or the time but… [chuckles] maybe I do not understand the question.
75. I: Okay, [chuckles] should I repeat the question?
76. R: [Chuckles] you should proceed.
77. I: Do you think it would be too much work or not?
78. R: Umm, what you are saying is that a person comes…I get the education on circumcision and then I have opted to undergo VMMC and if I am eligible to go through with it, because there are others who cannot be circumcised because of some diseases. So if I am eligible, I will get circumcised and then get reimbursed on the same day and the SMS is sent the same day?
79. I: No. let’s say people have come today and they are at the shelter and then someone does the intensive education. Afterwards, lets say maybe 6 people have agreed to circumcision, it means they will be given appointments and messages will be sent as reminders. When they come for the actual circumcision, they will be reimbursed. This is unlike where if they have had the intensive education it ends there, they will not be reminded or reimbursed. Or, the one who has been reimbursed has not had the talk or reminders.
80. R: Okay!
81. I: Yes, so for the last part, we will want to have all the strategies at once. How do you think that would work out?
82. R: It would work out well.
83. I: Okay, and I will ask again why you think so.
84. R: [Laughs] that is because the person…what I mena is that we will not loose people because this person has the information them we have sent the reminders and the person has been circumcised, it is the same person. In that way I can say that…I can say that follow up or whatever will be good because it is the same person and they know the things.
85. I: Okay and we wont loose out on people?
86. R: No, we will not lose any people.
87. I: Okay, do you think it would be too much work on the clinic staff?
88. R: [Laughs] I don’t know!
89. I: [Chuckles] but what are your thoughts; do you think it would be too much work or not?
90. R: Aa the clinic is not busy and it is not a lot of work [chuckles] I don’t know.
91. I: Okay, if you were to chose to combine two of the three strategies we have talked about which you feel would be more effective, or if you were to choose one, which ones or which one would you opt for?
92. R: Between the reimbursement…
93. I: SMS and education…
94. R: I would opt for reimbursement.
95. I: Why?
96. R: Like I said, people will target the money and so the turnup will be high. everyone is after money and so no one would refuse.
97. I: Okay, unlike the SMS one?
98. R: Yes, unlike the SMSs. The reimbursement one is better [laughs]
99. I: [Chuckles] okay. Considering what already happens at this clinic, do you think implementing these strategies is feasible or they would just be confusion?
100. R: No, there would no confusion. They would be fine.
101. I: Why do you say that?
102. R: Because the nurses that side already do some of the things that have been discussed. I think they already reimburse transport and they already provide information on the studies there. So, I think there would be no difference.
103. I: Because the things already happen?
104. R: Yes, because they are already happening.
105. I: Okay, then let us think of the wider community. If they learnt that there are these things happening at the clinic; that there is intensive education, SMS reminders and even reimbursement after circumcision. How do you think the community would react to these strategies if they were being implemented here?
106. R: It is like I said, not everyone would react the same. There are some people who like to discourage others. I already say that people misunderstand issues of circumcision. Still, its not like people would … I should say that people would still come.
107. I: They would still come?
108. R: Yes, they would still come.
109. I: In terms of religion and culture, if they heard of this, they would still come?
110. R: They would still come and currently, they talk of circumcision even in the churches. I think the religion that was difficult was Nyau but I think that even they take part in circumcision.
111. I: Okay, and so it is fine.
112. R: Yes, it is fine.
113. I: Okay, have you seen any of the strategies we have talked about being implemented at this clinic? The intensive education, SMS reminders and reimbursement, have you seen it happening?
114. R: We see the education being given every day, we see it being done at the shelter. For the SMSs, I am not so sure because I don’t know who sends the messages, maybe it is happening. For the reimbursement, I know it should be happening because when we have our weekly meetings, figures are presented of people who have undergone VMMC. So, it is possible that they were reimbursed or that the SMSs were sent. I don’t really make it a point to see what is happening and so it is possible that those things are happening. The education however is definitely done.
115. I: Okay, how do you think it is being done, from your perspective. We will only talk about the education because that is what you have observed. How do you think it is being done; is there anything you would like changed and things like that.
116. R: I think that there needs to be… we call it… I think there should be a manual, the thing we use when teaching. There is need for… I don’t know how to describe it.
117. I: Something like a guide?
118. R: Yes, I can call it a guide. There are times you are asked a question and you don’t know what to respond. However, when you refer to that thing, you are able to explain. I will give an example, there was a time when we were giving group education at Lighthouse to those newly tested positive and we had the thing I am talking about, am not sure whether it was called a manual or something… it had several things on it and whenever there was a question you were not sure about, we could use that to respond. I think something like that is needed because as it is right now, it is all head knowledge and nothing to refer to and I think the one giving education can use that to refer.
119. I: How would that help?
120. R: As humans, sometimes we can skip some points that are important.
121. I: Okay, points you would note if you are referring somewhere.
122. R: Yes, and its not like the people should actually see that you are just reading, but it helps to guide you so that you know that from this point I will go to this.
123. I: Okay, I see. What else would you like to change apart from the user manual, I will call it that for now.
124. R: Okay.
125. I: Yes, is there anything else that you would like changed?
126. R: No, that is all. That is the only thing I would like to see [laughs]
127. I: Okay, and considering the resources that are already available at the STI, which strategy of the ones we have talked about do you think would work?
128. R: [Phone rings, asks to be excused]
129. I: Okay, I was saying that considering the resources that are there at this clinic; of the three strategies we have discussed namely SMS, Reimbursement and Education, which ones do you think would work?
130. R: Which ones would work?
131. I: Yes, which ones are feasible?
132. R: They are all feasible.
133. I: Okay, all of them?
134. R: Yes, for the SMS all you need is a phone [laughs], the reimbursement will depend on the budget and how the sponsors have provided support. So they would work.
135. I: There would not be any challenges?
136. R: No.
137. I: Okay
138. R: As long as the resources are there.
139. I: With the resources that are available, think of the resources that are already there right now.
140. R: Okay! All of them would work. They already have the money that side and I believe they have a phone since the other studies they are conducting also use phones. There is already someone to give the education as well so they would all work.
141. I: Alright, I understand. Is there anything you would like to add?
142. R: No.
143. I: Any questions for me?
144. R: No, I don’t have.
145. I: Alright, thank you very much for your time.
146. R: Thank you.

TH END
