# Supplementary material for: Acceptability, feasibility and appropriateness of intensified health education, SMS/phone tracing and transport reimbursement for uptake of voluntary medical male circumcision in a sexually transmitted infections clinic in Malawi: A mixed methods study
Source: PLoS One. 2025 Jan 24;20(1):e0301952. doi: 10.1371/journal.pone.0301952 (PMC11760565; doi:10.1371/journal.pone.0301952)
Supplement: S1 Data — (ZIP) [file pone.0301952.s004.zip › Qualitative data/Endline IDI Transcripts/Transcript 12.docx]

1. I: Alright, first, tell me about your role at this clinic.
2. R: I am working as an (withheld), but I am a (withheld).
3. I: Okay, what do you do as an (withheld)?
4. R: As an (withheld), we help people who have sexually transmitted diseases and we also encourage those who have sexually transmitted diseases but have not undergone circumcision, we encourage each man to go for VMMC so that they should be protected from sexually transmitted diseases.
5. I: Okay, and when you talk to these men about VMMC, how do most of them react?
6. R: Umm, most men who have not undergone VMMC agree to do so, they agree to go through VMMC. However, here at Bwaila, we have one challenge. Previously, when we find a man who wants to go for VMMC, we were sending them directly to the VMMC clinic and they would get circumcised. Currently, they are given appointments on when they should come or they are told where they can go to get VMMC. So, we have that one challenge currently. Unlike previously where when they have accepted, they were sent to the VMMC clinic right away.
7. I: Okay, where do you think the challenge is in giving them appointments?
8. R: When they have been given the appointment, the challenge is that when they go home, there is a lot to do. Some will tell you that I failed to go because I went to a funeral or I went to the village. So, its like you give them a chance for them not to come. But, when everything is being done here, they get everything done and then they go home.
9. I: Okay, but what do you think makes most of them agree to undergo VMMC when they are told about it?
10. R: When most of them are told of the benefits of VMMC, that they have a 60% protection from diseases like HIV and other sexually transmitted diseases, they are able to understand. For some, their sexually transmitted diseases are in their foreskin and when they are told that circumcision also helps, they feel it is better to get it done so that the diseases do not come back. So, most people understand when they are told of the benefits of circumcision for their body.
11. I: Alright, and how long have you been an STI coordinator?
12. R: I have been an STI coordinator since 2014, which is about 7 years…
13. I: 7 years.
14. R: Yes.
15. I: Okay, and so these men you are talking to are the ones who have come into your clinic rooms right?
16. R: Yeah, the ones who have come for treatment because they are sick, these are the ones we tell.
17. I: Okay, but these people first sit in the waiting area and it is a mixture of men and women. If you were to talk of circumcision in that setting, how do you think they would react?
18. R: It is also good if they come as families and when the group also consists of women, it is fine. That is because when the women go home, they encourage their husbands if they have not undergone circumcision to say ‘there is circumcision being done at the hospital for people like you and you can be assisted’. That helps because the women have information which they can actually tell their husbands at home.
19. I: Okay, and that really happens? women who come here take the information to their husbands, that really happens?
20. R: Yes, it happens.
21. I: Alright, and how open are you to talk about circumcision?
22. R: With regards to circumcision, I am open to talk about it because with the nature of our work, for everyone who comes with an STI, we still examine them if they are men. After examining them, we also check to see if they are circumcised or not and we have a chance to advise them during the examination since we are able to see if they are circumcised or not. And so we offer them counselling.
23. I: Okay, so it is something you are already talking about and you are okay with it?
24. R: Yes!
25. I: Alright. first, we are proposing to conduct intensified health education on circumcision at this clinic. Intensified health education will be in regular group health education talks on circumcision. The education will focus on what circumcision is, its proven benefits and common misconceptions about circumcision. We will also allow patients to ask questions about circumcision. We propose to also involve men who have successfully undergone circumcision and as well as their spouses to share experiences around circumcision. What are your thoughts on using this education as a way of increasing the uptake of male circumcision at this clinic?
26. R: I think this is a good thought, especially the part of using the women. That I because when the women come with their husbands and they vouch for the circumcision, that will encourage other women to bring their husbands. They will reason to say ‘if this woman says she is happy’, because the women will be motivating their fellow women. They would say ‘before my husband was circumcised, things were not going well. however, after circumcision, everything is now fine and so I encourage you to let your husbands get circumcised.’ The other women will therefore be motivated because of what the other woman is explaining. So, the idea of bringing families is good. The health education is also good because it would mean that those who had misconceptions will be cleared after hearing from their friends who have undergone circumcision. Therefore, they would be motivated.
27. I: Alright, apart from what I have mentioned, what circumcision is and tackling the common misconceptions; what else can be included in the health education?
28. R: Umm, the other things fall in those groups as well, to tell the what circumcision is. however, you could just stress on the benefits of circumcion. At first, people misunderstood circumcision. They thought that oce they are circumcised, they will never suffer from any other STI’s like urethral discharge, syphilis and the like. So, what happened was that after circumcision started, we had a lot of young people with urethral discharge but circumcised. After talking to them, I told them to tell their friends that circumcision only protects them 60% and they will have to use condoms for the rest of the protection. after explaining to them, the number of young men coming with urethral discharge started to decrease. But, because they had little knowledge, they thought that they will not suffer from any disease because they are circumcised. So, that part should be stressed to say it does not mean that one will not suffer from any STI’s or HIV just because they are circumcised. People say that ‘I can do whatever I want! I am protected now and I will not contract any disease!’ so, they need to be told that they are only being protected 60% and for the 40%, they have to use condoms.
29. I: Alright, the second thing we are proposing to do is to send SMS messages to the men who have been given a VMMC appointment date. The aim of these SMS’s is to remind the men of their appointment. The messages will be sent three times; two days before the appointment date, a day before the appointment date and on the day of circumcision. What are your thoughts on using SMS reminders as a way of increasing VMMC uptake among the men?
30. R: I this that one is also a good method. Some people forget when they have been given a date and so when you send the SMS, it is a reminder. When you send the SMS, if they had not washed yet, they will make an effort to wash knowing that they have to go to the clinic the next day. If you remind them on the actual date it would be tricky. However, reminding them two days before is quite good. As the day approached, the person is aware that ‘I am going for circumcision tomorrow’. I think this one is also a good strategy.
31. I: Can you think of any challenges with this method?
32. R: The challenge could be the phones. Some can give wrong phone numbers, some would switch off their phone thinking you will trace them and so when you call, the number is never available or they do not pick up. At times, they can even give wrong direction. However, someone who is interested gives the right information in terms of the phone number or the map. The only challenge is when the phone is off or if they switched it off on purpose or are not picking up. It is difficult.
33. I: Okay, can you think of any way of dealing with those challenges?
34. R: Aaa, I do not know if there would be ways of tracing the person such that when they are giving the phone number, they also give directions to their house so that if they do not respond to the SMS and a couple of days have gone by, someone can follow him up to see if he is still around or if he moved or whatever reason.
35. I: Okay, so you are saying we should get directions to the person’s house the same time we are getting the phone number?
36. R: Yes.
37. I: Okay, is there anything else? As another way to deal with the challenges.
38. R: Aa, no.
39. I: Okay. The last strategy we are proposing is to provide transport reimbursement to men who will undergo circumcision to help with expense incurred on the day of circumcision. The reimbursement will be an equivalent of $10 in Malawian Kwacha based on the National Health Sciences Research Ethics Committee guidelines. This reimbursement will be from a designated nurse here at the STI clinic. What are your thoughts on using transport reimbursement as a way of increasing VMMC uptake at this clinic?
40. R: That is quite a good thought. The reason is that like I said, we are not doing VMC here. as such, someone might want to undergo it but they might fail to come because they do not have transport to get to the clinic or to go back home after. However, if you tell them that they will be reimbursed, they will be encouraged and can plan to borrow some money knowing that they will refund after they come from the clinic. That will therefore help other people who might have not had any transport money to come to the clinic for VMMC. That is quite a good thought.
41. I: [Chuckles] okay
42. R: However, one challenge would be that others would get circumcised simply because they want the money [chuckles]. That is a disadvantage. For those who are willing, it is a good strategy in cases where they do not have transport.
43. I: Okay, so those who only ant the money will still get circumcised but what they are after is the money.
44. R: Yes.
45. I: Okay, any other challenge that would be there with transport reimbursement?
46. R: Aaa, I don’t think there is any other challenge. That is the only challenge I can foresee.
47. I: Okay, eventually, we would want to implement all these strategies at once. That is the intensive education, SMS reminders and the transport reimbursement. How do you think implementing all strategies at once would work out?
48. R: That would be very helpful because everyone who has undergone circumcision will benefit. If there is only one strategy, it means one will only come for education, another SMS and another transport reimbursement. However, when these are all being implemented, everyone will gain the same…they will have similar packages. So, it would also be good for uniformity sake. That is everyone should go through the intensive education, get the SMS and be given money at the end.
49. I: Okay, do you think implementing all these at once would have any impact on the people coming for VMMC?
50. R: Yes, but I think the numbers would really increase with the issue of transport reimbursement. People keep saying that ‘things are not fine’ and so if you reimburse them, it would make things better since they have transport. Unlike the SMS, they can easily say ‘I don’t have transport’ and with health education, they would say ‘I want to come but I don’t have transport.’ So, if you give them transport, they will have no excuse. This strategy would help a lot of people get circumcised.
51. I: Okay, and in terms of workload, how do you think this would go?
52. R: Aa, currently I am not sure how the turnup is at the VMMC centers. I do not know how many people are being circumcised or are going to the centers per day. As such, I would not really be able to respond to that one unless I went to the VMMC centers to see the turnup of people. I do not know if the numbers will increase or decrease because I do not know how any people are already going there right now.
53. I: Okay, considering what already happens at this clinic, how do you think these strategies line up with what already happens?
54. R: The reimbursement will make a lot of people come to the clinic unlike the way the numbers were. A lot of people would come.
55. I: Okay, when we look at culture or the different religious beliefs, do you think these strategies would be in conflict with any cultural or religious beliefs?
56. R: At first, we had a lot of problems here in Malawi. Most people who were being circumcised needed to be Muslims because the Chewa were claiming that it is not their culture. With time however, as people understood that they can be protected from STI’s, people started to understand and at the moment, it does not even depend on the culture of the person. Whether Muslim, Yao, Chewa or whoever, everyone is free to get circumcised. A lot of Chewa’s are also getting circumcised right now. I think that because of sensitization… when something is just starting, people are often afraid of it. With time however, they start to understand it and to follow it. It is hard for us Malawians to understand things right away, we understand things slowly. In terms of culture, we can say that it is better now unlike the way it was in the past. People now understand circumcision.
57. I: Alright, those are the questions that I had today unless you have anything you would like to share with me concerning VMMC, what would help increase its uptake or anything.
58. R: I really wish VMMC was still here at Bwaila. Of course, I don’t know what really happened for it to close but the closure of the center affected the activities of VMMC. That is because when the people come, there are some who want to get circumcised. People approach me asking where to go in order to get circumcised and I refer them to someone. They come back and tell me that ‘I have been given such a date’ and yet if the center was right here at Bwaila, that person would have been assisted but you are re-scheduling the person to another day. So yes, if there was space here so that people would get circumcised, it would have been helpful because Bwaila is like the center. When people get off the bus, they are already in town and it is easier unlike telling them to go elsewhere, that disturbs the people in a way. Yes, those are my last words.
59. I: [Chuckles] alright. you have reminded me of another question.
60. R: Yes.
61. I: Apart from the people being referred to other clinics for VMMC, if these strategies were made part of the daily routine here at VMMC, do you think people would still be missed or would the numbers increase or would it still be the same?
62. R: The missing out comes in because when someone comes to the clinic, they come with the mind that I am going to do such and such at the clinic. When they come to the clinic and you tell them that the service they wanted is not available, you disturb that person and they tell you that they need to go back home and re-think yet when they were coming on this particular day, they had already decided. So that is the problem.
63. I: Okay, so even if these were put in place, the problem is where we are telling the man to go to another clinic?
64. R: Yes!
65. I: Okay, I understand [chuckles]
66. R: If for instance I am ready today to get a family planning method, I come here and they tell me to go to Kawale, do you think I will go straight to Kawale?
67. I: You will go home.
68. R: Yes, I will go home and while there I might need to go for a funeral at the village and when I am coming back from the funeral you find that I am pregnant [laughs].
69. I: [laughs] alright.
70. R: So you see, that is the whole issue.
71. I: Alright. Thank you very much for your time today.
72. R: Thank you.

THE END
