# Supplementary material for: Acceptability, feasibility and appropriateness of intensified health education, SMS/phone tracing and transport reimbursement for uptake of voluntary medical male circumcision in a sexually transmitted infections clinic in Malawi: A mixed methods study
Source: PLoS One. 2025 Jan 24;20(1):e0301952. doi: 10.1371/journal.pone.0301952 (PMC11760565; doi:10.1371/journal.pone.0301952)
Supplement: S1 Data — (ZIP) [file pone.0301952.s004.zip › Qualitative data/Endline IDI Transcripts/Transcript 14.docx]

1. I: First of all, tell me more about your role at this clinic.
2. R: I work as a (withheld) at this clinic; I test people for HIV.
3. I: Okay, apart from HIV testing, is there anything else that you do?
4. R: The other things is that when someone tests positive for HIV, we escort them to the right place so that they can be initiated on ART. Before escorting the person, we ask them whether they are married or not, we ask them to tell us their sexual partners or their children so that they can also get tested and know their status. This helps so that HIV should stop spreading amongst us, we are cutting the web in other words.
5. I: Okay.
6. R: We also do recency which has just started. It was there previously but it stopped due to COVID and it has just re-started. The aim is so that of those we have tested and found HIV positive we should know how many of them contracted it last year. When such people have been identified, we collect more blood samples to send to the lab so that they can confirm whether it really a new infection or not. If it not a new infection, we leave them but if it is new, we collect the blood sample. This is so that the government can track where most new infections are coming from and they should find a way of reducing the spread of HIV infections in that area.
7. I: Okay and you said it is called recency?
8. R: Yes, recency.
9. I: Okay, but you attend to every client right? You do not only attend to females or males right?
10. R: No, it is a mixture. We just share the health passport books amongst ourselves and so it just depends on the group that you have been given. You can either have a lot of males or females, it is just a mixture.
11. I: Okay. From your knowledge of the type of patients that you see, whether male or female, how open do you think they would be to talk about circumcision?
12. R: The people are quite open. At times, we ask them if they are circumcised and they are able to respond to say ‘yes, I got circumcised at such a hospital’ or some would say ‘yes, I got circumcised traditionally.’
13. I: Okay, without asking them about it, would they bring it up?
14. R: No, mostly we have to ask them. however, some people come to say ‘I want to get circumcised, what do I need to do?’ and we refer such people to the person responsible.
15. I: Okay, and if someone were to explain to them about circumcision, how do you think they would react?
16. R: Aa, most people do not seem shy about it. They even ask to say ‘does it mean that I will not contract HIV if I am circumcised?’ and we respond to say that is not the case, it only helps to reduce the risk of contracting HIV by 60%.
17. I: Okay, what do you think helps them open up?
18. R: It could be because of the disease that has brought them to the clinic. Most diseases that come here involve the sexual organs and so some take this as an opportunity to ask all those questions which they were failing to answer on their own.
19. I: Okay, and how open are you to talk about circumcision?
20. R: I am open to talk about it because with our work here at the hospital, we say that we should do any kind of work that we have been given and we work depending on the people that have come on that day. On some days you find that there is a lot more women than men but when there are men in the group, we still offer them. some say they are already circumcised while others say that they were born circumcised. Of all the men that I have seen, there was one who told me that ‘I was born that way (Circumcised).
21. I: Okay, you have said that you do any kind of work that needs to be done right?
22. R: Yes.
23. I: But if you had the option of choosing one type of work over another, would you opt to be involved in talking about circumcision or not?
24. R: I would opt for it because it is also one way of fighting HIV and preventing it from spreading among us. you can choose not to talk about it but you never know, the person you did not give this information to will sleep with your child or your relative and end up infecting them. But, if you take part and assist, you are also protecting several people from contracting HIV.
25. I: I understand. We are thinking of conducting intensified health education on circumcision at this clinic. Intensified health education will regular group health education talks on circumcision, like in the waiting area. The education will focus on what circumcision is, its proven benefits and common misconceptions about circumcision and we will also allow patients to ask questions about circumcision. We could also involve men who have successfully undergone circumcision and their wives to come and share experiences around circumcision*.* What are your thoughts about using intensified health education as a strategy for scale-up of VMMC at this clinic?
26. R: That is very good as well and especially if we involve the women sice they are the ones who live with the men in the homes. It is usually hard for men to come to the hospital. whenever they have a problem, they rush to the pharmacy to buy medicine and at times they may buy medicine that is not suitable for the disease that they have. However, if we involve the women, they are the ones who live with the men in the households. Some men may be shy to open up to other people about their issues but they can easily open up to the wives or to their sexual partners even if they are not the wife.
27. I: Okay, and so you think that if we involve the women, they can bring it up in the homes and…
28. R: And that would help for men to come for circumcision.
29. I: And so for the education, how likely is it or what is the possibility of it to actually increase the number of men coming for VMMC?
30. R: It would increase quite some much because most times it is women who come to the clinic. When you ask the women if they have been tested before they will say yes but when you ask if their husband has been tested, they say ‘no, he has not. He refuses to come to the hospital.’ then we offer them the self-test kits and teach them how to do the test so that the men can test. We tell them that if the result is such, the man should come to the hospital for further assistance.
31. I: Okay, and so you think the numbers would increase?
32. R: Yes.
33. I: Okay, second, we are thinking of sending SMS reminders to men who have a circumcision appointment. The SMS text will be carefully worded or coded for confidentiality. The messages will be sent two days before the appointment, then a day before and on the day of the circumcision appointment. What are your thoughts about using SMS tracing as a strategy for scale-up of VMMC at this clinic?
34. R: It is also very good because in other cases, messages are sent to people to remind them to come and collect ARV’s for instance. That is because people are actually quite busy and they might forget. But, when they see the message they would easily remember.
35. I: Okay, and so you are likening it to ART reminders.
36. R: Yes.
37. I: But how do you think the issue of acceptance would play out or how would they react to this strategy?
38. R: I think it is not hard because at times, when we are up there, the time I was working in retention, we were also making such calls. That was mainly to those who were recently initiated on ART. We follow up on them to remind them of their refill day and their reaction is usually ‘oh yes, I actually forgot. When I find money I will save it so that I can use it for transport to the clinic’. That means this one can also be one way of preparing the people so that if they were busy or if they travelled, they should come back so that they can come for VMMC.
39. I: Okay, can you think of any disadvantage that can come about with this strategy?
40. R: The women who do not know how to read might think that the message is coming from another woman once they see it in their husbands phones. However, for people who trust each other, the man can easily explain that it is a VMMC reminder and they would actually be glad about it. I recall on Monday, I think it was on Monday, a couple came and the man was saying that whenever he sleeps with his wife he gets cuts and I offered them circumcision. They agreed to say ‘I think what you are saying is true’ and I said it would actually help reduce the problems that they are facing. He said that ‘when I am healed and I have finished the treatment they have given me, I will come back to understand more about how the circumcision might help me.’
41. I: Alright, one other thing we are thinking of is reimbursing transport to men who will undergo circumcision to help with expense incurred on the day of circumcision. The reimbursement will be an equivalent of $10 in Malawian Kwacha based on the National Health Sciences Research Ethics Committee guidelines. The reimbursement will be from a designated nurse within the STI clinic. What are your thoughts about using transport reimbursement as a strategy for scale-up of VMMC at this clinic?
42. R: It is a good strategy because it could happen that after we tell the person to come on such a day, with the way money is not easy to find and also because not everyone who comes to this clinic has the money for transport to come here, that would help the men to come to the clinic on time to get assistance. It would also help because when they go back home, they can tell others to say ‘I went for circumcision but they reimbursed my transport’. That would encourage others to say ‘let me go to the clinic for circumcision and they will give me transport there, I don’t have to worry’. That is because here in Malawi, money does not come by easily. Apart from that, there are people who have no means of making money. Those might reason and say ‘when I go to the clinic, I will be reimbursed and I will also have some change from that’.
43. I: Okay, and so you think this strategy would work and the numbers would increase?
44. R: Very much! It would work and a lot of people would actually come. The men would tell each other about it and the numbers would increase.
45. I: Okay, with this strategy, what disadvantages can you think of?
46. R: A disadvantage in terms of money?
47. I: Yes, any disadvantage you can think of concerning this reimbursement.
48. R: Aaa no, this one has advantages and not disadvantages. A lot of people would come.
49. I: Alright. eventually, we would like to implement all these strategies at once. That mean we will have the education, the SMS reminders and the Reimbursement.
50. R: Yes.
51. I: What are your thoughts on implementing all these strategies at once?
52. R: It is helpful because we will send an SMS to the person, the person comes and gets the education in the group setting and as you have said that those who have already gone through with it will come to share experiences, that one is good and it would help to bring light to the truth because people mostly tell each other lies in the communities.
53. I: Okay, do you think it would be too much to do? To have intensive education and SMS reminders and the reimbursement; think in terms of both the providers and the clients.
54. R: For the providers, today for instance, you wanted to have a focus group discussion and am the one who tested all of them. it’s just a matter of committing oneself to the work I can say.
55. I: Okay, so it would not be too much, the issue is just commitment.
56. R: Yes!
57. I: Okay, if you were to choose one which you feel would work better or if you were to combine, which one or which ones would you opt for?
58. R: The SMS reminders and the Reimbursement.
59. I: Okay, you would opt for those two?
60. R: Yes. However, the education one is also good. Specifically, where you are bringing in other couples who have gone through it to share their experiences.
61. I: Okay, let me start with the combination of SMS reminders and the reimbursement; why do you think these would work?
62. R: Because when they are reminded, they will come to the clinic and they will be assured that when they come to the clinic, transport will not be an issue. They can even borrow from other people to ay ‘borrw me some money, I will return it when I am back’. And indeed when they come to the clinic and they are reimbursed, the person they borrowed money from might ask where they got the money and in response they might say ‘I went for circumcision and they reimbursed me’. In the process, they are sharing the benefits of coming to the clinic and also that they have been reimbursed.
63. I: Okay, in implementing all of these at this clinic, you have said that it is possible and that the issue is just commitment. However, how do these line up with what already happens at this clinic?
64. R: They are not very different because when we talk of the education, it is the same thing that already happens there and so they will simply continue on what already happens.
65. I: They are not different.
66. R: No.
67. I: Okay, and considering culture and the different religions that are there, how do you think these strategies line up with those?
68. R: Some people might think that because they are actually calling the men to come for circumcision, it means they want to do something with the foreskin, you know how people reason. They therefore need to be encouraged on that aspect and told that when the foreskin is cut, it does not go anywhere [external interruption]
69. I: Please continue, we were talking of the foreskin…
70. R: Yes, some believe that the skin wants to be used for witchcraft. However, when they come to the clinic and meet people who have undergone circumcision, they might learn that there is nothing wrong with it and that they can also go through with it.
71. I: But that would be after they have come to the clinic.
72. R: Even while they are still in the communities, some people go through circumcision at a young age as part of their culture. If we involved such people, it would be helpful.
73. I: Okay, so we should involve those who practice circumcision as part of their culture?
74. R: Yes.
75. I: How do you think that would help?
76. R: When they hear them say that ‘I was circumcised at a young age but there is nothing wrong with me’, it would help.
77. I: Okay, so it would be a way of encouraging the others?
78. R: Yes.
79. I: Okay. These strategies were being implemented at this clinic. The intensive education, SMS reminders and reimbursement has been happening at this clinic. What are your thoughts on how they have been implemented?
80. R: There is nothing wrong because this will make people come to the clinic. If they have STIs they will be treated and we will offer them the circumcision.
81. I: Okay, what else makes you say that there is nothing wrong or that they were implemented well?
82. R: When their STI is treated and they have also learnt about HIV, they will have benefited because they will protect themselves from contracting HIV and they will get circumcised. There are people in the communities who hear of circumcision but they do not know what to do to get circumcised. This would help so that those in the communities can have a chance to come to the clinic.
83. I: Have you seen these things happening at this clinic? The education, SMS reminders…
84. R: Yes, we have seen them happening and we have heard them say that the transport reimbursement has started. They said it during a meeting last week.
85. I: Okay, I understand. Is there anything which you feel could be done differently or anything that you would like changed from the way things have been happening. I am not sure how involved you are with the implementation of the strategies.
86. R: We are mainly involved in the screening when they coe here. we ask if they are circumcised and things like that but for the other things, we are simply told that ‘transport reimbursement has started’ and things like that.
87. I: Okay, so at the moment, is there anything that you think can be changed? It could be in terms of your involvement or anything that you feel could be done better.
88. R: If there are leaflets on circumcision or the strategies, it would be good if we also had them so that we have adequate knowledge and that when we speak of these things, we actually know what we are talking about.
89. I: Alright, and the three strategies that we have discussed, if they were implemented permanently and not as a study, like it is right now, how do you think it would work out?
90. R: It would go well because where there is money, you know how willing Malawians are when money is involved. As such, it would go very well.
91. I: Okay, the people would come?
92. R: Yes.
93. I: Okay, and in terms of the clinic, how do you think it would go?
94. R:We would asses how things are going. This thing is just stating and when something starts, we evaluate to see what is working. So, we can meet monthly or after two months to talk about how things are going and which areas can be changed.
95. I: Okay, just thinking about it right now in this moment, how do you think it would work?
96. R: It would be fine because it will be a part of the routine work. In the morning people are taught, those who want to be circumcised will be circumcised and those needing transport will be given. The work is not all being done by one person, one person works on this while another does another part and so it is fine. It’s not like the same person will be with the client from start to finish.
97. I: Is there any one strategy you would rather have implemented out of all these?
98. R: The SMS reminders are good if they were implemented. The reimbursement depends on the one giving the money [source of funding] because they might get tired of it one day. The SMS reminders are at least cheaper.
99. I: Okay, and so you would love if that one were implemented?
100. R: Yes.
101. I: Okay, apart from it being cheaper, why else would you opt for that one?
102. R: Because it is easy. The men are simply being reminded that you need to come to the clinic on such a date for circumcision.
103. I: Alright, alright. I think that is all I had, I don’t know if you have anything you would like to share with me.
104. R: Aaa, I don’t have anything to share.
105. I: Okay, or any questions.
106. R: There are no questions at the moment.
107. I: Okay, thank you very much for your time today.
108. R: Thank you very much.

THE END
