# Supplementary material for: Acceptability, feasibility and appropriateness of intensified health education, SMS/phone tracing and transport reimbursement for uptake of voluntary medical male circumcision in a sexually transmitted infections clinic in Malawi: A mixed methods study
Source: PLoS One. 2025 Jan 24;20(1):e0301952. doi: 10.1371/journal.pone.0301952 (PMC11760565; doi:10.1371/journal.pone.0301952)
Supplement: S1 Data — (ZIP) [file pone.0301952.s004.zip › Qualitative data/Endline IDI Transcripts/Transcipt 15.docx]

1. I: First of all, tell me about your role at this clinic?
2. R: Okay, as a (withheld) at this clinic, my role is to assist women who have reported with sexually transmitted diseases. I provide counselling, draw blood samples, work on study activities and whatever else comes, we work on that.
3. I: Okay, and how long have you been doing this?
4. R: At the STI clinic?
5. I: Yes.
6. R: Five years.
7. I: Okay, and so you attend to both male and female patients in your role, right?
8. R: Yes.
9. I: How open do you think the patients that come to this clinic would be to talk about circumcision?
10. R: Umm, it seems that they are open to talk about circumcision. Mainly the youth, the teenage generation or the twenties and thirties, these are very open unlike the forties.
11. I: Okay, why do you think that is the case?
12. R: In the past, people were not open, it was not there while for these ones, they start being open from a young age and so talking of these things to them is not something new. In the past, I can say according to culture, every issue to do with the private parts was regarded as something very secret and so that is how they grew up. That is why you find that they are not as open as the younger ones.
13. I: Okay, so it is about their upbringing.
14. R: Yes.
15. I: Okay, but then whether young or not, let us compare the males and the females; who do you think would be very open to talk about circumcision?
16. R: The men.
17. I: Why?
18. R: Men are always free when in a group. And for the women to also talk about issues to with the private parts, I will take it back to the issue of culture, they are not open to do that. They are not comfortable to talk about their own private parts.
19. I: Okay, I understand. In your case, how open would you be to talk about circumcision?
20. R: Ii 100%.
21. I: Okay, why?
22. R: Maybe because it is part of our work, we are used to it. The thing is, if you don’t talk of things as they really are, people interpret words differently. As a nurse, and as part of my job, I am used to calling a thing what it is. if it is an ear, I call it an ear and the same with any other part. So, I am very open to talk about it because I am used to it, it is like my daily language. It is unlike talking to someone who is not used to talking about the private parts, they would be very uncomfortable talking about them. but, in my case, I am used to it.
23. I: Okay, apart from it being your work and it being something you are used to, is there any other reason why you would be open to talk about circumcision?
24. R: Awareness, in order to increase awareness. People should know the truth. It seems people easily believe their friends, they easily believe the people they live with in the communities. They can come to the clinic to learn about family planning for instance, but they will hold what their friends have told them as valuable.
25. I: Why?
26. R: I do not know. They already have information which they have heard from their friends when they are coming to the clinic, but I do not know why they believe their friends.
27. I: Okay but you would do it to raise awareness.
28. R: Yes.
29. I: Alright, so we are thinking of having intensified health education on circumcision at this clinic. Intensified health education will regular group health education talks on circumcision. The education will focus on what circumcision is, its proven benefits and common misconceptions about circumcision. We will also allow patients to ask questions about circumcision and we propose to also involve men who have successfully undergone circumcision and their spouses (women) to share experiences around circumcision. From your observation, how do you think this has been going?
30. R: The intensified health education?
31. I: Yes, I think these take place out there.
32. R: Oh, the morning ones.
33. I: I think so.
34. R: Okay, I thin they go well because when they are giving the education, I think people are open and they discuss and they look interested in the conversation with the one teaching them.
35. I: Okay, going forward, what are your thoughts on still using this as a strategy to increase VMMC uptake?
36. R: It looks like it is helpful. It seems to be helpful. Mainly, when they see someone who is not dressed in a nurse’s uniform or any uniform, they take the person as one of them. so, if this were to continue, it would be a good thing. For the people I talked about as well, the ones who listen to what their friends, when they see someone dressed civilian and looking like one of them, they can take that as important.
37. I: Okay, is there anything you would like to change or anything to be added to the content of the education? [audio paused] you were saying that the people giving this education are dressed in civilian and so the patients take them as one of them. then I asked if there is anything you would like added to the way this education is being given?
38. R: Umm, maybe if posters were added, adding demos so that everyone has a chance to practice when they are being taught.
39. I: Explain that.
40. R: With condom demonstration for instance, so that a lot of people can have a chance to try out the condom demonstration.
41. I: Okay, how do you think that would help?
42. R: It would help because when it is another person doing it, you might think it is easy. However, when you try to practice it on your own, you will be able to note where you are going wrong and how to improve. Some people will agree that they have heard when they are in a group of people, but they would not be able to do it on their own. But, if everyone took part in the demonstrations, it would help.
43. I: Okay, is there anything else? Or what should be added to the content of this education?
44. R: Umm, I cannot think of anything at the moment.
45. I: Alright, we also plan to send SMS reminders to men who have a circumcision appointment. The SMS text will be carefully worded or coded for confidentiality. These messages will be sent two days before the appointment date, a day before and on the day of the circumcision appointment. How do you think this has been going so far?
46. R: I think it is going well, but it cannot go very well because the percentage of people with phones… [chuckles] I don’t know if 50% of people have phones. They can have the phone, bit you find that they did not charge it and so there are some hiccups.
47. I: Okay, so first is the percentage of people with phones and the other thing is the people with phones actually charging their phones. Are there any other hiccups with this one?
48. R: Umm, that is the only one. For people whose phones are always available, it is a good strategy.
49. I: Why is it a good strategy?
50. R: Because they are reminded in case they forgot. With the different work that we engage in, at time we tend to forget maybe because of how busy the work is and other things. You might be occupied with other things and for you to remember your appointment… besides, me forget appointments more than women do and so this strategy is helpful to those who have phone because they are reminded.
51. I: Okay, and what can be done for those who do not have phones?
52. R: Maybe if they gave contacts.
53. I: Contacts like?
54. R: If they can give a number of their friends who have phones. But with that then we would settle for calling and not sending the SMSs. It would also depend on what you agree with the patient concerning how you should address yourself when you call. When you agree, the person will immediately know that this is the hospital calling. That is because other people would not like it known that the hospital people were calling them and they said that there are two days before their appointment. So, for those who do not have phones, maybe they would give other contacts and if not, they would be followed to their houses to be reminded.
55. I: Following them as in tracing them?
56. R: Yes, physical tracing.
57. I: Okay, and what are your thoughts on the frequency of the messages; that they are being sent 3 days before, 2 days and on the actual day; what do you think of that?
58. R: I think it is fine.
59. I: Why do you say that?
60. R: Because some might forget that they received a message, they would just brush it off and say ‘I remember’. You can remind them today but you find that they have forgotten by the following day so it is good to remind them.
61. I: Okay, we are also proposing to provide transport reimbursement to men who will undergo circumcision to help with expense incurred on the day of circumcision. The reimbursement will be an equivalent of $10 in Malawian Kwacha based on the National Health Sciences Research Ethics Committee guidelines. The reimbursement will be from a designated nurse within the STI clinic. What are your thoughts on this strategy?
62. R: This strategy is also very good.
63. I: You have actually said ‘very good’ for this one.
64. R: Very good.
65. I: Tell me why.
66. R: That is because some might have the desire to go for circumcision but you find that they do not have transport money. And the amount of 10$ that you have mentioned, they would not only use it for transport. They can also use it for their other needs. Some say that ‘after I am circumcised, what will I eat in the week that I am nursing my wound’. Some people therefore would use it during that one week for the needs while they have the wound so that as the wound heals, they will have the strength to go back to looking for food. So, most people look at it from that angle and not just as transport. Because of that, they like this strategy.
67. I: And they come?
68. R: Yes!
69. I: Okay, you have seen this one implemented at this clinic right?
70. R: Yes.
71. I: Okay, how is the turnup with this strategy?
72. R: The turn up seems to be good. People go for circumcision and then they come to say ‘they told me to come and collect transport’ and we give them. Just from looking at others, you can tell that they were told about it on that same day, they went for circumcision that same day and they have come to the clinic on that same day to get transport.
73. I: [Laughs] okay, are there any challenges you can think of with this strategy?
74. R: Yes, the challenges are there. The challenges are that there are some people who are giving the blue cards which they get from the health center to their friends telling them to come to the clinic for reimbursement even before they have gone through VMMC. So, we have realized that now and I would think that there are a lot of people we have attended to who just got the card from their friend. Most times, they come straight to the nurse’s office without passing through Dumbo’s office, they just tell each other which office to go. And because Dumbo is not be around at times, we just take the reimbursement and we give them. but, we have caught tw people who simply gave each other the cards.
75. I: Okay, and what has been done to deal with that challenge or what can be done?
76. R: We have not discussed what we should do there. However, if Dumbo is not around, there is a book where those peoples names are written and he has showed us that book because he writes the names of everyone he has referred in that book and so we go and crosscheck.
77. I: Okay, any other challenge with this strategy?
78. R: The reimbursement strategy?
79. I: Yes, apart from the one where they are just giving each other the cards even before they are circumcised, is there any other challenge you have experienced or you can think of?
80. R: Aa no, that is the only challenge right now.
81. I: Alright, and so eventually, we would like to implement all these at once. Am not sure, are they all being implemented right now or still one at a time?
82. R: I think its still one at a time.
83. I: Okay, so eventually, all the strategies will be implemented at once. That is the education, the SMS and the reimbursement will be implemented at once. What are your thoughts on implementing all these strategies at once?
84. R: It would be good because if one is not interested in one of the strategies, they would be interested in the other strategy and another would be interested in the other ones. People are different, some would opt to be reminded by phone while others would refuse to take the money saying that ‘I don’t want that money, wehere did you take it?’ because of the misconceptions that are there in our midst. So, all those strategies are good because if someone was not interested I one strategy, they would be interested in the other one.
85. I: Okay, first on the misconceptions, how do you think the community or culturally or religiously, how do you think people would react to hearing that there is transport being reimbursed?
86. R: For the money, some say that ‘where did the money come from’, some say that ‘it means they are buying our foreskin. After they cut our skin off, they are paying for it with money.’ There are different misconceptions, some would say ‘they are satanic, why would they give me money and yet am the one who is getting circumcised?’ people don’t understand that part.
87. I: Okay, so what can we…
88. R: People react to things differently.
89. I: Okay, and so what can we do in that case?
90. R: What is needed is that if a person raises a concern, there is need for counselling and talking to the person such that they understand why we are doing what we are doing. That the aim is to promote VMMC.
91. I: So, counselling?
92. R: Yes.
93. I: And we will only counsel the individuals who have raised a concern?
94. R: Yes, where we see a problem, we provide counselling. Even while giving the health talk to the group in the waiting area, it is good to address issues and we encourage them to speak because if they disagree amongst themselves, it is better than for a health worker to do that. When they discuss it amongst themselves, they easily believe each other unlike if a health worker did that.
95. I: Okay, bringing it up in the group discussions as well?
96. R: Yes! When we see a new problem, we need to discuss that in a group as well so that they should help each other.
97. I: Okay, and on combining these strategies, do you think the numbers would increase? You said that people who did not like one strategy would easily like one of the other strategies. However, all in all, do you think the uptake of VMMC would increase if the strategies were combined or not?
98. R: It would icrease.
99. I: Okay, if you were to choose to combine some of these strategies, which ones would you choose?
100. R: The strategies are SMS, reimbursement and what else?
101. I: Intensified education.
102. R: I would combine education and transport reimbursement.
103. I: Why?
104. R: Because the reimbursement one is the one I think most people would accept. For the SMS one, a few people have phones.
105. I: Okay, and the education?
106. R: The education is increasing awareness. If you did not know something, now you know it and you can do it. It is possible you heard it from friends who do not have enough information, but now, you have all the information and that would increase the turnup for VMMC.
107. I: Okay, and how do you think these strategies line up with what already happens at the clinic?
108. R: I feel like this is helping unlike the way it was before.
109. I: Okay, and so incorporating them into the daily routine of the STI clinic, do you think that would be hard or easy to do?
110. R: Umm, it is easy to do because the one who give the education is a different person and the one who does the other things is a different person and so it is easy to do.
111. I: Okay, and so it is easy because different people are doing different things?
112. R: Yes, it is unlike where the same person has to give the education and the other thigs as well, things do not work because the person has so much to do. however, if there are enough staff, this education strategy is good.
113. I: Okay, so considering the way things are right now in terms of staffing, do you think these will work?
114. R: Yes, they work.
115. I: Okay, alright. And if these were part of what happens at this clinic, right now it is being done as part of a study right?
116. R: Yes.
117. I: And if these were done as the normal work of the STI, what are your thoughts?
118. R: If these were put in place, they are good because they are promoting VMMC.
119. I: Okay, and in terms of how effective they would be implemented, not as a study but as part of routine STI activities, do you think they will effectively be implemented?
120. R: Yes, so long as there is enough staff.
121. I: Okay, for education, you said it would work, but let us look at the SMS and the reimbursement strategy, do you think these would work?
122. R: They would work. Actually, they can work.
123. I: Okay, with what is already there right now?
124. R: Yes!
125. I: Why is that?
126. R: Because people are welcoming them. with what I already said concerning the reimbursement, people are in poverty. As you already know the state of our country, the money helps them in other ways and not only for transport. The people do not just focus on the transport, they think of what they can do with the money. It is also possible that there are other who would want to get circumcised as I said…
127. I: But because if transport.
128. R: Yes, because of transport. They think that if I go for circumcision, what will I eat as I wait for the wound to heal, and so they opt not to do the circumcision.
129. I: Okay, and that is on the part of the people; that it helps them.
130. R: Yes.
131. I: But, on the part of the clinic, do you think it would be able to sustain this after the study?
132. R: Yes, it can continue.
133. I: Okay, and so the resources for the SMS or reimbursement, where would these come from?
134. R: I thought the study would provide the money.
135. I: [Chuckles] but studies come to an end.
136. R: Iii, then they will also stop coming when they hear that ‘they stopped reimbursing transport’. They will say that ‘I will go there another time, let me prepare’.
137. I: Okay, and so if the study pulls out, the uptake will go down.
138. R: Yes.
139. I: Okay, so what can be done for the uptake to continue being high since this is just being tried out?
140. R: Mm, I also don’t know where the transport money would come from for continuity of the activities. Because definitely, when they realize that transport is not being given, they will also stop going for circumcision.
141. I: Okay, of the three, which one do you think would be able to sustain itself?
142. R: Then it would be the education.
143. I: Just that one?
144. R: Yes.
145. I: Okay, is there anything you can suggest?
146. R: [Chuckles] I think just to increase awareness so that people know the benefits of being circumcised. That would help increase uptake of VMMC.
147. I: Regardless whether there is the transport or not?
148. R: Yes.
149. I: Alright, is there anything else you would like to share with me? Anything concerning VMMC?
150. R: [Laughs] there is nothing else but as of now, things are going fine.
151. I: Alright, do you have any questions for me?
152. R: No, I don’t have.
153. I: Alright, this is also the end of the questions I had. Thank you very much for your time.
154. R: Alright, thank you.

THE END
