# Supplementary material for: Acceptability, feasibility and appropriateness of intensified health education, SMS/phone tracing and transport reimbursement for uptake of voluntary medical male circumcision in a sexually transmitted infections clinic in Malawi: A mixed methods study
Source: PLoS One. 2025 Jan 24;20(1):e0301952. doi: 10.1371/journal.pone.0301952 (PMC11760565; doi:10.1371/journal.pone.0301952)
Supplement: S1 Data — (ZIP) [file pone.0301952.s004.zip › Qualitative data/Endline IDI Transcripts/Transcript 17.docx]

1. I: Please remember that this interview is a follow up to another that was done when this study started. It is possible that you too part or that you did not take part in the initial interviews. But still the intention of this interview is to hear your thoughts on how this study is going. Please tell me about your role at this clinic.
2. P: I work in the STI department and I register /document various sexually transmitted diseases that people present.
3. I: What are you working as?
4. P: Nurse provider in the STI
5. I: How long have you worked here?
6. P: More than 10 years but here in the STI clinic for just one year
7. I: One year?
8. P: Yes
9. I: How do you think male and female clients can be free to discuss or talk about circumcision?
10. P: On their own?
11. I: It can be discussing with you or other own or here at the clinic?
12. P: If the men are the ones who need the circumcision, they ask about how to go about it, but as for the women, I have never heard them ask about it.
13. I: Alright. But do you think they are free or how free/open can they be to talk about this?
14. P: The men
15. I: Mmmh
16. P: They are the ones who access the VMMC and also depending on how well they have understood the health education, they then have interest to ask more about VMMC,
17. I: What do you think the men at this clinic can do after telling them or talking about VMMC?
18. P: They are now supposed to go ask the right person on what they are supposed to do and which clinic they can go to access the VMMC.
19. I: Meaning that it does not happen here?
20. P: It is not done here at Bwaila
21. I: How free or open are you to talk about VMMC?
22. P: To the men?
23. I: Yes
24. P: I am free/open as long as I have all the VMMC information. We tell them in the room especially in the relation to the STI issue they present. We tell them the benefits of VMMC and if they need to hear more we tell them which room to go to, where they will be told more information about VMMC
25. I: To whom do they go and meet in the room you have mentioned?
26. P: There are people who were set to teach about VMMC in the same project
27. I: What enables you to be free/open?
28. P: The conditions we have here at the clinic, for those who have had VMMC and those who have not, hygienically those who had had VMMC are much better off than those who have not, so one just wants to help and enlighten the person that if they did this they would be better off hygienically so I send them to the people ( in the other people) to tell them more about how VMMC can help them
29. I: What makes you to be free/open to discuss these issues?
30. P: I am just free, I just look at how best I can help the person. I can also say that it is one part of my job, so if I am free it means the person to be helped I have to be open, if I am not open/free, the people cannot decide.
31. I: Alright. We are thinking about doing intensified health education on male circumcision here at this clinic. The intensified education will be conducted frequently in group health talks to do with male circumcision. Emphasis on this education will be on what is circumcision, the known benefits as well as misconceptions which are there. We will also allow patients to ask questions related to circumcision. We are thinking about allowing men who have done circumcision and their female partners to take part in sharing their experiences about circumcision. What are your thoughts about using intensified education as a way to enhance VMMC at this clinic?
32. P: I can say that these things already started. I think they are helping because once people here information about VMMC there, when they enter the room they are asking more questions and indicating that they would wish to do VMMC and where they can go. This is because they are beginning to get the information outside and the people who are teaching are I think those who were also circumcised, so they seem to have all the information base don how they know it. So, they give the people the information, then they (the clients) ask more inside and then they meet the educator again, so I feel that the intensified education done daily are heling to enlighten the men and when they have come with their wives they decide to hear more and to access VMMC.
33. I: How do you think this has affected the numbers of those who choose to do VMMC as compared to before the intensified education started?
34. P: I cannot talk about the numbers because VMMC is not longer done here at Bwaila but at other facilities. But I believe that maybe the numbers have increased but I cannot say the actual percentage.
35. I: Alright. We also have a plan to send phone messages in order to remind men who were given circumcision appointment. These phone messages will be written carefully, or will be written with a code in order to keep confidence. The messages will be sent out two days as well as a day before and on the appointment day for the circumcision. This is three times, two days before, a day before and on the day, just to remind them. What are your thoughts about using phone messages in order to enhance access to VMMC at this clinic?
36. P: It will help because some people forget. You can give them scheduled dates, but they forget. So, this will be a reminder to the person for them to come and access VMMC, and if they really desire to access VMMC they will indeed come, but without that they will just desire to hear about it and not do anything about it.
37. I: We are also thinking about refunding transport money to men who have accessed circumcision services, in order to help with the money that they have spent on this day. This money will be equivalent to $10 in Malawi kwacha as per guidelines of the Malawi National Health Sciences Committee. This money will be refunded through an established nurse at the STI clinic. What are your thoughts about using this strategy of refunding money in order to enhance access to VMMC at this clinic?
38. P: I think that it will help. There are some people who just need a certain incentive for them to go and do something. As they are learning they are supposed to say that they will be refunded transport. Some people feel that they may lack transport to comeback here for VMMC, so if you say you will refund transport they will be encouraged to come. It is like the GUT study where they are also refunded transport, they know that even if they go back after a week they will be refunded their transport, you find that the people still come. Others even send their wives to join the study because they refund transport. So, this will help.
39. I: Alright. Finally, we would like to put implement all these things we have discussed together, the Intensified education, transport refund as well as SMSs, to see how they can affect the number of men who choose VMMC. What are your thoughts on combining all these together?
40. P: It will be good because for those who desire VMMC if you send them a message, that will serve as a reminder, the intensified education will provide information while the transport will cause the person to come knowing they will benefit something. Of course, it is for transport but often it is not the exact amount. So, one will encourage them, one will remind them and the other will give them information. It will help to do them at once
41. I: Do you think that it would work?
42. P: Yes, it can work because of you give them the information there and then hey also get information in these rooms, they will decide to undergo circumcision because then they will be booked, you will then send them an SMS reminder, lets say on 23^rd^ July, you will send that 3 times, and then the promised transport, that will compel them to get on the bus and come
43. I: Do you thin it would be too much?
44. P: No, it is not. We already get overwhelming messages in our phones
45. I: Is there anything else that you feel we can add, if any?
46. P: Maybe if there can be female involvement, you know are quick to grasp things. Maybe they can help the men to decide to undergo VMMC, to encourage them. The women can also be the ones to carry VMMC messages to their homes.
47. I: How can we involve the women? How can we find them?
48. P: The same ones who come here at the STI, they can assist, if it was in the other departments we could also say that there should be someone to teach in the OPD, in the Under 5 clinic or in the ante natal at FHU. There are more women found in the clinic than men
49. I: Alright. How do you think these strategies relate to the activities of this clinic?
50. P: Which ones?
51. I: The three we have talked about, sending SMS’s, refunding transport as well as the intensified education.
52. P: Can you repeat the question?
53. I: How do you think these three methods we have talked about relate to the activities here at the clinic?
54. P: Let me skip that one
55. I: How do you think these methods relate to our culture and religion in Malawi?
56. P: There are some religions which agree with circumcision, even in the Bible circumcision was there, people were getting circumcised, so I do not think there is any difference. It just depends on the person, how they view circumcision, how they will benefit from it, you know how people are, they ask a lot of questions. But I do not think that there is an issue in relation to religion
57. I: What are your thoughts when this strategy of sending SMS’s, refunding transport as well as intensified education were implemented in this clinic?
58. P: [silence]
59. I: You mentioned that these started, what are your thoughts on these?
60. P: I cannot talk about the transport refund, what I know is of the intensified education on circumcision, on the money and the SMS’s I do not know. But daily we have intensified education on circumcision
61. I: What are your thoughts on that?
62. P: On how they are doing?
63. I: Yes
64. P: I have noted that more people are making sure to get VMMC information, once they make the decision to do VMMC, they are meeting the right people who are guiding them on where to go, but I cannot say whether they are really going to access the VMMC or it ends where they are getting the information, if VMMC was being done right here, that would have been another issue.
65. I: You have told me that you have observed the Intensified Education, from your perceptive what could have been done differently?
66. P: On the part where people are to access VMMC, in this area this is being done in are 25 and 30. If it was possible, I am not sure if in Biwi they also have a theatre, but I know that it is done in various places. If they could make Biwi a center for VMMC, people would not be motivated to travel from the Biwi area to o all the way to Area 25 or Area 30 to access VMMC. At least if we had a center that is closer to Bwaila.
67. I: What are your thoughts on these strategies that were put in place? I want to hear what your thoughts are on having these strategies as part of established activities at this clinic.
68. P: The intensified education and others?
69. I: Yes
70. P: The education already started, but on the SMS’s let me ask, will they be automated or there will be someone who will sending them?
71. I: I am not sure, but I think there will be a person who will be sending them.
72. P: Is the person from this clinic?
73. I: I should think so. Why do you ask?
74. P: It will depend on the person given the responsibility, if they have the time. At our clinic there are just three of us, so I do not know who will be doing that, you may find that the person is busy and has forgotten to send the messages. If it were the same people who are doing the intensified education it would be better, because there are times you find that they are free after giving the talks. We are only 3 Nurses, it can happen that we all busy and we are missing some people by failing to send the messages
75. I: These things have already started, perhaps it is that you are not aware,
76. P: Oh!
77. I: Yes, I think you have just observed the intensified education
78. P: Yes
79. I: What things would you like to be established on this?
80. P: The issue is just about giving people the right information, when they are given the right information and if they need more information they can go to the other designated rooms and if they are given appointments, in the private rooms they should be given numbers to call, like if they want to find out more about are 25 (circumcision Centre) they should be given numbers. Also, there are times when people go to the centers and they find that on those days they are not carrying out VMMC, so maybe the people giving out the intensified education should also give them contact numbers of the people responsible in the various centers so that they can ask in advance, what days they do VMMC. Unlike if the people go there and are turned back, yet given transport refunds. All things being equal, the people should be able to communicate the days they will carry out VMMC, depending on the situation.
81. I: On the three things we have talked about, the Intensified Education, the transport refund and the SMS reminders, which would you like to be fully established?
82. P: The intensified education and the SMS’s reminders are sustainable. The transport refund is now being done by the project, but once it is given to government it cannot e sustained, so it is important to be telling people in advance that the money issue may not go on always. Some people would still be expecting to be getting money, even if it goes to government. But if the SMS and intensified education were permanent that would not be a problem
83. I: Is there anything else that you wish to share with me?
84. P: No
85. I: Do you have any questions or comments?
86. P: No
87. I: Alright. Thank you so much for your time and what you have shared with me today.
88. P: Thank you

**END**
