# Supplementary material for: Acceptability, feasibility and appropriateness of intensified health education, SMS/phone tracing and transport reimbursement for uptake of voluntary medical male circumcision in a sexually transmitted infections clinic in Malawi: A mixed methods study
Source: PLoS One. 2025 Jan 24;20(1):e0301952. doi: 10.1371/journal.pone.0301952 (PMC11760565; doi:10.1371/journal.pone.0301952)
Supplement: S1 Data — (ZIP) [file pone.0301952.s004.zip › Qualitative data/Endline IDI Transcripts/Transcript 16.docx]

1. I: Ok. Please remember that that this is a follow up interview to the one that was done when the study started. You may or may not have taken part in the previous interview, but still this interview intends to hear from you about your perceptions on how the study is going. Please tell me about your role at this clinic.
2. P: I am a (withheld), I give health talk education, enter people’s information in the biometrics system as well as to prepare study files and so on
3. I: What is and so on?
4. P: Some activities just arise
5. I: Oh, ok. Let us talk about acceptability and appropriateness, ho do you think male and female patients can be free to talk about circumcision?
6. P: They can be free but t would depend on whether the person teaching them is free/open, if that is the case, the people also become open.
7. I: Why do you say that?
8. P: Because as one giving the education is not free, the people will not be free/open as well. Some are naturally open/free while others are not
9. I: When you say ‘free/open’ what do you mean?
10. P: Being able to speak what is needed, to express their thoughts clearly on the issues that are being discussed, some people need you to push them for them to speak
11. I: Oh ok, what do think the men at this clinic can do after talking about medical circumcision?
12. P: After telling them about medical circumcision, some of them would desire to get it done while others would not, it would just depend.
13. I: Why do you think so? What would cause that?
14. P: Because of some of their beliefs and also how it has been explained to them. If they have been told well and clearly, they can do it
15. I: Ok. How free are you to talk about circumcision?
16. P: I am free, I can explain clearly
17. I: Why is that?
18. P: Because I know that it is beneficial to the men…to both men and women
19. I: Apart from it being beneficial to both men and women, what causes you to be free/open as a health care worker?
20. P: [silence]
21. I: What causes or enables you to be open? You said some are very free/open and some are not. What causes you to be free/open to speak about circumcision?
22. P: Because I like talking to people, interacting with people. So, I can be free to talk about circumcision.
23. I: Alright. We are thinking about doing intensified health education on male circumcision here at this clinic. The intensified education will be conducted frequently in group health talks to do with male circumcision. Emphasis on this education will be on what is circumcision, the known benefits as well as misconceptions which are there. We will also allow patients to ask questions about circumcision. We are thinking about allowing men who have done circumcision and their female partners to take part in sharing their experiences about circumcision. What are your thoughts about using intensified education as a way to enhance VMMC at this clinic?
24. P: It will help the patient sot have enough information about circumcision and they will make a proper choice without force as they will have enough information.
25. I: Do you think that they do not have adequate information now?
26. P: Sometimes they lack people to guide them, they have some beliefs or myths from the communities, so this will help as they will have adequate information to make proper decisions
27. I: Ok. We also have a plan to send phone messages in order to remind men who were given circumcision appointment. These phone messages will be written carefully, or will be written with a code in order to keep confidence. The messages will be sent out two days as well as a day before and on the appointment day for the circumcision. What are your thoughts about using phone messages in order to enhance access to VMMC at this clinic?
28. P: I think that it is a good idea. To remind the person. It is also an easy way to ensure that the message gets the person
29. I: Why do you think so?
30. P: Because the person can be reminded easily, unlike having to physically visit the person, whereas on the phone you will just write and send and the person will see it and be reminded to come to the clinic
31. I: We are also thinking about refunding transport money to men who have accessed circumcision services, in order to help with the the money that they have spent on this day. This money will be equivalent to $10 in Malawi kwacha as per guidelines of the Malawi National Health Sciences Committee. This money will be refunded through an established nurse at the STI clinic. What are your thoughts about using this strategy of refunding money in order to enhance access to VMMC at this clinic?
32. P: I think it will help…will you give the money after circumcision?
33. I: Yes, it will be after they have accessed the VMMC service.
34. P: They will also tell their colleagues that they came to the clinic and they were refunded, in turn their colleagues will also come to the clinic for VMMC. As a result, more people will undergo VMMC because of the money and also, they will say they are not losing anything as they will be refunded, so I think that is a good strategy.
35. I: Do you think that some people do not come because they use their own money?
36. P: Yes. They source money in different way and for them to think of using their hard-earned money for transport and food, they at times just do not feel motivated to come,
37. I: Alright. Finally, we would like to put implement all these together, to see how they can affect the number of men who choose VMMC. We have talked about Intensive Education, sending messages by phone as well as transport refunds. So, we would like to put all these together to see how they can affect access to VMMC by men. What are your thoughts on combining all these together?
38. P: SMS, transport refund, intensive education?
39. I: Yes
40. P: I think it will help a lot of me to access VMMC, because when they receive intensive education they will know the truth about VMMC and then being refunded transport thereafter as well as sending reminders through messages as instead of physically tracing them will really them. Most men refuse to come for VMMC and they have to be traced in their homes, when they see the project cars coming into their communities they can ran away in fear of what other people will say, while with an SMS they will personally be reminded to come to the clinic, so that is easier.
41. I: Do you think that putting all these together will work?
42. P: Yes! A lot.
43. I: Why do you think so?
44. P: Because, putting all these together will make things simple
45. I: Do you think it would be too much?
46. P: no, that is enough
47. I: Is there anything else that you think we should add?
48. P: No. Nothing else to add
49. I: How do you think this relates to activities of this clinic?
50. P: They are related. Like for the intensified education, the education already happens, so the people will not feel like they are wasting their time, these are things that already happen, it is just that other things will be added. And on giving back transport money, this already happens
51. I: You already do?
52. P: Yes. For only those in a study, they get a stipend. So, it is something that already happens, what I see to be new is the SMS. I have never heard of it, but it is the easiest
53. I: How do you think these things relate to our culture and religion in Malawi?
54. P: In our culture, people do circumcision, so it is ok, Young people undergo circumcision. The cultural kind. In religion they also do circumcision. So, for me it is just fine to establish and talk about this
55. I: Alright. What are your thoughts when this strategy of sending SMS’s, refunding transport as well as intensified education were implemented in this clinic?
56. P: [silence]
57. I: What are your thoughts after this was established? What do you think is going well? What do you think should change?
58. P: On the transport refund…
59. I: On refunding transport, sending messages as well as intensified education, since they were established, what are your thoughts?
60. P: I think it is well, I do not see any challenges
61. I: From your perception, what could be changed?
62. P: Nothing, all is well
63. I: What are your thoughts on Intensified education, transport refund as well as sending messages being established activities here at the clinic?
64. P: It means clients would have received enough information before making the decision to undergo VMMC, I agree with all this
65. I: What would you like to be changed or established?
66. P: Established?
67. I: Yes
68. P: The Intensified Education should be fully established, not just for the study, it should be happening always.
69. I: Why is that?
70. P: We talk about so many things about VMMC, as such when we teach the person thoroughly, they will have enough information to decide to do VMMC
71. I: What other additional things would work with the services that are there?
72. P: I cannot think of anything else, all is well
73. I: is there anything else wyou wush to share with me on what w ehave discussed?
74. P: No
75. I: Do you have questions or comments?
76. P: No
77. I: Alright. Thank you so much for your time and what you have shared with me today
78. P: Thank you

**END**
