# Supplementary material for: Acceptability, feasibility and appropriateness of intensified health education, SMS/phone tracing and transport reimbursement for uptake of voluntary medical male circumcision in a sexually transmitted infections clinic in Malawi: A mixed methods study
Source: PLoS One. 2025 Jan 24;20(1):e0301952. doi: 10.1371/journal.pone.0301952 (PMC11760565; doi:10.1371/journal.pone.0301952)
Supplement: S1 Data — (ZIP) [file pone.0301952.s004.zip › Qualitative data/Endline IDI Transcripts/Transcript 11.docx]

1. I: First of all, tell me a bit about your role at this clinic.
2. R: I am a …the work that I do here is to help providing care to people who come to this clinic. This care is for Sexually Transmitted diseases. The other work that I do concerns research, HIV related research.
3. I: Okay, what type of care do you give the people who come here?
4. R: The care that we give is for different sexually transmitted diseases. I am not sure if I am supposed to mention the diseases.
5. I: Umm, you can just mention a few.
6. R: Okay, some diseases that people present with at this clinic are candida, this is for both men and women. Sores which we call Syphilis, another thing is which develop in the sexual organs. Another disease is Gonorrhea in both men and women. In short, those are some of the diseases we see at this clinic.
7. I: Okay, how open do you think men and women at this clinic would be to talk about circumcision?
8. R: About what?
9. I: About circumcision.
10. R: Mhh, how men and women would be open to talk about circumcision?
11. I: Yes.
12. R: Some are open but we see others who shy away from talking about circumcision. However, some are open, especially when they are in the clinic room. Depending on the disease they have presented with and we tell them about circumcision, because there are other diseases that can be helped with circumcision like candida for instance. For candida not to occur in men often, we can tell them concerning VMMC if they are interested so that they should not have the candida often. When you tell other men, they are interested to say ‘okay, when I am better, I will go for VMMC’.
13. I: Okay, and for those who are not open, why do you think that is the case?
14. R: [Chuckles] for the ones who are not open, it could be because they are shy and for others it could be out of fear of what they heard concerning circumcision.
15. I: As in the dangers of circumcision?
16. R: Yes.
17. I: Okay.
18. R: And some would want to ask their loved ones like their wife before they can make a decision.
19. I: Okay, so there are men who say they should ask their wives first?
20. R: Yes, there are.
21. I: You have come across them before?
22. R: Yes.
23. I: Okay, that is interesting [chuckles]
24. R: [Chuckles]
25. I: How open are you to talk about circumcision?
26. R: I am open to talk about it because of the work that we do and the benefits of circumcision. They say that you are protected from contracting HIV even though you are not 100% protected. But, it helps to prevent HIV. Because of that, I am open to talk about it because HIV is one of the difficult disease right now on earth.
27. I: Okay, and so you would be open to talk about it because of the benefits.
28. R: Yes.
29. I: Okay, we are thinking of conducting intensified health education on circumcision at this clinic. Intensified health education will regular group health education talks on circumcision. The education will focus on what circumcision is, its proven benefits and common misconceptions about circumcision. We will also allow patients to ask questions about circumcision. We propose to also involve men who have successfully undergone circumcision and their wives to come and share experiences around circumcision. What are your thoughts on using intensified education as a way of increasing uptake of VMMC?
30. R: I think that using the intensive education will help people because they will know what circumcision is, who can get circumcised, the benefits of circumcision, the challenges that one may face after circumcision and what to do if they experience such challenges.
31. I: I have noticed that the intensive education is being provided, right?
32. R: Yes.
33. I: After the education started, have you noticed any change in the patients you are working with right now.
34. R: The change is really there because before they started the education, the number of people who were coming were few. Currently, we see a lot of people coming. Some have already been circumcised and some have not, they approach us and ask us where to go and we point them to people involved with the VMMC so that they can assist them.
35. I: Okay, so you would attribute that change to the education being provided?
36. R: Yes.
37. I: You think that is the reason why there is the change?
38. R: There is change because of the education. People need to know, if they do not learn, they would not know other things concerning VMMC. However, because they are being taught, they can even tell their friends what is happening. They can tell their wives at home and if is the woman who has been taught, she can tell her husband concerning the VMMC.
39. I: Okay, and so there is change.
40. R: Yes.
41. I: Okay, is there anything you would like the education to include concerning VMMC? What else should be included concerning VMMC?
42. R: [Chuckles] [silence] the benefits of medical circumcision should be there. That is because medical circumcision is smart and the person is given medicine before they are circumcised. That is unlike the circumcision being done in the communities, there is no hygiene and I don’t believe they give pain killers before the circumcision. Even though we don’t really know, I don’t think they give such medicine. The benefits of circumcision as well should be included in the information.
43. I: Okay, we also to send SMS reminders to men who have a circumcision appointment. The SMS text will be carefully worded or coded for confidentiality. The messages will be sent three times; two days before the appointment, a day before and on the day of the circumcision appointment. What are your thoughts on SMS reminders as a way of increasing VMMC uptake at this clinic.
44. R: It is a good strategy because there are a lot of things that can make people forget. When people have gone back home from the hospital, they might forget what was discussed for different reasons. However, if they are reminded, they will have that conscious to come and have VMMC done. that means the number of people coming for VMMC will increase.
45. I: Because they are being reminded.
46. R: Yes.
47. I: Okay, and concerning the timing at which these messages are being sent, is there anything that you would change there?
48. R: Yes because the people will be able to prepare. If they had other plans, they can prioritize and attend to the other things later.
49. I: Okay, what challenges do you think would be there with this strategy?
50. R: Most people either don’t have phones or they just sold it. It could be that the phone has been damaged and others do not have electricity in their homes. You would find that the day the message is sent, the phone is of. because of that, they might miss their appointment dates since the phone was off.
51. I: Okay, and how can we resolve that challenge?
52. R: [Laughs] this one is hard to resolve.
53. I: Okay [chuckles].
54. R: It is hard to resolve this one because in terms of electricity, it is hard since you cannot just tell them to go and charge their phone at such a place. Some people do not have money to charge their phones and we cannot afford to give them money so that they charge their phones. We don’t even know if their neigbors would accept to charge the phones for them.
55. I: Alright, and so just like the intensive education, this one was also implemented. Am not sure if you were aware or if you saw the implementation.
56. R: Yes.
57. I: You noticed it happening?
58. R: Yes.
59. I: How do you think it went or how was it going?
60. R: Aa, I am not sure on this one. That is because we are not working hand in hand with the people involved with that. We have not had time to ask how they are doing it and so I am not sure.
61. I: Alright, I understand. Another thing we are thinking of is reimbursing transport on the circumcision day. The reimbursement will be an equivalent of $10 in Malawian Kwacha based on the National Health Sciences Research Ethics Committee guidelines. The reimbursement will be from a designated nurse within the STI clinic*.* What are your thoughts on this strategy?
62. R: Transport reimbursement?
63. I: Yes.
64. R: Mhh, it is quite a good strategy and it would help people because most people living here in town are either not working or they run a business which is not stable. In such cases, they would be assisted with the transport. This would make a lot of people come as well because they would tell each other to say ‘they are reimbursing transport after circumcision’. There are other people who fail to come to the clinic because they do not have transport. At the STI clinic for instance, we have people who have walked from Mtsiliza to come to the clinic. Some come from area 23 or 24 and when you ask them, they say they walked to the clinic. With circumcision, some would actually be lazy because they are not sick. If there is transport, they would have the heart to come knowing that ‘they will giving me back my transport’.
65. I: Okay, when I asked the question, you started with an ‘mhh’ before responding, what are some of the challenges with this method?
66. R: [Laughs] Maybe the government would not manage to reimburse the transport. I do not know…maybe this will be handed over to the government after the study is over. The government, I don’t think it would manage to reimburse the transport. That is just what I think
67. I: Okay, so sustenance is the first issue, right?
68. R: Yes.
69. I: What other challenge would be there with the reimbursement?
70. R: Others will only want to get circumcised because of the money. They wuld say ‘let me just go and get circumcised so that I find money’. It is the equivalent of $10 right?
71. I: Yes.
72. R: Yeah, so when they think of that, they might say ‘it is better I get circumcised and use that money for some household needs’.
73. I: Okay, and so their aim will be the money.
74. R: For some, yes.
75. I: Okay, but they will still get circumcised.
76. R: Yes.
77. I: Alright, am sure you have seen this one in practice, right?
78. R: Yes.
79. I: How was it going from what you heard or observed?
80. R: From what I heard or from what I saw partially, even though we are not hands on with it, from what we see of those who are coming to get reimbursement after VMMC are quite a lot compared to the past.
81. I: Okay, which past are we comparing to?
82. R: Before transport was being reimbursed.
83. I: Okay. The numbers are increasing but how has the procedure or process been or the flow of things when they come; how was it going?
84. R: From what we saw, when they come, they were first going into the VMMC room where they were handing in a card which is proof that they have been circumcised. There were also some questions which they were being asked, these were on a tablet, concerning the circumcision I think. When they are done there, they are escorted to the room where the nurse doing the reimbursement is in and they are reimbursed then they leave. That is how the flow is.
85. I: From your observation, was it going well or not or what can you improve from how it is going; anything that you can improve or change from how things are going.
86. R: That is hard to explain because with our busy schedules, we do not really…we only know the things in passing. We only see the person coming to our offices for reimbursement. When we ask them what is happening is when they explain to us that there are some questions being administered from the tablet. As such, it would be hard to know which area to improve because we just see the person coming after they are done with everything.
87. I: Alright. finally, we would like to try and implement all these strategies at once. At first, all the strategies were being implemented one at a time but this time around, everything will be done at once. That means when the person comes, they get intensive education then the SMS reminders are sent and then after the VMMC they get reimbursed. What are your thoughts on having all these strategies implemented at once?
88. R: It will help because when the person gets the SMS, they will have it in mind that on such a day, I need to go or circumcision. Others will come on the same VMMC day to be asked the questions and to be reimbursed then they go. When everything is being done at once, it will be helpful because it will not be long before the person is circumcised. I don’t know if I have responded right [laughs]
89. I: No, its fine [chuckles]. Why do you say it wont take long before the person is circumcised?
90. R: When the person gets the SMS, because of the reimbursement, the person will be motivated to get circumcised so that they can be reimbursed. Like I said, some will only be after the money while others will really want to get circumcised regardless the reimbursement. because they did not have transport, this will be their opportunity because they will be reimbursed.
91. I: Alright, do you think it will be too much to handle? On the part of the clinic staff, do you think this would be too much or it is manageable?
92. R: I don’t know if it will be the same people continuing with it as time goes. I do not know.
93. I: If it is the same people?
94. R: It will still be fine [chukcles] becsaue the work load will be lessened. With what we do here concerning STIs and other studies, to add this study on top of that would be too much for us. unless there is staff specific for it.
95. I: Okay, that is why you are saying it will be fine if the same people continue and that if it comes to you it would be too much?
96. R: Yes. But, I do not know if the questionnaires will continue or if they will stop after this phase.
97. I: I also do not know, but what do you think would happen if they continued?
98. R: [Chuckles] if the questionnaires continue, time would be a problem because of the work we already have, unless it will have its own staff.
99. I: Okay, if you were to choose such that you don’t have to implement everything, which one or which ones would you choose of the three; SMS, education and Reimbursement.
100. R: The education.
101. I: Okay.
102. R: Teaching the people in detail so that they understand.
103. I: Okay, so you think teaching them would work without the SMS and the reimbursement?
104. R: If there were two of them
105. I: Okay, which one should the other be?
106. R: [Chuckles] sending them the messages maybe. Sending them messages even though we know the challenges with the phones in terms of electricity or them loosing the phone or the phone being damaged.
107. I: Okay, so you think the SMS and the education would work.
108. R: Yes.
109. I: Okay, on transport you talked of sustenance, that the government might not be able to sustain it. Is there any other reason why you think it would not work?
110. R: [Chuckles] in terms of the other activities that happen at the hospital, people are not reimbursed. So, for VMMC to be the only activity giving out transport, people will start to wonder why it is the only one doing that.
111. I: Okay, and that brings me to another question. [chuckles] after the community learns that these strategies are being implemented at the clinic, how do you think they would react? Whether religious groups or cultural ones, how do you think they would react?
112. R: They might think there is bias. They might think that the people are being coaxed with money for them to be circumcised. I think that is what people in the communities would think, the cultural part of it. That is because culturally, people believe in circumcision being done in the communities. So, they might think that they are being hijacked because of the money. That people are being given money when they go to the clinic for circumcision which means that the cultural circumcision will no longer be valued.
113. I: Okay, and so what do you think can be done so that they don’t feel hijacked or so that they do not think we are stealing people from them in other words.
114. R: [Laughs] maybe they should be informed on what is happening, why it is happening and things like that.
115. I: Alright, alright. umm, I think that is all had. Do you have any question or any addition?
116. R: Mmm, there are no questions [chuckles]
117. I: Okay, anything you can add on what you think would help in terms of VMMC?
118. R: Aa, no. most of the things that would help are already there, they are already happening [chuckles]
119. I: Yes, continue.
120. R: There is nothing to add [laughs]
121. I: [Chuckles] okay, so how do you think the things that already happen at this clinic line up with the strategies we have discussed; do you think they fit in or they are completely different?
122. R: With what already happens at the STI?
123. I: Yes.
124. R: It is not different. Like I said, the diseases that the men present with like candida, after examining them, it is clear that they need to be circumcised. So, it is something we have been explaining and it would not be a new thing.
125. I: Alright, I really appreciate you for your time. Thank you very much.
126. R: Thank you.
127. I:
128. R:
129. I:
130. R:
131. I:
132. R:
133. I:
134. R:
135. I:
136. R:
137. I:
138. R:
139. I:
140. R:
141. I:
142. R:
143. I:
144. R:
145. I:
146. R:
147. I:
148. R:
149. I:
150. R:
151. I:
152. R:
153. I:
154. R:
155. I:
156. R:
