# Supplementary material for: Acceptability, feasibility and appropriateness of intensified health education, SMS/phone tracing and transport reimbursement for uptake of voluntary medical male circumcision in a sexually transmitted infections clinic in Malawi: A mixed methods study
Source: PLoS One. 2025 Jan 24;20(1):e0301952. doi: 10.1371/journal.pone.0301952 (PMC11760565; doi:10.1371/journal.pone.0301952)
Supplement: S1 Data — (ZIP) [file pone.0301952.s004.zip › Qualitative data/Baseline IDI Transcripts/Transcript 1.docx]

1. I: No questions?
2. R: Yes.
3. I: Okay, first, tell me your role at this clinic, at the STI clinic.
4. R: Okay, I am a (withheld) who assists patients who have come with sexually transmitted diseases at the STI clinic. Umm, I am also the nurse who supervises other UNC nurses, or I should say as a team leader not a supervisor.
5. I: Okay, how long have you been a team leader?
6. R:Umm, I have forgotten. But it is for more than 5 years.
7. I: Okay, and how long have you worked in the STI clinic?
8. R: I have been there for… umm, since I joined UNC.
9. I: Okay, and how many years ago was that?
10. R: 2003 up to date.
11. I: Okay, so that is 18 or 19 years.
12. R: Yeah!
13. I: Okay, so you assist patients who have come to the STI clinic and you are also a team leader for the UNC nurses, apart from that, is there anything else that you do on daily basis?
14. R: Umm, not really. My main role at the STI is that I am an STI provider. So, I am one of the nurses who asses our patients when they come, we make a diagnosis and we give the treatment and we also counsel them concerning the STI treatment and we encourage on partner treatment.
15. I: Okay, so there is a lot of contact between you and your patients.
16. R: Yes, there is.
17. I: Okay, so the first thing we want to see the acceptability and appropriateness of the strategy I talked about with male circumcision right.
18. R: Yes
19. I: How open do you think the male and female patients would be to talk about circumcision at the clinic?
20. R: For me, through my experience and from what I see when they give health talks on circumcision; I see that people are interested. So, I do not doubt that most men would accept this circumcision. Because of the health education that they are given, I think most people are open and they ask questions. That means they are interested. So, I think that currently, circumcision is acceptable to most people unlike the way it was previously. I think that is because of sensitizations and the coming in of the Voluntary Male Circumcision who have been going around in most places. At first, they were saying that circumcision was meant for other cultures. With the coming in of the sensitizations that they have been doing, the ones that Jphiego was doing, it seems many people have now accepted that circumcision is for everyone. For me, I think it is not hard.
21. I: Okay, you have mostly talked about the men and you have also said that they would be able to ask questions. Let’s say the talk is happening where both women and men are present, as it is in the waiting area. In that context, how open do you think they would be?
22. R: I think that men would not be very open in the presence of women. However, if that discussion were happening in the presence of men only, it would be easy.
23. I: Okay, why do you thin k having women there would be a problem?
24. R: I think that the women would be a barrier because some men are just shy. They are shy to talk about their private organs in front of women they do not know. So, I think that would hinder the men’s acceptability of the circumcision.
25. I: Okay, with the way the STI clinic is what would be the best way of bringing about or of talking about circumcision?
26. R: I think we can still talk to them about it. However, there should be another offer for those who are interested to go somewhere [in private] with the provider and discuss those things.
27. I: Okay, and in your case, how open are you to talk about circumcision?
28. R: [Chuckles]
29. I: [Chuckles] how open are you to talk about circumcision?
30. R: Okay, for me [silence] I personally do not have a problem talking about circumcision.
31. I: Okay, why is that so?
32. R: Because I know the benefits. I know that when one is circumcised, they can avoid other diseases and in terms of hygiene, I know that the person’s hygiene is improved after circumcision. I know the advantages of circumcision, which is why I think that it would not be hard for me to talk about this circumcision.
33. I: Okay, let us go to the women who do not come to the clinic; how open do you think they would be to talk about circumcision?
34. R: I think that when talking to women about circumcision, most of them do not seem interested and they would not be interested. That is because they take circumcision to be for men. With our culture, most women are afraid and they would not take that message to their husbands. That is because they are afraid of their partners. They are more submissive than open to explain to their husbands. This issue is also about a man’s private part and in our Malawian culture, most of our women are not open. That is what I observed in the STI clinic. They are not open to talk about this issue with their husband.
35. I: Okay, first you have said that is because of our culture and because it involves a man’s private part. However, do you think the reaction would be the same with other health issues? As in is it the issue of circumcision that is the problem or you feel it is the same with any other health issues?
36. R: I think that… I think they would bring up other health issues and talk to their husbands but most women are afraid. Most Malawian women are not open. The exception would be those who have gone a little far with their education, those are more open with their spouses. Those who did not do a lot of school are really scared of their partners. When a couple enters one of our rooms and we ask the woman to take off her clothes so that we examine her, it is a hard thing to do. In the end, because we realize that we will spend so much time before she can take off her clothes, we ask the man to step out and wait outside. Even when we tell the man to take his clothes off in front of the woman, the woman will look away as if they are not married.
37. I: Okay, so it is a general issue and not just related to circumcision?
38. R: Yes! It is a general issue.
39. I: Alright, I understand. We will start by discussing the ‘I’ in the RIT, which is intensive education. We are thinking of We are proposing to conduct intensified health education on circumcision at this clinic. This education will regular group health education talks on circumcision. The education will focus on what circumcision is, its proven benefits and the common misconceptions about circumcision that are there. We will also allow patients to ask questions about circumcision and we propose to also involve men who have successfully undergone circumcision and their wives to share experiences around circumcision. What are your thoughts on using intensified education as a way of increasing the uptake of VMMC at this clinic?
40. R: I think the education is good. It is good because it will encourage many people to come and access circumcision at this clinic. It will help to increase the uptake because we will impart knowledge concerning circumcision into them. As a result, many people will know about it; about its advantages and the things, they might experience after going through it. As such, it will be easy for them to access circumcision.
41. I: Okay, so they will know its benefits and that would make them seek it more; is there anything else that would make them come after this intensified education?
42. R: Umm, after the education… [Silence] maybe they will also help us spread the message in the communities where they live and that would help those in the communities who did not attend the health education to come as well.
43. I: Okay, what are some of the benefits of this strategy that you can think of, apart from first, they will spread the message to other people and they now have the information; what other benefits are there with this strategy?
44. R: [Silence]
45. I: Or even the problems that can come about with this strategy.
46. R: [Silence] [laughs] I have failed this one.
47. I: [Chuckles] alright, I understand. I will move on to the second strategy; the second one involves sending an SMS as a way of reminding the men of their VMMC appointment. This message will be carefully written or coded as a way of ensuring privacy. The first message will be sent two days before the appointment date, the second one will be sent a day before the circumcision and the last one will be sent on the day of the appointment. What are your thoughts on this strategy?
48. R: [Chuckles] it is a good strategy. However, I was thinking of the network since sometimes it cannot be trusted. My thoughts were will these messages really reach the people as they should? But generally, it is a good strategy because in short, I can say that the cost will be lower than having to visit the person just to remind them. So, in terms of cost, it will be lower. Apart from the cost, I think that it is a private way. Some people do not want tracing cars to reach their homes or for the people doing the tracing to go to their homes. On that one, I feel you are maintaining the confidentiality of the potential client who will come for circumcision rather than physically visiting them.
49. I: Okay, as the benefits, you have talked of cost and privacy. Before that, you spoke of network issues and that you never know if the message has reached the person or not.
50. R: Yes.
51. I: What could be done on that one?
52. R: Mmm, I do not know [chuckles]
53. I: Okay, apart from the network issues, what else do you think would be the downside of this strategy?
54. R: I do not know if all these people have phones. If all the potential clients will have phones or if they do not have phones. Some borrow their neighbors phone in order to have assess and so somehow, there will be a breach on the issue of confidentiality. Those are my thoughts there [chuckles]
55. I: Okay, and what can be done for those who do not have phones?
56. R: Those who do not have phones?
57. I: Yes, you mentioned there would be a breach of confidentiality if they used a neighbors phone, so what can we do in that case?
58. R: Umm, for those who do not have phones…I think there is no other way than physical visits; visit them physically to remind them. The number of times you contact that person would also reduce because the car goes there today and then it goes again tomorrow…maybe you would just go there two days before the appointment date to remind them.
59. I: Okay, so the SMSs were being sent three times but you think the number should be reduced because of the coming in of the physical tracing?
60. R: Yes, that is what I think.
61. I: Alright, are there any other thoughts on this strategy?
62. R: [Chuckles] no that is all.
63. I: Alright, the last strategy we are thinking of is Transport reimbursement for the men who have undergone circumcision to cover the costs they incur when coming for the circumcision and afterward. The reimbursement will be an equivalent of $10 in Malawian Kwacha based on the National Health Sciences Research Ethics Committee guidelines. The reimbursement will be from a designated nurse within the STI clinic. What are your thoughts on this strategy as a way of increasing VMMC uptake at this clinic?
64. R: [Chuckles] the strategy is good and it will help with VMMC uptake. However, I feel that currently, people are already coming for the circumcision and people already understand circumcision. Even if this transport reimbursement was not there, I think people would still come for circumcision. However, because the intent is for them to use it because they took their time and they came for circumcision, it is a good strategy for them to receive that money and they can use it to help themselves. Some have businesses and so that money would also help them fill the gaps that were there when they went for circumcision.
65. I: Okay, so it a good method, but people are already coming.
66. R:Yes.
67. I: In this case, would you advice for this strategy to be included or not?
68. R: No, it should be there, let us not be unfair to the people [chuckles]
69. I: [chuckles] okay, at some point, all these strategies will be implemented at once. What are your thoughts on implementing all these strategies at once?
70. R: I think it will help improve the number of men coming to the clinic for circumcision because other will be motivated. Most people will be motivated to come for circumcision.
71. I: What will motivate them in this case?
72. R: Because they have heard through the intensive health education and they have the knowledge. From there, they can go out and tell their friends. When they come to the clinic and they are reimbursed, all that together will help to increase the uptake.
73. I: Okay, and in terms of workload, how will that work out in the clinic after all these strategies have been put in place?
74. R: [Laughs]
75. I: How do you look at it?
76. R: Ehh, the workload will be there. The workload will be there on the staff but what else can we do, we just need to accept it.
77. I: If you were to choose from the 3 strategies, to choose which two would work better or which one is more efficient, which one/ones would you choose?
78. R: I think the education strategy is very good, I think the intensive health education is really good and that one should work with the reimbursement one.
79. I: Okay. Compared to the SMS, and the reimbursement, why do you think the intensive education is good?
80. R: Because with the intensive education, the person will be free to ask questions and in so doing, they will know more about the topic. That same person will help us dispel the rumors that are there in the community. Everything comes with its rumors and so the same people, because we sat with them and talked to them and they asked questions which they had and now they know the truth; these are the ones who would help us in the community.
81. I: Okay, then mixed with the reimbursement strategy, what makes you think it will work very well?
82. R: Umm [laughs] let us be honest; people want money for everything. I will give an example of the studies we have been conducting. In those studies, let me give an example of the iKnow study. Initially, this study was not supposed to give out stipend and at the beginning, enrolment in this study was hard. When they added in stipend, many people came to the clinic, some who did not even have any disease. They came just because there was stipend. In all honesty, let us just say that we our social-economic status which is very poor in Malawi, people go where there is money. They go because they know that they are losing nothing but they will gain something. That is why I am saying that if education and reimbursement worked together, many people would come. But, the workload is something that will be too much for us.
83. I: But you said you would still do it.
84. R: Yes, we will still do it [chuckles]
85. I: Okay, thinking of the activities that already take place in the STI clinic, how do you think fitting this strategy into that would work?
86. R: Yes, it will fit in. it will be combined into what we already do because things like health education and sensitization talks are given on daily basis. As such, things like the health education would just go into the talks that have been happening all along.
87. I: Okay, the health education on circumcision is being given even now?
88. R: Yes, it is given. Even when Jphiego was around, they had a core person who would come to the STI for that and it is still continuing. Because of that, I don’t think there is a problem.
89. I: Alright, you spoke of the issue of culture but in terms of fitting in the issue of circumcision, how would it go with the culture that is there?
90. R: I think that the cultures are losing their significance. So, I cannot say that this is what would happen with the culture that is there here in Malawi… I think it would have no effect and I do not think it would discourage people. That is because people already have knowledge on circumcision and so accepting this will not be very hard unlike the way things were previously.
91. I: Okay, and that is because they have the knowledge and culture is not really significant.
92. R: Yes!
93. I: Okay, are there any cultural or religious challenges that you can think of with these strategies?
94. R: [Chuckles] [silence] no.
95. I: There is none?
96. R: Yes, I cannot think of anything there.
97. I: Alright, is there anything that you would like to share with me?
98. R: No, I just appreciate that I have been given a chance to take part in this study. I have personally welcomed the strategies that want to be tried in order to help increase the uptake of VMMC among the men. As the STI clinic, we will try our best to help spread this message so that a lot of men can take part.
99. I: Okay, are there any questions?
100. R: Aaa, no, there are no questions.
101. I: Alright, thank you very much for your time and for what you have shared with me.
102. R: Thank you.
103. I: This is the end.
104. R: Thank you.
105. I: Thank you.

THE END
