# Supplementary material for: Acceptability, feasibility and appropriateness of intensified health education, SMS/phone tracing and transport reimbursement for uptake of voluntary medical male circumcision in a sexually transmitted infections clinic in Malawi: A mixed methods study
Source: PLoS One. 2025 Jan 24;20(1):e0301952. doi: 10.1371/journal.pone.0301952 (PMC11760565; doi:10.1371/journal.pone.0301952)
Supplement: S1 Data — (ZIP) [file pone.0301952.s004.zip › Qualitative data/Baseline IDI Transcripts/Transcript 6.docx]

1. I: Ok. Please tell me about your role at this clinic
2. R: My role as a (withheld) working at the STI clinic is to do consultations with STI clients who come with any sexual reproductive health problems. When they come here, we greet them and they tell us their problems and depending on the drugs we have, we assist them, if do not have (the drugs) we turn them back and tell them to go and buy and then come back. So briefly, it is consultation of STI clients.
3. I: Is there anything else that you do, on your day, when you come? Apart from what you have mentioned, if any?
4. R: Apart from treatment, there is also counselling, educational counselling, so when I said ‘treatment’ it is encompassing counselling and education, not just disbursing drugs. A lot of things here are related to issues of behavior, so another aspect is the one of education, about lifestyles, risky behaviors, as well as partners, to also help partners who maybe at home, or if they have multiple partners, that they also come and get treatment. We also talk about the risk of having multiple partners in relation to STIs and challenges that they have.
5. I: All right. How free do you think male and female clients can be to talk about circumcision?
6. R: [sighs]
7. I: We are now talking about acceptability and appropriateness
8. R: Mmmh. They all have an area, but I am not sure. If the women can be free to talk about circumcision, but I do not know if they can be free to talk about this with their partners. For the men, of course they can be free here maybe with intensive education. I say this because I have experience of two places about circumcision. I am coming from the Southern area, I have worked in Zomba and Chiradzulo, and now I have come to the central region. This is my first time to work in the Central Region. Exclusive of the time, I was in school. Therefore, I can differentiate circumcision uptake, in the two months that I have worked here. A lot of people there have undergone circumcision
9. I: Where?
10. R: In the Southern area, so I was thinking that because of the culture of the people in the South, they do that. In the two months that I have been here, for the men to accept this…of the 300 men I may have seen, the ones who have done circumcision cannot even reach 10! So, most people I am seeing; for instance today, I have seen close to 30 people, but I did not see a circumcised man. Maybe with intensive education, because maybe those in the central region think that circumcision for the Yao’s and the likes, so maybe with education they can be accept. It would depend on who is doing the counselling. I cannot say how each would accept to talk about it, but maybe with the intensive education and counseling about the benefits of circumcision the men could accept. I am not sure if the women would be able to accept it for the husbands. I am not sure if I have responded well.
11. I: Yes, but I would like to know, you also do education counselling here
12. R: Yes
13. I: Health Talks, from your perspective, how free are the men and women here?
14. R: They are free when we speak one on one, but on the group counselling there has never been even one person who has asked me a question, even if you asked, on any matter about STIs. No one who comes here wants it to be known that they are the ones who have a ‘problem’, you know about the issues of STIs, where everyone will shift blame and say, ‘it is my husband or my wife’. As such, the people will not speak as we teach. But when they come in here, they would open and confess to be suffering from some things we talked about or that they do some of the things talked about such as behavior issues, but in the group, no one is courageous enough to speak in a group, but on one to one they are free. Even in Zomba where I was and have noted that there was acceptability, I never saw a person who was free to speak up in a group, but on one to one. People can even freely tell you that they have 8 partners!
15. I: All right. You have touched on my follow up question, what do you think men at this clinic can do after you have talked about clinic-based circumcision?
16. R: After educating them thoroughly, I believe they can accept. For now we just have group education, men and women all come together, but if were to separate the men and women, I am believing that they would be free
17. I: Is that opportunity not there now? Of separating them?
18. R: I do not do that. There is another person who does VMMC counselling, I am not sure if he does that, but I think after he has done the talk they meet him in the office, where they become free. But for now we just do education talks together. But if there was an opportunity to separate them after the general counselling and have intensive education and be thorough so that people do not relate circumcision with culture, I believe that most men would accept it. I never expected that I would find many men in Lilongwe who have not undergone circumcision, yet there was a VMMC clinic, no! I never believed it.
19. I: How free are you to talk about circumcision?
20. R: I am 100% free!
21. I: What makes you to be so free?
22. R: As a health worker, knowing how important circumcision is in one’s life, with the way things are, I can do everything to help a patient. As I help the man, I am also helping his family, then their lives will be well. There are some other STI conditions that are common in uncircumcised men. Once they circumcised they are preventing such. We know about HIV/AIDS, that circumcision provides 66% prevention for that and also of other STIs that can be prevented by the same. So, for me with that as a Nurse and a health worker I can always be open for them to know the truth and make a decision to do circumcision and be helped, as they are helped it means I am also helping Malawi. So, I am free
23. I: All right. We are thinking of doing intensive health education at this clinic about male circumcision. The intensive education will happen frequently in-group health talks and will focus on ‘what is circumcision’, ‘known benefits’ as well as ‘misconceptions that are there’. We will also allow patients to ask questions about circumcision. We are thinking about allowing men who have previously undergone circumcision and their female partners to take part in sharing their experiences about circumcision. What are your thoughts about the intensive health education as way of enhancing VMMC at this clinic?
24. R: My thoughts are, I would agree or concur with whatever package that says that men should be educated thoroughly. This is because as I already said earlier, I believe that if these people were well educated…they just hear from the radio or from friends as they talk, but they have never accepted it, as currently this was known and associated with ‘Jando’ and believe that this circumcision is for those in the Southern region. Therefore, for me, I agree with that, if that was done, I think uptake would increase and we can then prevent some things through this VMMC
25. I: So we have talked about intensified education, we also mentioned bringing men who have undergone circumcision and their partners, how do you look at that?
26. R: That is a good strategy; because those are the ones who can explain to the people, as what we call ‘Expert Clients’ in ART, as people who have gone through the process, this is because people have many questions. Some say one does not have the same good sexual experiences ( after circumcision), all those things as health workers we may just present theory which is different from the practical. Therefore, if this person underwent circumcision, he knows the benefits, his experiences. So both he and his wife can speak about and respond to all questions, and perhaps clarify that it does not affect sexual relations, while the health workers talk their part, I think that all these can work out.
27. I: All right. We also have plans to send messages through the phone in order to remind men who were given appointments for circumcision. The phone messages will be written carefully, or in a secret code, so as to keep confidence. The messages shall be sent two days prior to the appointment date, a day before the appointment and on the day of the appointment. What are your thoughts about sending phone messages in wanting to enhance VMMC uptake at this clinic?
28. R: The strategy of sending phone messages is very good because firstly, we are very busy people and can at times forget. We forget Doctor’s appointments or other things in our lives, so if we are getting reminders on the phone, that is a good thing. But the challenge for this could be, for those who do not have phones or for those in the villages/rural set up where there is no electricity, where there phones could be off at the time the reminders are being sent. I do not know if those things were looked into. But all things being in order, it is a good initiative, I do not think that there is any other way on how we can reach the people remind them, the phone strategy is the cheapest.
29. I: I wanted to ask if you have any thoughts of any other possible methods
30. R: [laughing]
31. I: All right. We have talked about sending confidential messages, how do you look at that?
32. R: I would ask, what you mean but this confidential message
33. I: it is a message where if the owner saw, he would know what it is about, but another person cannot know.
34. R: All right. That is also a good method. It is like the issue of one’s status. Some people would not be happy for others to know. Many people, in the homes, can touch phones, we have kids who access phones and for them to see a message for their father that reads, “we are reminding you to come for circumcision”…, or perhaps he had taken the phone elsewhere for charging. Therefore, if it is a confidential message that no one can know, that is a good thing, the owner would not be offended that others have seen his confidential message Just as we would like to keep confidence of some other things in our lives.
35. I: We are also thinking of giving transport reimbursement to the men who have undergone VMMC, in order to refund the money they have spent on this day. This money will be in Malawi Kwacha but equivalent to $10 US following the National Health Sciences Research Ethics Committee guidelines. This reimbursement will be given through a designated Nurse in the STI clinic. What are your thoughts about this?
36. R: This is a good strategy, because some people walk to come and access VMMC services here, coming from far. This would encourage people, as they would not have transport issues. A person can courageously walk to the clinic because they are not feeling well to get to the clinic because they need help by all means, but where they are not sick and are just coming for VMMC, one can choose not to come if they are not sick and do not have transport money. So this strategy is good because we will reach the people and help them. But the issue is after the study is over and we want to continue. Sustainability becomes a challenge. Most things in the district move well but in the end sustainability becomes a problem. I do not think that government would be giving transport until the required population has been reached and circumcised. It is a good thing and you may find that someone goes back to the community and encourages others to come with the mindset that they will get transport re imbursement only to be told that the study ended and they will walk. What else can we do? But it is a good thing.
37. I: All right. As we are winding up, we would like to try to implement all things we have talked about, we have talked about intensive education, phone message reminders as well as transport refunds. We want to do all these together in order to enhance VMMC uptake for the men who have chosen to do clinic-based circumcision. What are your thoughts on using all these strategies together?
38. R: As in? My thoughts are…
39. I: What are your thoughts on doing all the above things we have talked about, at once?
40. R: All these work. It will be as if we are doing them all together and at the end of the day, the person would get the service. I do not see anything that would lead to failure. Only if all things are in place; that we have enough staff, I say this because at times you can find situations where the clients have come and there is no one to teach them, or they come and have to turn back due to challenges in service provision. But if we are organized so that all those who are responsible are organized, those giving intensive health talks, the ‘Expert Clients’, phone messages are sent on time, as well as those giving transport refunds are all available. If everything is in place, I think that this is something that is doable.
41. I: Is there any other method or strategy that you think would be helpful if it was added to the above things we have talked about?
42. R: No
43. I: You said that, “it is possible” if all things were in place. I just wanted to know what is the status now, from your experience? Some of the things you have talked about could be there already.
44. R: I think what is not there is the service. The VMMC clinic moved from here. When I came here, it had already moved. Therefore, people come and go back. People have asked me when they come for treatment of STIs, (when I said that there are some conditions that are common to those who have not been circumcised) after they are treated and healed of STIs they come back and remind me that I had talked about circumcision. Only to be told that the site is now in area 25 on Mondays and another day it is at Kabudula, what is that? I do not know what happened for VMMC to move from here, I was not here. I have no idea. That is a challenge. That (study) can come, but where will people go for circumcision? A person has come from area 36, and then you tell him to go to area 25. Is that ok? If the site was to open again and VMMC is done, the educators here have been trained for VMMC, you should not expect that because I am a Nurse here then I can do VMMC, I have not been trained! Who is going to do that? The people have to really know these things, the educators, the Expert Clients have to be found, the phones should be place, if all these things are in place. It is possible.
45. I: You mentioned that since you came from the South to here, you have noted that perhaps out of 300 people maybe only 10 have been circumcised
46. R: Yeah, it can be 10 but not more than 50
47. I: So my question is have these few people who have done VMMC done it here or the other places you have mentioned?
48. R: No! I do not know. In STI we review and note when one has been circumcised, even when registering we are supposed to ask if they have done it or not, even in the UNC database it indicates whether one has undergone VMMC or not, so this is the only time I am able to access this information not that they came for referral. When they come we refer them to the guy who does VMMC counselling, but here have never seen someone who we have referred, has been circumcised and comes back, no
49. I: I was asking this because you said the clinic was relocated…
50. R: That VMMC relocated I know…
51. I: Yes, my question now is, perhaps the uptake has decreased because the clinic is no longer there?
52. R: I am not sure, maybe it is
53. I: How do you think the above strategies relate to the activities of this clinic?
54. R: They can relate a lot, especially here at STI. As I said that if I am treating someone with an STI problem, another part for that to be solved, the solution is circumcision. Therefore, it means that there is a relationship, if one has been circumcised through these strategies, it will also ease other cases here, we can save drugs, reduce cases and many other things.
55. I: You touched on this next issue earlier, how do you think these strategies relate to our culture or our religious beliefs here in Malawi
56. R: On religion, it is related because VMMC came in Malawi after we already knew about the circumcision done by Muslems, the Yao’s. Some Christians have the mindset that since they are not Muslims or Yao’s, therefore they can do circumcision. It does seem like VMMC came after we already had it set in our minds that it is done by the above people groups, so on religion that is the relationship. I do not know if there was a study to assess the uptake between Christians and Muslims.
57. I: How about on culture?
58. R: It is the same. As I said earlier, I can differentiate what is done in the Central and Southern Regions. From Zomba where I was and here it is different, we often hear people say, “we are Chewas, Nyanja’s etc and we do not do that, that is for the Yao’s and Chawa’s”. So yes, it is related to culture. Some feel that it is not their culture therefore they cannot do it, yet it is proven that it can help them in their life
59. I: Is there anything else you would like to add, related to what we have discussed? Something that we perhaps did not discuss
60. R: No
61. I: All right. Do you have questions or comments, I may not be able to respond but I can note them for others to respond
62. R: No, I think it is the same thing that I said earlier. When the studies come, they are not here to stay, as government or an arm of government; we should have strategies to ensure that these things should be there. Some things die a natural death; they were working during a study but once the study ends, things stop, because people just need data. They (studies and teams) do their things, but what about the people who give us the data? They are still there and they still need the services. The things need to carry on for the some of the people taking part in the study and their families. If we use these strategies and VMMC takes place, we have reached the desired figures, it does not mean that we no longer have men in the population, we still have growing boys and other men who may also need circumcision; will the service still be there? After we have done our PHDs or Post Docs, what about the people on the ground? I hope I am clear.
63. I: All right. That is clear. Thank you so much for your time and for what you have shared with me. This is the end of our discussion.
64. R: All right.
65. I: Thank you
