# Supplementary material for: Acceptability, feasibility and appropriateness of intensified health education, SMS/phone tracing and transport reimbursement for uptake of voluntary medical male circumcision in a sexually transmitted infections clinic in Malawi: A mixed methods study
Source: PLoS One. 2025 Jan 24;20(1):e0301952. doi: 10.1371/journal.pone.0301952 (PMC11760565; doi:10.1371/journal.pone.0301952)
Supplement: S1 Data — (ZIP) [file pone.0301952.s004.zip › Qualitative data/Baseline IDI Transcripts/Transcript 7.docx]

1. I: Please tell me more about your role at this clinic
2. R: My role at this clinic is to help people found with various STIs. They are several grounds of these STIs, I will just mention a few, Gonorrhea, Syphilis and Bombs.
3. I: What do you do daily when you come here, what activities do you perform?
4. R: All right. When I come, we first check how we are going to help our patients. We look at materials to with such as drugs, if they are all available in sufficient quantities, if we have enough injectable, because we have many of the patients who require injections. So we check for these materials. After checking if we have or do not have the materials, we begin assisting the patients. When assisting them we have our processes. Our process is that when each client comes, they sit where we give our health education talks or where we do counselling. In that place we give counselling on STIs, HIV, and circumcision and also on any study that is being carried at the clinics. Here at our STI clinic we do studies, now we are doing a syphilis study. After the heath talk, their details are entered in a computer where we want to know their address, their name and after that the receive HTC counselling, they then go for blood testing, after that they come into a room where they receive the medication relation to the problem they have presented. That is how we carry out our work when we come here at the clinic
5. I: How free do you think male and female patients can be free to discuss about circumcision?
6. R: The male and female are free about circumcision, sometimes it is the man who is requesting for circumcision and sometimes is the woman who wants the man to be circumcised depending on the challenge they are facing in their family. So the people help each other, but often it is the men who come but some do come with their wives seeking to understand about circumcision
7. I: What do you think men can do at this clinic after you talk about medical circumcision?
8. R: That information is very important here at the clinic, because when most people have listened, at the time we start the clinic, I said earlier that we have health education on circumcision, HIV, STIs and also about the study that is taking place here. When some here the information in the group and when they are in the room getting assistance, they ask about what they heard in the group and how it goes, so we are able to help them by leading them to a person who does circumcision stuff so that they make contact. We have a phone number of the person with whom they can link up and schedule an appointment for the circumcision and be assisted. So it happens that in the group some may feel shy and not be able to speak but when they get in the room where they get their prescription, they are able to speak and we help them by leading them to the person or by giving them the persons contact number.
9. I: All right. How free are you to speak about circumcision?
10. R: On circumcision, I am open. When a person asks me, I am well able to explain clearly about circumcision. I am able to explain that it helps to prevent by 60% the chance of contracting HIV or STIs, but we need to tell the person that this is not 100%. If we misinform the person… I noted in the past at a certain time, many people were circumcised but because they did not get the correct information, many of them came with Gonorrhea. However, after counselling, after I spoke to them and said yes indeed circumcision protects but on top of that, we need to use condoms all the time if we are not having sexual relations with our spouse or the only one person. So, if you explain clearly, it happens that the people prevent. After I explained that, it happened that the number of circumcised people who were returning with gonorrhea reduced because they understood that circumcision does not offer 100% protection. So when we give health education on circumcision we need to tell the people the truth about it because if we hide something, it bring challenges later on. So we explain clearly to the people.
11. I: Some people are not free to talk about all the things you have mentioned, what makes you to be free to do so?
12. R: Indeed, it is true that some people cannot be free, but I have done this work for a while, I know how to interact with the people for them to be free. For a person to be open, you have to be the first to be open, if you are not open/free, maybe you are shouting, or you have to the clinic and there are no drugs and you are frustrated, you cannot be free with the people. So the first thing I noted for people to be free, is that you should first be the one to be free/open. How are you going to be free/open? You need to first greet them, introduce yourself. This makes the people to be free to someone who has made an effort to greet them and they become free to explain their issues. Most of those who explain do not need prompting to start asking about male circumcision they themselves being to open up once they see that you are free/open with them. So the most important thing here at the clinic is for the worker to be free, you should not be arrogant or be shouting. When you shout, you find that the person already has a problem and your attitude makes them not to disclose their problem because you have already disappointed them. They no longer have trust that you can help them. So, there is need to speak to them calmly and they understand and even being to open up, at times even to the extent of telling you their family issues they are facing with their spouses, what caused them to go and end up contracting the STIs. So if you are free with them they also open to you and tell you anything. The big issue here is for you to be free/open and they also do the same
13. I: All right. We are thinking of doing intensive health education at this clinic about male circumcision. The intensive education will happen frequently in-group health talks and will focus on ‘what is circumcision’, ‘known benefits’ as well as ‘misconceptions that are there’. We will also allow patients to ask questions about circumcision. We are thinking about allowing men who have previously undergone circumcision and their female partners to take part in sharing their experiences about circumcision. What are your thoughts about the intensive health education as way of enhancing VMMC at this clinic?
14. R: For me, I think these health teachings will be effective; most people are not able to understand what circumcision is. So these talks will help others who did not want to do circumcision to go ahead and do it because they have understood what circumcision is, what its benefits are. And bringing the spouses of those who did circumcision helps because it makes others to be motivated by what the spouse had said about what her spouse did and how things are. So I feel that all those pans you are thinking of are helpful and can lead others who have not done so, to do it.
15. I: Ok. We also have plans to send messages through the phone in order to remind men who were given appointments for circumcision. The phone messages will be written carefully, or in a secret code, to keep confidence. The messages shall be sent two days prior to the appointment date, a day before the appointment and on the day of the appointment. What are your thoughts about sending phone messages in wanting to enhance VMMC uptake at this clinic?
16. R: My thoughts on sending messages though the phone are that this is important, some people are so busy and can forget, they can set a date and find that when the day is near, they are far away or are busy and forget when the day arrives. So the phone messages will help the people to remember two days prior and even on the same day, they will be reminded to come for circumcision. Moreover, sending messages two days prior is very good as it helps the person to plan what to do the day before so that the following day they go for the circumcision. Therefore, that plan is good and important.
17. I: Do you think there can be any challenge in using this method?
18. R: No, I do not see any challenge.
19. I: Ok. We are also thinking of giving transport reimbursement to the men who have undergone VMMC, in order to refund the money they have spent on this day. This money will be in Malawi Kwacha but equivalent to $10 US following the National Health Sciences Research Ethics Committee guidelines. This reimbursement will be given through a designated Nurse in the STI clinic. What are your thoughts about this?
20. R: My thoughts are that this strategy is helpful because if a person did not have transport money but was told that he would be refunded, it will be like has travelled freely, that will encourage him to come because if he really wants the circumcision but has not transport money, it is difficult for the person to come. But if they are being given money they will not have an excuse to say, “I cannot come because I do not have money”. This strategy is also good because it will attract many people to come for circumcision because they will know that they will be refunded their transport money.
21. I: All right. As we are winding up, we would like to try to implement all things we have talked about, we have talked about intensive education, phone message reminders as well as transport refunds. We want to do all these together in order to enhance VMMC uptake for the men who have chosen to do clinic-based circumcision. What are your thoughts on using all these strategies together?
22. R: My thoughts are that the strategies that have been chosen are good, especially the health education one. This is because many people have misconceptions, they lie to one another. Some say once, “once you circumcise, you will never run again” meaning that you will no longer be sexually active. When there are those who have previously circumcised or if their spouses have come, they can testify that all id well in their family after circumcision. So this strategy will help others to do circumcision. The method of discussing through education is one way that can help many people to do circumcision.

The strategy of giving money is also helpful because those people do not have transport, they will use that and if they have change they can get some refreshment.

The other method about women is also good, if you can find women whose husbands were circumcised, and put them in groups where they can campaign about this and explain what happens and give them opportunity during the health talks to speak, I think that would be helpful.

1. I: Do you think that perhaps these strategies are too many?
2. R: No, they are not
3. I: Do you think they can work?
4. R: They can work really well
5. I: Do you think there is another strategy we can include, apart from what we have mentioned?
6. R: Another way could be having leaflets. I do not know if you have prepared posters or leaflets about circumcision, because while they are learning they can be given, so that those who did not come for the health education can read this and be attracted to come for circumcision. This can also help because messages go out in different ways. This would be beneficial
7. I: Ok. How do you think these strategies relate to the activities of this clinic?
8. R: They relate because our clinic we see people with STIs, and circumcision is one way that helps to prevent STIs. So since all these are similar, it is helpful, also here at our clinic we give different counselling, so the intensified education will just be an addition to the counselling we already give. So all that is related.
9. I: Ok. How do you think these strategies relate to our culture or our religious beliefs here in Malawi
10. R: These methods and our religion in various areas differ, that is why I said the issue of education can help us a lot, so that we leave the thoughts that we usually have. Because in the Central region, most people think circumcision is for Moslems, those who go for cultural circumcision ceremonies and that they cannot do circumcision as they pray. But the issue of circumcision is not an issue of culture or religion. It is an issue of health, and what the benefits of circumcision are. That is why I was insisting on the education. The education would help to enlighten the people, for them to know between culture, religion and the importance of circumcision. So that they do not confuse religion and the importance of circumcision.
11. I: Ok. Is there anything else you would like to share with me, related to what we have discussed? Something that we perhaps did not discuss
12. R: Maybe what we did not discuss is that here at Bwaila in the past, we used to have a place for circumcision, now they no longer do it, so many people who are told go to other sites to get the service. What I wanted to know is if it is possible, when the circumcision starts, if we can have a place here at Bwaila where circumcision can be done, this is because when people ask us for this service and we tell them to come another day, they become discouraged. In the past when the service was near, we would just give them a referral form and they would go directly and some would get circumcised same day, those who were sick and needed to heal first, were scheduled for another day. So, when they came there was no challenge. They could get it done right here. But now when they come, they have to leave this place and go to another place where they are doing circumcision. The biggest challenge is a place for circumcision, if a place was found right here at Bwaila, that would help. That would help to increase the number of people needing circumcision because the place would be centralized
13. I: You said the place was there.
14. R: Yes, it was there
15. I: What happened?
16. R: What happened was that, as far as I know is that, due to Covid 19, the place was set as a place to assistance for the corona virus. Since the Covid team left, the Eye department as well as the Orthopedics team now use the place. This means that the circumcision people will not use it again. I do not know what arrangements are there, but now circumcision is not done at Bwaila
17. I: Ok. Do you have questions or comments?
18. R: No. my comment is the same one about the place for doing circumcision. Otherwise I do not have questions and that was my last comment
19. I: Ok. Thank you so much for your time and for what you have shared with me today.
20. R: Thank you

**END**
