# Supplementary material for: Acceptability, feasibility and appropriateness of intensified health education, SMS/phone tracing and transport reimbursement for uptake of voluntary medical male circumcision in a sexually transmitted infections clinic in Malawi: A mixed methods study
Source: PLoS One. 2025 Jan 24;20(1):e0301952. doi: 10.1371/journal.pone.0301952 (PMC11760565; doi:10.1371/journal.pone.0301952)
Supplement: S1 Data — (ZIP) [file pone.0301952.s004.zip › Qualitative data/Baseline IDI Transcripts/Transcript 4.docx]

1. I: Please tell me about your role at this clinic. You may also tell me about what you do every day.
2. P: Ok. My role to provide HIV counselling as well as to do blood testing. I also escort who have been found with HIV to go and initiate into the ART program. Since we are also working with UNC my other role is to refer those found with STIs to join a study. I also refer those who are willing to do circumcision, this is after I have given counselling, they join the study
3. I: All right. Is there anything else that you do?
4. P: There is a lot
5. I: A lot? Please tell me a little
6. P: Ok. My other job is to sensitize or give counsel on sexually transmitted diseases as well as HIV
7. I: How free do you think male and female participants can be to talk about circumcision?
8. P: They can be free but it depends on the place where these people can be when talking about what is happening here at the clinic. It could be to do with the illness they are suffering from or ways for prevention. The issue is having a conducive place where they can be to speak about that they are suffering from?
9. I: When you say a conducive place, what kind of place do you mean?
10. P: Ok. I mean, the way the place we give our counsel is, like here, we have a shade where we give our health talk, that place one can be free because not many people pass by the place or other people who listen in, it is just the one giving the health talk and the patients.
11. I: All right. What do you think the men at this clinic can do after you talk about medical circumcision?
12. P: From my perspective, after telling them about circumcision, they should take part in doing the circumcision.
13. I: What do you think would compel them to do that?
14. P: There are somethings that would compel one to take part. Like here in Malawi if a person comes here because they are sick and want to get help for their issue, and you share with them about circumcision, most of these people come from far, unless if there was something that would attract that person to take part
15. I: Such as?
16. P: Like for example if someone has come here and is sick and you ask them to take part in circumcision, but the person is coming from far and if you tell them that you will refund their transport, that person would be attracted to take part. Others are given refreshments after taking part, which would also attract people.
17. I: Apart from being given transport or refreshments, is there anything else that would attract the patient to take part?
18. P: Maybe giving them counselling on what the circumcision is like. Understanding the benefits of circumcision would also help one to take part.
19. I: You talked about someone who could be coming from far, what if it were someone who comes from nearby. The person has come here while sick and you have done the health education
20. P: That is what I am saying. That even if the person comes from here, as long as they are well taught on why they are doing the circumcision and the benefits that come from doing that they would take part.
21. I: How free are you to speak about circumcision?
22. P: To speak on…
23. I: To give counselling to tell someone who has come, about circumcision, how free are you?
24. P: It is my job as a counsellor; a counsellor ought to be able to speak. Just as we speak about HIV. It is also the same on circumcision. it is our job. We are supposed to do this every day. It is our duty to tell people about what circumcision is and what HIV is, these things are interconnected; we were trained on how we can give talks. When we go to school they teach, or when we go for trainings, they also teach us on what is circumcision, what happens when one is circumcised. So I am very ready, even now if I was called today or tomorrow, I would teach someone about circumcision.
25. I: Do you not ever feel like, thee are men I cannot talk to them?
26. P: No! For one to understand something, you need to clarify really well, just as you hear the name ‘Counsellor’, is it my job to do that.
27. I: All right. We are thinking of doing intensive health education at this clinic about male circumcision. The intensive education will happen frequently in-group health talks and will focus on ‘what is circumcision’, ‘known benefits’ as well as ‘misconceptions that are there’. We will also allow patients to ask questions about circumcision. We are thinking about allowing men who have previously undergone circumcision and their female partners to take part in sharing their experiences about circumcision. What are your thoughts about using the intensive health education as way of enhancing VMMC at this clinic?
28. P: My thoughts. Ok. Another way that can enhance access to VMMC here at this clinic, is using… I do not know if we can have small pamphlets that can have messages to do with circumcision that people can read. We can also have media messages that can be transmitted through that TV set over there, currently is just plays music. Of course issues of circumcision are confidential but maybe there can b e a person, like they do in the bank, just to talk about circumcision the benefits and risks of it. The risks that can be there if one does not do circumcision. Another way is that for us counselors, when meeting the patients in this private room, we could have other extra information, which we can tell that person, to do with circumcision in addition to what was talked in the open.
29. I: Ok. You have talked about the intensified education and have mentioned adding pamphlets as well as use of the TV
30. P: Yes
31. I: How do you think using all those strategies can be helpful?
32. P: That is what would make many men to take part in circumcision, as the information would be sufficient. What happen is that some people come late and are not able to listen to the health talks, but when they come in here and find the pamphlets and read, they can take part or when they go into the HTC room and also hear about the circumcision, they could also take part. This would cause many men to do circumcision.
33. I: We also have plans to send messages through the phone in order to remind men who were given appointment dates for circumcision. The phone messages will be written carefully, or in a secret code, to keep confidence. The messages shall be sent two days prior to the appointment date, a day before the appointment and on the day of the appointment. What are your thoughts about sending phone messages in wanting to enhance VMMC uptake at this clinic?
34. P: My thoughts on the phone messages will cause…I think that is a good idea. The person could have forgotten, but through these messages, that would help many men to come to the clinic for circumcision. The same they do with ARVs, they are bale to call people to remind them about their next appointments for collection of ARVs. This has may benefits, it help to improve adherence and makes things work accordingly because they are reminded. So in the same way when it comes to circumcision, it will help. If the person had forgotten to come. For example if a person was told today and a week goes by, and they are sent reminders, it will help them to come on time. To remember to come but to also come in large numbers to come for circumcision because there is a way that has been set to be reminding them.
35. I: Ok. We are also thinking of giving transport reimbursement to the men who have undergone VMMC, in order to refund the money they have spent on this day. This money will be in Malawi Kwacha but equivalent to $10, following the National Health Sciences Research Ethics Committee guidelines. This reimbursement will be given through a designated Nurse in the STI clinic. What are your thoughts about this strategy of refunding transport in order to enhance VMMC at this clinic?
36. P: Ok. This strategy is good because most people take advantage, when they are told about circumcision they say they do not have transport. This is one way that would cause the men not to refuse, they will not worry about how they will travel back home. It is a motivation for one to come here and get circumcised. Most people accept once they here that they will be refunded transport, this attracts them. But is has a disadvantage because when dome people here the message they just think they ae going to receive money, it becomes difficult to control people, as some get the wrong information and do not really know what is happening
37. I: What do they think they are being given money for?
38. P: Instead of them understanding the issue, it draw many people to come here , even those who could never have volunteered to do, just because they have heard about the transport refund.
39. I: Finally, we would like to try to implement all things we have talked about, together; we have talked about intensive education, phone message reminders as well as transport refunds. We want to do all these together in order to enhance VMMC uptake for the men who have chosen to do clinic-based circumcision. What are your thoughts on using all these strategies together?
40. P: Ok, I think that this is a good idea because, if we take our profession as HIV counsellors, and HTC providers you know HIV has been one of the most transmitted virus because most people do not know most prevention measures. So we should take circumcision as one of the prevention measures for HIV even though it is just 60%, however if we bring all these strategies together and people hear them and do circumcision, they will prevent contracting HIV. If we also look at Cancer, the circumcision would also help their wives to reduce the risk of getting cancer and for them get sick of cancer, through circumcision, and another thing is that it helps the person to prevent some STIs. So this strategy of putting all this together and enhancing circumcision is very good because people will come and do the circumcision but also that there are other benefits the patients will have.
41. I: Do you think that perhaps putting three things we have discussed is too much?
42. P: No
43. I: Do you think they would work?
44. P: It is not too much, the information should be just like that
45. I: All right. Are there other strategies which could be added, apart from what I have mentioned; refunding transport, intensive education and SMS reminders
46. P: The other information that you could add to do with circumcision. Some people use health workers, or the same UNC staff, you could move through the various communities and give information about circumcision. For people to know. Some people even go to community leaders and give health talks, they mobilize people in the community. Another way would be to run radio adverts and people who are far can also hear the messages
47. I: Have these message snot already been heard on radios and communities? I would like to understand that…
48. P: …it happens but it has now been silent.
49. I: All, right I wanted to understand there, whether you wanted there to be other different messages
50. P: No. The same messages but now that we are doing a study, but in the past people would just go and do sensitization and people would feel that they could come anytime. But a study is done once and has a desired sample size. I do not know what your sample size is, but when doing the mobilization you can add to talk about the study on circumcision so that people come to access it.
51. I: All. How do you think the three strategies I mentioned relate to the activities of this clinic? Refunding transport, intensive education and SMS reminders
52. P: They relate, firstly, when we are doing HTC, we also need many people to know their status. So when many people come for circumcision, they will also go though HTC and this will help us to find many people would could be found with HIV but do not come for testing here at the clinic. And through other studies that are happening here at the clinic, they will also benefit to join the other studies and be able to know if they have other health issues such as STIs.
53. I: How do you think these strategies relate to our culture or our religious beliefs here in Malawi?
54. P: Ok. Some churches or religions denominations believe in circumcision as you are aware, so I think this will be one for them to benefit. As people are doing the circumcision, their beliefs are being enhanced through medical circumcision.
55. I: All right. You have touched on religion, what about cultural beliefs here in Malawi?
56. P: It is just as I said above
57. I: All right. Is there anything else you would like to share with me, related to what we have discussed? Something that we perhaps did not discuss
58. P: I just wanted to know when this study will start
59. I: We will let you know in due course when we complete all of this. Is there any other question or perhaps a comment?
60. P: No
61. I: Thank you so much for your time and what you have share with me
62. P: Thank you.

**END**
