# Supplementary material for: Acceptability, feasibility and appropriateness of intensified health education, SMS/phone tracing and transport reimbursement for uptake of voluntary medical male circumcision in a sexually transmitted infections clinic in Malawi: A mixed methods study
Source: PLoS One. 2025 Jan 24;20(1):e0301952. doi: 10.1371/journal.pone.0301952 (PMC11760565; doi:10.1371/journal.pone.0301952)
Supplement: S1 Data — (ZIP) [file pone.0301952.s004.zip › Qualitative data/Baseline IDI Transcripts/Transcript 8.docx]

1. I: First, tell me about your role at this clinic.
2. R: At this clinic?
3. I: Yes, the STI clinic.
4. R: As a (withheld), when the clients come, we welcome them and we direct them where they go to first. At this clinic, it is a routine that everyone should undergo a series of actions, if I can say that. You do not go straight into the doctor’s office, they start by sitting at the waiting area and a health talk on STIs is given. We give a talk on STIs and not on other things and we can even add issues of HIV testing and counselling. From there, they go for MMI and then they go to the nurse. As a research nurse, I welcome someone who is eligible for a study and we attend to them. However, when we do not have clients, we also attend to patients by giving STI treatment. So we do counselling, consenting, we give treatment, yes. That is what I do [chuckles]
5. I: Okay, how long have you been doing these things?
6. R: Here at the STI?
7. I: Yes.
8. R: 3 years.
9. I: Okay, and that is what you do on daily basis; if there are no clients, you help with seeing patients for STI.
10. R: Yes.
11. I: Since you meet the patients that come to the STI clinic, how open do you think both male and female patients would be to talk about circumcision?
12. R: Much as we tell them that circumcision is part of prevention of HIV preventions, most people are interested. I do not know if they are only interested because they are sick, am not sure how they would react if we found them well, maybe in their workplaces, I do not know how they would respond. The ones that come here however are very open. Some would even like to have the circumcision done right away. However, I think that because they have an infection, they want a way to remove the infection and that if they did not have it, they would not be as willing. Nevertheless, when they are sick and they have come to the clinic, they are 100% willing.
13. I: Okay, have you ever been in a situation where a health talk on circumcision was being given?
14. R: Yes.
15. I: Are they open even in that group setting?
16. R: Yes, they are very open.
17. I: Is there any difference in how open the women are, compared to the men?
18. R: Yes, there is a difference. Women are fond of hiding information even when they know the thing or they want it. In a group setting, women will not be open; they will only be open when it is just you and them. Unlike the men, only a few women are open.
19. I: Why do you think that is the case?
20. R: It’s the way they were born [laughs]
21. I: [Laughs]
22. R: For some, it is because they are shy, and others just do not want to speak in a group setting and yet they know the thing. I would think it is just the nature of women.
23. I: Okay, in your case, how open are you to talk about circumcision?
24. R: How open am I?
25. I: Yes.
26. R: I am very open to talk about circumcision.
27. I: Why?
28. R: Because I look at the benefits, so I am very open to talk about it. Because of the benefits.
29. I: Okay, if there were no benefits or if you did not know the benefits.
30. R: Aa, I would not be open about it, of what use would it be. At first, we took it to be culture and if I am not a part of that culture, if I am not Yao, then I have nothing to do with it. Currently, because of my work, I know the benefits and I can be open to talk about it. If not for the benefits or anything else and I talk about it as *Jando*, then I would not.
31. I: Okay, without the benefits, it becomes *Jando* but with the benefits, it is part of your work.
32. R: Yes!
33. I: Alright, I understand.
34. R: [Chuckles]
35. I: We are thinking of conducting intensified health education on circumcision at this clinic. This Intensified health education will be in regular group health education talks on circumcision. The setting would almost be like the one when a health talk is being provided in the waiting area.
36. R: Yes.
37. I: The education will focus on what circumcision is its proven benefits and common misconceptions about circumcision. We will also allow patients to ask questions about circumcision. We propose to involve men who have successfully undergone circumcision and their wives to share experiences around circumcision.
38. R: Okay.
39. I: What are your thoughts on using intensified education as a way of increasing the uptake of VMMC at this clinic?
40. R: That education is good. That is because in a group, someone can ask a question which you were not expecting and that would help you. Some people have trouble understanding. When you explain to them, they will not have a question right away. However, because of the group setting, they can even remind you or stimulate something in you because of their question and this is unlike one on one education. Some people understand better because of the questions compared to one person just giving a lecture throughout. When you are discussing and others are asking questions, some people get enlightened and if they did not understand at the beginning, they would understand during the discussion. People have a different way of understanding things so this would help.
41. I: Okay, the clients now understand what circumcision is from the education, how would that help with the uptake of VMMC?
42. R: After they have understood, they can make decisions and they are now motivated.
43. I: Okay, apart from them being motivated, how else would it help?
44. R: The group discussion?
45. I: Yes, especially where this intensive education has been provided and they have understood. Apart from them being motivated and making a decision, what other benefit would come out of them getting this intensive education?
46. R: They would also inform their friends or neighbors the things that are happening at the clinic. In that way, many people would come for VMMC.
47. I: Just thinking of it, what percentage of the people who come here would tell others about VMMC if they heard about it?
48. R: Umm most of the people who come here are men, so maybe I would say 50%,
49. I: 50% of the men would tell others or 50% of people who have to the clinic that day?
50. R: 50% of the men. Overall, 40 or 50% of the men would go and tell other people.
51. I: Okay, when giving this intensified education, what information should be contained in it?
52. R: The VMMC?
53. I: Yes, the intensified education which will be provided as a strategy, what kind of information should it contain?
54. R: The aim.
55. I: The aim of VMMC?
56. R: Yes, the benefits as well as addressing the misconceptions.
57. I: Okay, is there anything else?
58. R: No, that is all [chuckles]
59. I: Okay, when these things are included, how will they help with the uptake?
60. R: They will help people understand. When you understand something, you can easily do it. If you do not understand it however, you just have fear of the unknown. When you have understood, you can do it and even tell others about it. It is the same with the studies we conduct, you can sit with the person in this room for 2 hours, but, once they walk out of the room and they are asked what we were talking about, they will say ‘I do not know what they were saying.’ Therefore, once you understand, you can share the information with your friends.
61. I: Alright, the second thing we are thinking of is sending SMSs as a way of reminding the men of their VMMC appointment date. This message will be well written or encrypted as a way of ensuring privacy. It will be sent three times; 2 days before the appointment date, 1 day before the appointment date and the last message will be sent on the day of the appointment. What are your thoughts on using this strategy as a way of increasing VMMC uptake at this clinic?
62. R: The strategy is very good; it is good. Men are fond of forgetting because they do not usually come to the clinic. Our setup is one where the woman is the one who goes to the hospital and things like that. If the person is not sick, for them to remember their appointment dates, with the work that they do, they would forget. Reminding them like that however is good and they would be coming. However, I do not know the percentage of men who have phones.
63. I: Okay, what can be done in terms of the percentage of men with phones, which you have brought up?
64. R: Maybe getting the phone number of a close relative or a neighbor. Aa, but in that case, the confidentiality would not be there.
65. I: It would not be there.
66. R: Yes, I do not there would be any confidentiality in sending an SMS through another person’s phone. There would be no confidentiality.
67. I: Okay.
68. R: Maybe you would buy phones for them.
69. I: The study should buy the phones.
70. R: Yes, buy phones and give them.
71. I: And that would work [chuckles]?
72. R: Yes, we have seen that happening. Buying phones for the people.
73. I: Okay, so buying phones for those who do not have them.
74. R: No, when that happens, you buy a phone for everyone whether they already have it or not.
75. I: Okay, for the cons of this strategy, the first is that some people do not have phones. What else would be a challenge with this strategy
76. R: Maybe some people do not how to read. The illiterate ones.
77. I: Okay, and what could be done for those who cannot read?
78. R: You would opt for calling them.
79. I: Instead of sending the SMS.
80. R: Yes, and that would work using the neighbors phone or a friends phone. [interview paused]
81. I: Yes, you were talking about calling.
82. R: Yes, the calling would work even if you use a friend’s phone or a neighbor’s phones unlike the SMS. You cannot send an SMS to another person’s phone, especially when it is study related.
83. I: Okay, what percentage of men would come after receiving the reminder; how much men do you think would come?
84. R: 90%.
85. I: Okay, why do you think so?
86. R: The excuse most give is that they forgot or that they had so much work to do and the like. However, most of them say they forgot, that is why I have put it at 90%.
87. I: Okay, as the last strategy, we are also thinking of reimbursing transport to men who have undergone VMMC as a way of covering the expenses on the day of circumcision. This money would be the equivalent of $10 in Malawi Kwacha as per the National Health Science Ethics Research Committee. This money will be given through a nurse stationed at the STI clinic. What are your thoughts on transport reimbursement as a way of increasing the uptake of VMMC at this clinic?
88. R: Transport reimbursement is a very good strategy. That is because some people would be lazy to come for VMMC because they do not have money and so it would be hard for them to walk back home with the wound they get after circumcision. It is hard for most people to earn money and so this would help. They would come knowing that they will not have to walk back home, there will be transportation.
89. I: Okay, apart from that, what would be the other benefit of this strategy in terms of increasing uptake?
90. R: The transport reimbursement?
91. I: Yes.
92. R: Some can use the same transport money for their home needs. The amount mentioned is more than what they spend on the transport; there is some change left. Some hardly work or have any businesses and that money would help them in other ways in their day-to-day life.
93. I: Okay, what disadvantages can you think of with this method?
94. R: The disadvantage is that it is as though we are trying to coax the people by giving them transport money, which is k7000. Some people ask to say ‘is all this transport?’ because it does not make sense. It is as if we are coercing them by giving them so much money so that they take part. That is the disadvantage.
95. I: Okay, and what kind of impact would that have on the numbers of people coming or not?
96. R: It has a positive impact because people rush the money. They forget the things that will happen there; they can forget everything and just rush for money. They look at the advantages to say I will be circumcised and they will give me this much money. It is also one way of gaining their interest.
97. I: Okay, and so the impact will be positive and not negative?
98. R: Yes, because it is for their own benefit.
99. I: Okay, at the end of it all, all these strategies will be implemented at once. What are your thoughts on implementing all these strategies at the same time?
100. R: It is good; it means the study will go well.
101. I: Explain that.
102. R: First, we are educating and afterwards the person has information. From there, we are reminding the person when they are supposed to come and then we are reimbursing their transport money. At the end of the day, the people we loose along the way are few. It is unlike just giving them information and they go and we expect them to come without knwing when when, we could lose some on the way. If we do not remind them, they will not come. When they think of cost, they would rather run their business than come to the clinic but when they think of the k7000, they will come. All that will help the study run well, they will help with the retention.
103. I: So you think the uptake would be high and the numbers of those not coming would be little.
104. R: Yes!
105. I: Okay, if you were to chose one strategy out of all of these or if you were to choose two strategies that would work better, which ones would you opt for?
106. R: Umm… the two I would choose are the first one…
107. I: Intensified education?
108. R: Yes.
109. I: Okay.
110. R: And the transport reimbursement. Telling the in advance that they will be reimbursed.
111. I: Telling them in advance [chuckles]?
112. R: Yes!
113. I: Compared to the SMS strategy, why have you chosen these two?
114. R: Because we are giving the person knowledge of what is happening. When they leave their homes, it is not like they do not know what will happen. therefore, if you have the knowledge, that with what I have chosen, this is what will happen, that is what would make someone come to the clinic. The second thing is travel expenses. Here in Malawi, when they think they will be reimbursed, they will come to the clinic.
115. I: Okay, and the SMS one?
116. R: It has so many unknowns, because not a lot of people have phones. You cannot trust a friends phone and even for the phones that are theirs, they have to take them to places where they get charged and it can happen that when you send the SMS, the phone is off.
117. I: Okay, and in terms or workload, either on the nurse’s side or on the number of activities the client has to go through, what are your thoughts.
118. R: On that, we just try to assist the people fast so that the waiting time is not long.
119. I: Okay, do you think it is doable though? For all these to be implemented and managed?
120. R: Very much.
121. I: What makes you say that?
122. R: When we come to work, we divide ourselves in groups and we cannot leave them unattended. One is doing this and another person is doing another thing. If the rooms are not enough, we tell the people to wait for us for a little bit. Because the clients actually see us working, they understand that when we are finished with one, the next person comes in. Therefore, we would work so that the waiting time is not long and the clients should feel that ‘they have told me this, and this is really what is happening. Instead of lying to them.
123. I: Okay, and so it would work?
124. R: Yes.
125. I: Okay, so it would mean bringing in these strategies into the STI clinic where there are other things already happening.
126. R: Yes.
127. I: How well do you think these would fit into what already happens in the STI clinic?
128. R: They will fit.
129. I: What makes you say that?
130. R: [Chuckles] because I am hands-on in the clinic, I know that these things will fit in.
131. I: For someone like me who is not hands-on and wants to understand what makes you confident that these would fit in; what things are you looking at.
132. R: The way we do everything here, things work out. As such, everything can be done [chuckles]
133. I: [Chuckles] because of the way you do everything
134. R: Yes, everything is possible.
135. I: Okay.
136. R: When people come and they are looking for our services, we need to provide the services. If the rooms are not enough, we work so that in the end, everyone gets what they are seeking.
137. I: Okay, you talked of culture earlier on, where you spoke of circumcision as *Jando* and now looking at circumcision as part of your job. In the same like, thinking of culture and the different religions, how well do you think these strategies align with them?
138. R: [Sighs] [chuckles] there are discussions that take place. In that, the people talk and some speak of the advantages while others speak of the disadvantages and you clear the misconceptions that are there. For those who take it as a cultural thing, through counselling, the people realize that it is part of care and not culture. Things changed now, and people understand.
139. I: Okay, and so it would not bring about problem with the different cultures and religions?
140. R: No! And, we continue to say that it is voluntary, we are not forcing anyone. From that, people also say that ‘if they are not forcing me, it means these things are good’ and they make a decision on their own from their own understanding.
141. I: Okay, is there anything else you would like to share with me?
142. R: Aaa, there is nothing.
143. I: [Chuckles] okay. Any question?
144. R: No.
145. I: Okay, this is also the end of what I had. However, I really appreciate you taking your time and everything we have discussed. Thank you.
146. R: Thanks.

THE END.
