# Supplementary material for: Acceptability, feasibility and appropriateness of intensified health education, SMS/phone tracing and transport reimbursement for uptake of voluntary medical male circumcision in a sexually transmitted infections clinic in Malawi: A mixed methods study
Source: PLoS One. 2025 Jan 24;20(1):e0301952. doi: 10.1371/journal.pone.0301952 (PMC11760565; doi:10.1371/journal.pone.0301952)
Supplement: S1 Data — (ZIP) [file pone.0301952.s004.zip › Qualitative data/Baseline IDI Transcripts/Transcript 9.docx]

1. I: First, tell me about your role at this clinic.
2. R: Okay. I test people for HIV; I also provide counselling.
3. I: Okay, is that all?
4. R: Yes, that is all.
5. I: Okay, whom do you provide this counselling to? The ones you are testing or?
6. R: The first thing that happens is group education. However, we mainly provide counselling on the benefits of getting tested for HIV, positive living and a little information that we know on STIs. From there, we start the testing and if someone tests positive, we do linkage and we explain to them how they will take their medicine.
7. I: Okay, where is the health education given?
8. R: At the waiting area.
9. I: Okay, and how long have you been doing what you have described?
10. R: For four years.
11. I: Okay, considering the health talks that you give in the waiting area, how open do you think the male and female patients would be to talk about circumcision.
12. R: They would be very open. That is because most times, the people who come to the STI clinic are in pain. As such, they are free to say whatever the doctors ask for. We believe that once a patient comes to the hospital, only a small percentage hides their diseases. Because we tell them that ‘the doctor prescribes medication based on the illness he is told’, people are open.
13. I: Okay, they would even be open to talk about circumcision?
14. R: Yes, they would be very open.
15. I: Would there be any difference in how open they are between the male and female patients or not?
16. R: Aaa [phone rings] it would be different because when they are in a group setting, the men are the ones who are open unlike the women. When it is just you and the person, how open they are also differs.
17. I: Why do you think that is the case?
18. R: It also depends on how the is [their character]. Some are shy whilst some are flexible. As such, it depends on the way ones understands something and how they can express it.
19. I: Okay and so for the men, they would be comfortable regardless it being a group setting or individually?
20. R: Yes, they would.
21. I: Okay, and how open are you to talk about circumcision?
22. R: I am very open because with the work that we do, and when we go for counselling, they tell us to be very open. You are not supposed to be shy even when mentioning the private parts. If you are shy, the patient will not open up to you and you would not give the right counselling.
23. I: Okay, because of what you were taught during counseling, you would be open to talk about circumcision.
24. R: Yes.
25. I: Alright. We are thinking of conducting intensified health education on circumcision at this clinic. Intensified health education will be in regular group health education talks on circumcision, meaning the setting will be the same as in the waiting area right?
26. R: Yes.
27. I: The education will focus on what circumcision is its proven benefits and common misconceptions about circumcision. We will also allow patients to ask questions about circumcision. We propose to involve men who have successfully undergone circumcision and their spouses perhaps, to share experiences around circumcision. What are your thoughts on using Intensive education as a way of increasing VMMC uptake at this clinic?
28. R: Okay, my thoughts are that [phone ringing]… okay, every client comes in blank; they do not have any information. So, if the intensive education will be there, it will help to civic educate the clients so that they know the benefits and the disadvantages of it. That is what would help the clients to make a decision to take part and undergo VMMC.
29. I: Okay, apart from helping them with decision-making, how else would intensive education help in increasing the uptake of VMMC?
30. R: When the civic education has taken place, they will know the benefits. On the other hand, because this is the STI clinic, the VMMC will also help them. They say that VMMC reduces the chances of contracting diseases by 60% is it, yeah, so it is a good thing.
31. I: Okay, anything else?
32. R: The other benefit is hygiene. It means the clients will benefit after they have done the VMMC. It means the hygiene will be there.
33. I: Okay, they are getting this intensive education, but they have not yet undergone VMMC. First, they get this information and they become aware of the benefits and things like that. What happens after they know this information, what change will be there?
34. R: The chances of them undergoing VMMC will be high. You have taught them, and they know what VMMC is. Because the doctors are the ones who have taught them, they can easily ignore other beliefs.
35. I: Alright, you mentioned that they will know the benefits of the VMMC through the intensive education, what other information do you think should be included.
36. R: The same things I talked about, the hygiene, that their chance of contracting HIV decreases, those.
37. I: That is all?
38. R: Yes [laughs]… that is all.
39. I: Alright, we are also thinking of sending SMSs as a way of reminding the men of their VMMC appointment date. This message will be written safely or encrypted as a way of ensuring privacy. This message will be sent two days before the appointment date, the second will be sent a day before the appointment and the last one will be on the appointment date. What are your thoughts on using the SMSs as a way of increasing uptake of VMMC at this clinic?
40. R: It is a good thing because everyone has a plan for each day. Circumcision is something you plan; you ask them when they are free. When you send the SMS reminder, that person will be able to plan to say ‘on this day, I am supposed to go to the clinic’ and they can even reschedule the other programs that they had. Unlike if you just stay and tell them on the same appointment day that they are supposed to come.
41. I: Okay, so it will help with planning.
42. R: Yes, planning.
43. I: Okay, any other advantage or how else will this strategy help apart from planning.
44. R: That will serve as a reminder. It will also help to prepare the person. They will have a wound afterward, so it would prepare their mindset.
45. I: Without preparation, do you think there would be any impact on VMMC uptake?
46. R: Mmm [laughs] I would lie [I am not sure]. I do not know if there is any connection between the wound and the person. However, I feel that what you tell your mind is what your body does. So, I think…yes.
47. I: Okay, we have talked of the benefits, what disadvantages can you think of with this SMS strategy?
48. R: Mmm, no. I do not see any problems with this strategy. As long as you talked to the person and they gave you their number or they agreed to come for VMMC and you are simply reminding him, it is up to him to say whether he will come or not. As I said, it serves as a reminder. I do not think there is any harm or any disadvantage in doing that. Unless the person refused, if you ask him if you can send a message or call them, if they refuse both and you call them that would be violating his rights.
49. I: Okay, so before sending the message, the person must agree to be contacted.
50. R: Yes, they need to be asked to say ‘can we send you and SMS? Can we call you or can we visit you?’ you should give the person the option to choose.
51. I: Okay, if we are sending messages, what impact would that have on the number of people coming for VMMC?
52. R: Because it will serve as a reminder, people will plan. As a result, the number will increase. Some people can forget. Circumcision is unlike being operated on because of a stomach problem that you have for instance. Because the problem is causing you pain, you can easily remember. However, because you are fine and you have just decided to go for VMMC, the case will be different [you may not remember].
53. I: Alright, I hear you. The third thing we are thinking of is reimbursing transport to the men who have undergone VMMC to help cover the expenses on the day of circumcision. This money will be the equivalent of $10 as per the National Health Science Ethics Committee regulations. This reimbursement will be given through a designated nurse within the STI clinic. What are your thoughts on using this strategy to increase uptake of VMMC at this clinic?
54. R: The advantage is that in Malawi [chuckles] there is that spirit where if you tell someone that this is the benefit of this thing, they still want to benefit from you. So, because you will tell them that they will be reimbursed, I think it will empower them and when they compare the time they spend at the hospital against the time it would take them to find money, this money will compensate for that time.
55. I: Okay, that is the first benefit of this strategy, what is the second one.
56. R: The second one is that people earn differently. Being a government hospital, people with different financial statuses come here. For some to come to the hospital, they have to try so hard to find transport money for that day. If you add another day for them to come for VMMC, it will take a longer period for them to find more money they can use for transport. However, once you tell them that you will refund, that will empower them to come knowing that they will be given transport.
57. I: Okay, what negatives can you think of with this strategy?
58. R: Okay, the negatives would come in because people have different thinking capacity. Some might think they are being bribed with the money for them to come for VMMC. Others might not even think that you are covering their expenses, they might think the other way round, that it is a bribe.
59. I: Okay, with that mentality, that the money is a bribe, what effect would that have on the numbers that come to the clinic?
60. R: It means the numbers would be low. However, the way Malawians are, I do not think many people might think that way.
61. I: Okay [chuckles] so only a few would think in this way.
62. R: Yes, a few would think this way.
63. I: Okay, any other negatives you can think of.
64. R: No, that is all.
65. I: Alright. Finally, we will try all these strategies at once to see how they will affect of the number of men who will choose to get medical circumcision. What are your thoughts on combining all these to increase VMMC uptake?
66. R: I think it is a good thing because once you civic educate a person, you have told them that you will reimburse the transport, I think at the end of it all, the person will not lose anything financially because of the refund. On the other hand, the person will also benefit their health in terms of hygiene, reducing the risk of contracting of HIV, so it is a good thing.
67. I: Okay, and in terms of the numbers of people coming, how would they be?
68. R: I think they would be high since you are providing everything. If you are providing VMMC and you also give them painkillers and you tell them how to care for the wound. If you provide transport when they come for checkup, the numbers will go up. Unlike telling them to come on a particular day with their money, considering peoples financial status as I said, it would not work.
69. I: Okay, if you were to choose which two strategies would work better or which one alone would work; which ones or which one would you opt for?
70. R: I would opt for educating them and reimbursing the transport. However…that one where they are getting reminders…
71. I: The messages?
72. R: Yes, the SMSs are also good because if they come on their own, they will not think of it as a bribe when the transport is reimbursed.
73. I: Explain that.
74. R: You have educated the person and they decide on their own to come for VMMC, he will come whole heatedly and not expect anything from you. For someone who comes on their own, their mentality will be that the hospital people want to help them. If we combine it with the reimbursement, they will think of the money as a bribe to get them to do VMMC.
75. I: Okay, in that case, what are you opting for?
76. R: The best of it all is combining reimbursement and the intensive education, educating the person first. My second option would be to educate the person without reimbursement.
77. I: Just so I am clear, if combining, you would choose intensive education and reimbursement but if you were to choose just one, you would opt for education.
78. R: Yes.
79. I: Okay, I understand. However, do you think combining all these strategies would work?
80. R: Yes, they would work a lot. Considering the way we are right now, everyone keeps saying money is hard to come by because of COVID. If you do that, it would work a lot better.
81. I: Okay, if these strategies were really put in place, what can you say in terms of work load on the part of the clinic; adding these strategies to the work that is already there.
82. R: Okay, the workload could be high. …when a client comes, because of the services already provided here, the workload would be high. That is because when the client comes in the morning, they sit in the waiting area and then they go in for registration. After registration, they go back to the waiting area and then we get them from there for HIV testing. If they test positive, we have to record them in our registers, I think we have four registers; one for linkage, HTS, Oraquick and AID. From there, we teach them how to take the medicine and they have to go and collect medicine for their STI. The workload would really be… a lot. However, I believe in teamwork and so we would share some responsibilities and everything would work.
83. I: It would work out.
84. R: Yes.
85. I: And for the patient, would it not be too much to go through all these strategies?
86. R: No, it would not be too much. That is because for a client who has agreed to VMMC, we favor them so that they receive every service first; to shorten the time it takes them to go through the whole process. It means you put them in front to receive the medicine, so I do not think it would be a lot since the person will be the priority for the day.
87. I: Okay, considering what already happens in the STI, how well do you think these strategies would fit into the system that is already there? Would they seem out of place? Or, they would fit into what already happens?
88. R: No, they will fit in because this is the STI clinic and some STIs come in because of lack of hygiene. Apart from that, there is a big relationship between STIs and HIV. As such, when we tell the person about VMMC and they decide to go through with it, it means the chances of them contracting the other diseases would be low. However, for you to talk about VMMC in the OPD, that would be out of place. However, being the STI clinic, it is a good thing.
89. I: Okay, so bringing in these strategies into what already happens would not be a problem?
90. R: No, it would not be.
91. I: Okay, and in terms of our culture or our different religious beliefs, how do you think these strategies would do?
92. R: They would work because… I do not know if it is the southern region were circumcision is already being done, they call it *Jando*. As such, I think it is a normal thing and people know it.
93. I: Alright, is there anything else you would like to share?
94. R: No.
95. I: [Chuckles] okay, do you have any questions?
96. R: No, I do not have any question [chuckles]
97. I: Alright, this is also the end of what I had. Thank you very much for your time.
98. R: Yes.

THE END
