# Supplementary material for: Acceptability, feasibility and appropriateness of intensified health education, SMS/phone tracing and transport reimbursement for uptake of voluntary medical male circumcision in a sexually transmitted infections clinic in Malawi: A mixed methods study
Source: PLoS One. 2025 Jan 24;20(1):e0301952. doi: 10.1371/journal.pone.0301952 (PMC11760565; doi:10.1371/journal.pone.0301952)
Supplement: S1 Data — (ZIP) [file pone.0301952.s004.zip › Qualitative data/Baseline IDI Transcripts/Transcript 5.docx]

1. I: Please tell me about your role at this clinic
2. R: I work as a (withheld)
3. I: Can you please share with me what you every day?
4. R: When I first come, I enter people’s names in the system using the biometric system here, EMR. When we finish entering the people in the system, we then give a health education to the patients who come here. We then check if we have enough folders for the study and many other things like that
5. I: All right. How free do you think male and female patients can be free to talk about medical circumcision at this clinic?
6. R: If the person giving the health talk is also free
7. I: Why do you say that?
8. R: If the person giving the health talk is free, the patients will be free but if she is shy the patients will also not be willing to be open
9. I: What would make the person giving the health talk to be free?
10. R: Knowing the reason the person is giving the messages and keeping in mind that they are speaking to different people who need someone open so that they too can be open
11. I: what do you think men here at the clinic can do after you have talked about medical circumcision?
12. R: Many can be willing to do circumcision
13. I: What would make them to be willing to do that?
14. R: Because circumcision helps to reduce chances of contracting STIs and HIV by 60%. So when they hear the health talk messages they would decide to do it and they also become hygienic.
15. I: How free are you to talk about circumcision?
16. R: I am free because I note that it helps the people to be healthier and to protect themselves from STIs and HIV by 60%
17. I: All right, what makes you to be free?
18. R: Because I would like to help other who do not have this knowledge, it is a joy for me to teach those who not know.
19. I: All right. We are thinking of doing intensive health education at this clinic about male circumcision. The intensive education will happen frequently in-group health talks and will focus on ‘what is circumcision’, ‘known benefits’ as well as ‘misconceptions that are there’. We will also allow patients to ask questions about circumcision. We are thinking about allowing men who have previously undergone circumcision and their female partners to take part in sharing their experiences about circumcision. What are your thoughts about using the intensive health education as way of enhancing VMMC at this clinic?
20. R: It is good
21. I: Why do you think it is good
22. R: Because it will help us to know what people think about circumcision and the challenges they face when they have done circumcision
23. I: What about on the part of letting men who have done circumcision and their partners to take part? What are your thoughts on this?
24. R: I think it will help those who have not done the circumcision to know the benefits.
25. I: All right. We also have plans to send messages through the phone in order to remind men who were given appointment dates for circumcision. The phone messages will be written carefully, or in a secret code, to keep confidence. The messages shall be sent two days prior to the appointment date, a day before the appointment and on the day of the appointment. What are your thoughts about sending phone messages in wanting to enhance VMMC uptake at this clinic?
26. R: You will be calling them?
27. I: Sending messages to remind them that they have two days left, then one-day left and then one that says, “Today is your appointment date at the clinic”.
28. R: I think that is good because the person will know when they have to go to the clinic. Without that reminder, they can forget. So I think it is good. These will be frequent reminders for them to come to the clinic for the circumcision
29. I: All right. We are also thinking of giving transport reimbursement to the men who have undergone VMMC, in order to refund the money they have spent on this day. This money will be in Malawi Kwacha but equivalent to $10, following the National Health Sciences Research Ethics Committee guidelines. This reimbursement will be given through a designated Nurse in the STI clinic. What are your thoughts about this strategy of refunding transport money in order to enhance VMMC at this clinic?
30. R: Refunding money to the patients is good because they will others that you refunded them transport after they had done the circumcision. Because of that, the number of people doing circumcision will increase, many will come where there is money [laughing]
31. I: Based on your past experience, how do you view this strategy, apart from the increase in numbers
32. R: I think that it is good
33. I: Why is that? Apart from increasing numbers, why else do you think it is good?
34. R: Apart from increasing numbers, the person will have the feeling that he is not wasting his money as he will be refunded. He will feel that he is not losing anything, but he is getting helped
35. I: Finally, we would like to try to implement all things we have talked about, together; we have talked about intensive education, phone message reminders as well as transport refunds. We want to do all these together in order to enhance VMMC uptake for the men who have chosen to do clinic-based circumcision. What are your thoughts on using all these strategies together?
36. R: The intensified education and medical circumcision?
37. I: Yes, intensified education, transport refund, phone reminders as well as escorting them to the clinic. What are your thoughts on putting all these strategies together?
38. R: I think that it will help the patients. Clients to feel that they are well cared for when they come here. That they are escorted from place to place, they will acquire a lot of knowledge through the intensified education, and will be refunded transport. The patient will feel compelled to come here because they are well cared for
39. I: All right. Do you think that all these will work together?
40. R: Yes, they would work together. The patients will think when they come here they are well trained, they are escorted and after circumcision they get a transport refund, so these will work well together
41. I: Do you not think that it is too much
42. R: No, it is not too much, it will go well
43. I: Are there other strategies, which we can add? If you think so.
44. R: Did you say that you would be sending them messages?
45. I: Sending reminder messages two days prior, one day prior and also on the day, when they come they will escorted, they will get intensified education and will also be refunded tier transport.
46. R: Mmmh
47. I: Apart from these strategies, do you think there are other ones that would work if we added to this?
48. R: No, this s fine as it is
49. I: How do you think these strategies relate to the activities of the clinic?
50. R: They relate because firstly, already when the clients come here we have a health talk, so when we add the circumcision issues and clarify clearly, it will work well, they will not think that we are wasting their time, this is something that already happens and we will just add extra messages. We also already escort the patient to the clinic. If we find a patient who needs to go elsewhere, they have to be escorted.
51. I: All right. How do you think the strategies relate to our culture or our beliefs here in Malawi?
52. R: The circumcision?
53. I: The strategies we have talked about, all these are about circumcision. So how do you think they relate to our culture or religion beliefs?
54. R: It relates with some beliefs but not others
55. I: Can you explain
56. R: For those who already do it, we are different people, some believe in circumcision and some do not, for those who believe and already do circumcision, it is not difficult. But for those who do not do circumcision, it is difficult, so it just depends on how those receiving the messages have understood
57. I: Do all these meet in one place when they come here?
58. R: Yes they meet and get the health talks in one place, so it depends on how you have explained the things and how the people have understood
59. I: Is there anything else you would like to share with me, related to what we have discussed? Something that we perhaps did not discuss
60. R: No, there isn’t
61. I: Do you have questions or comments?
62. R: No
63. I: Thank you so much for your time and what you have shared with me
64. R: Thank you

**END**
