# Supplementary material for: Acceptability, feasibility and appropriateness of intensified health education, SMS/phone tracing and transport reimbursement for uptake of voluntary medical male circumcision in a sexually transmitted infections clinic in Malawi: A mixed methods study
Source: PLoS One. 2025 Jan 24;20(1):e0301952. doi: 10.1371/journal.pone.0301952 (PMC11760565; doi:10.1371/journal.pone.0301952)
Supplement: S1 Data — (ZIP) [file pone.0301952.s004.zip › Qualitative data/Baseline IDI Transcripts/Transcript 2.docx]

1. I: I want us to talk about an intervention being tried out at this clinic. The intervention is called RIT and it stands for Reimbursement for transport, Intensified health education and SMS Tracing. We are trying this intervention to see if it can improve the men’s desire for medical circumcision and increase the number of men going for circumcision. All your responses are confidential and your name will not appear on this form. If you cannot, or do not want to answer a particular question, tell me and I will go on to the next one. Please answer questions honestly.
2. R: Okay.
3. I: Remember, there are no right or wrong answers to these questions we just want you to answer them as best as you can. Please feel free to respond, the questionnaire will be the same length of time regardless of the answers you give. Do you have any questions before we start?
4. R: No questions.
5. I: Okay, first tell me about your role at this hospital.
6. R: Okay, my name is [name withheld] and I am a (withheld) I provide counselling concerning HIV and I also test people for HIV. I work with Lighthouse and I am currently working in the STI clinic at Bwaila.
7. I: Okay, how long have you worked as a counsellor?
8. R: I have worked for almost 12 years.
9. I: Okay, and how long have you been in the STI clinic?
10. R: As of now, I think this is my second year there. Previously, there were other studies being conducted there and I worked for more than two years.
11. I: Okay, as a counsellor, what are some of the things you do on daily basis?
12. R: When we arrive, we dust the room that we are working in. If it is a Monday, we order test kits if they are not enough and everything else we might need. We also provide health talks concerning HIV testing and HIV self-tests.
13. I: Okay, so in providing health talks you meet different people.
14. R: Yes, we do. Depending on the people who have come on that day.
15. I: Okay, how often do you give these health talks?
16. R: Umm, we rotate and so you can give it twice a week or once, it depends on who is there that day.
17. I: Okay, with circumcision, how open do you think the men and women at this clinic would be to talk about it?
18. R: They can be ope because when we are testing for HIV, those who have tested negative ask to say ‘I want to know where I can go to get circumcised’ and we refer them to the right place so that they can get the right information on circumcision.
19. I: The ones who ask are both the males and females?
20. R: Most times, it is the men.
21. I: Okay, lets say they have not started receiving any services at the clinic, maybe they are outside in the waiting area, in that setting, how open do you think they would be to talk about circumcision?
22. R: With the STI clinic, most diseases involve the sexual organs. So, I think if circumcision was brought up, people would not be shy to talk about it in a group setting. Besides, circumcision has been talked about for a long time now and although they only have little information, some have been circumcised and others have not.
23. I: Okay, do you think there would be a difference in how open the men would be versus the women?
24. R: There would not be a significant difference because in the STI clinic, it is mostly the men who come. Women come yes, but the larger group are the men.
25. I: Why do you think that is the case?
26. R: Because with most STIs, when you ask the man if they told their partner, they respond to say ‘my wife is in the village, I slept with a different woman’. When you ask them if they have come with that other person, they tell you that ‘I just met that woman and slept with her’. That is why it is mostly the men who come unlike the women. For the men whose wives are around, those are the honest ones and they come with their wives. When you ask if they are married, they agree and when you ask if they are polygamous, they say ‘no, I am the one who went out and slept with another woman and that is why we have this STI’.
27. I: Okay, so you provide health talks on HIV testing, how open do you think you would be to talk about circumcision?
28. R: I would be open because it is part of our daily work. Of course we know that this is the work that we have, but if there is extra work that has to be done, we just have to know what information we need to give those people for them to understand.
29. I: Okay, apart from knowing what to say, what else would you need for you to be very comfortable talking about circumcision?
30. R: If there were focus groups were we share points on what to talk about with the patients.
31. I: Who would need to be in this focus group?
32. R: Just us, the health workers.
33. I: Okay, We are proposing to conduct intensified health education on circumcision at this clinic. This intensified health education will be group health education talks on circumcision. The education will focus on what circumcision is, its proven benefits as well as the common misconceptions about circumcision. We will also allow patients to ask questions about circumcision and we propose to also involve men who have undergone circumcision and their spouses (women) to share experiences around circumcision. What are your thoughts on using Intensified health education as a way of scaling up VMMC at this clinic?
34. R: It is good because when they have adequate information, that is the patients that might come to the clinic on that day, it means they will approach others with that information. These could be their relatives, the people in their communities or even at work, they can interest others to undergo circumcision.
35. I: Okay and you have said that if they have adequate information…
36. R: Yes.
37. I: For it to be adequate, what should be contained in the education?
38. R: What male circumcision is, Its advantages…the disadvantages… if they knew all that, even when they go home and they tell their relatives, workmates or even church mates, they have enough information such that even if the people have questions, they would be able to provide the right answers. If the information they have is not enough, it is hard for them to convince anyone.
39. I: On asking questions, how open do you think the patients would be to ask, in the group setting where this intensive education is being provided?
40. R: In most cases, people are shy to ask questions in a group. However, when they enter our rooms for assistance, they open up to say “I failed to ask a question while we were outside but I had such a question”. Then that means even us, the health service providers need to have enough information to be able to give them the right responses.
41. I: Okay, so it is possible for them not to ask whilst in the group.
42. R: Yes, it is. There are other people who are quick to speak. When you ask if anyone has a question, they quickly say ‘there is no question’ and so if another person had a question, they will not ask it. You can tell them not to be quick to answer and give a chance to others who might have a question, but because they already blocked them, you find that they just stay quiet. In the rooms however, you find them asking questions.
43. I: Thinking about the topic of circumcision, do you think most people would ask questions in the group or in the rooms?
44. R: I think it would be the same.
45. I: Why is that?
46. R: Because when they are in the group, they ask questions but we see that even when they enter the rooms, they still ask different questions.
47. I: Alright, secondly, we intend to send SMS reminders to men who have a circumcision appointment. This SMS text will be carefully worded or coded for confidentiality as a way of keeping their privacy right,
48. R: Yes.
49. I: The messages will be sent two days before the appointment, a day before the appointment and on the day of the circumcision appointment. Meaning the SMSs are being sent three time right?
50. R: Yes.
51. I: What are your thoughts on using SMS tracing as a strategy for scale-up of VMMC at this clinic?
52. R: It is a good method because for men who told their wives, they can tell their wives to remind them in case they forget. So, they would easily tell their wives that ‘remind me tomorrow, I have received a reminder to go for VMMC’ and the wives would do that. So it is quite good.
53. I: Do you think it would work or not?
54. R: Yes, it would work but it is also a bit hard since not everyone has a phone, there are other people who do not have phones. For those people, it would be hard to get hold of them. For those with phones, it would work perfectly because they might be busy with other thigs but when they receive the message, it would remind them.
55. I: Okay, apart from other people not having phones, what other challenge can you think of with this method?
56. R: Umm, some people may get bored of constantly being reminded. I remember there are times when we call people who … I was working in the retention department once and when you call people to remind them of their visit date, when you call them the first time, they respond positively. The next time you call them, their response is ‘I heard what you said’. There are other people who would respond positively and there are others who would get tired of it, since people differ. Other actually say ‘it is good that you called me, I forgot.’ Yes.
57. I: Okay, in that case, what can be done?
58. R: remind them still. You will be making noise, but at the end of the day, they have heard what you wanted to say.
59. I: Okay, so we are still sticking to reminding them three times?
60. R: Yes, they should still be reminded. They might react and say ‘you are bothering me’ but it would stick in them that on such day, I need to go for VMMC.
61. I: Okay, is there any other challenge that you can think of with this strategy?
62. R: Ah, it is fine. Sometimes, people have phones but their households do not have electricity. There are times when the phone is off, and so you send the message but it will not get through. In this case, sending the messages three times is good because if the phone was off at some point, maybe the next time you send a message, their phone will be on and it will pass through.
63. I: Okay, you also said that not everyone has a phone, what can be done for those?
64. R: For those who do not have phones, then we would need locator information and if they do not come on their appointed date, someone can follow them to their homes to say ‘we waited for you to come to the clinic but noticed that you did not come. So we are here for you to set a date when you think you would be free to come to the clinic’.
65. I: Okay, so someone would need to follow them to their homes.
66. R: Yes, because not everyone has a phone. We use the same method in retention, some people have phones and others do not have phones. When they do not come on their visit date, we give them a period of some days and if they still do not come, they are followed and they give different reasons to why they failed to come to the clinic on their scheduled date.
67. I: Alright. We are also proposing to provide transport reimbursement to men who will undergo circumcision to help with expense incurred on the day of circumcision. The reimbursement will be an equivalent of $10 in Malawian Kwacha based on the National Health Sciences Research Ethics Committee guidelines. The reimbursement will be from a designated nurse within the STI clinic. What are your thoughts on this strategy?
68. R: That one is also good. We see people who have tested positive for Syphilis, these people need to come to the clinic three times. You find that someone fails to come for all the three times and when you ask them why, they tell you that they did not have transport. So, this would be one way of encouraging them to return to the clinic. The way money is hard to come by nowadays, people would be reluctant to come back to the clinic. They would reason to say ‘this money is enough to buy my day’s food’ so they would rather use it for that and they give up on coming to the clinic. However, if they were given transport, it would be a way of encouraging them. Whether they decide to use the money for transport or not is up to them. When that money has benefited them, they can share the information with their friends to say “go to Bwaila, they are giving out money for transport. I bought the food I ate yesterday with that same money.” In that one, other men would come to the clinic. But, for you to just tell them to come to the clinic on a certain day, some people need to pay k700 or k500 just to come to the clinic and they would rather buy food for themselves.
69. I: Okay, so the first benefit of this strategy is that they will tell other people about it and even they will really come on their scheduled date.
70. R: Yes.
71. I: What other benefits can you think of with this strategy?
72. R: Aa, I think those are the main ones. If they have children, those above 18 years old, or if anyone in the family wanted to get circumcised but were reluctant because of the transport, they would be encouraged to come.
73. I: Okay, what challenges can you think of with this strategy?
74. R: Challenges with reimbursing transport?
75. I: Yes.
76. R: If the money is finished and we can no longer provide transport money, it would seem as though we cheated the people.
77. I: Alright, finally, we want try implementing all the interventions discussed above together to see how they will impact of the number of men who will choose to get medical circumcision. What are your thoughts on combining and using all these strategies at once?
78. R: Just to add, if there was an opportunity to go into the communities, approach the chief and tell him that ‘we are coming on such a day so please gather the people. We want people to be encouraged to do VMMC at the clinic.’ That would work. There are some areas which make it hard for people to come to the clinic, the areas that are far from town anyway. That would help us get more people from the areas that do not have clinics offering circumcision near them.
79. I: Okay, so having meetings.
80. R: Yes, even doing them once a month. You gather them and ask them when they plan to come to the clinic and then remind them when their date is due. Yes.
81. I: Okay, in this one, we are going to the communities, to the people. Lets think of it in the clinic setting, the people have come to the clinic and all these strategies are in place. How effective do you think they would be in increasing uptake of VMMC?
82. R: I think that the intensive education would really be effective because if the people have adequate information, they are a lot more in number than we are and so they can reach out to a lot more people. If they have been taught well, they can approach other people in the community.
83. I: Okay, which combination do you think would work better together?
84. R: Intensive education and transport reimbursement are the ones I feel would be effective. For the SMS ones, yes there are people who have phones, but when you ask them to show you a contact in their phone, they are unable to and then need someone to read the message to them or for someone to find the contact for them before they can call. We know that education is not really a priority in Malawi [many people cannot read]. When you teach someone however, they will not remember everything but they will still have one or two points in their head, which they can use to approach other people and when those people come, the clinic team can add on to the little they already heard.
85. I: Considering the activities that already take place in the STI clinic, how do you think these strategies fit into that?
86. R: They fit in because we say that one who is circumcised is at lower risk of contracting HIV. They can still contract it, but if I am not mistaken, they reduce the risk by 60% and that would help them. When they hear the other advantages that are there as well like promoting hygiene and preventing them from cancer, they would be compelled to do VMMC.
87. I: Okay, so it would fit into what already happens in the STI clinic?
88. R: Very much! They both involves the sexual organs as well.
89. I: Okay, what about in terms of workload.
90. R: The work load is already a lot, but we would find a way of managing. Previously, there were three counsellors and I was sent to work in another department. But, they called me back to the STI clinic and am sure that was because they realized that the workload was high at the STI clinic.
91. I: Okay, and what will happen when we add this?
92. R: We will find a way. It’s not like everyone will agree to undergo VMMC after the talk, some will still refuse. For those who have agreed to do it, we will find a way of managing. We might be slow at the start but as time goes, we will get used to doing it.
93. I: Okay, and in terms of our culture or the different religions, how do you think the issue of VMMC would play out?
94. R: At first, people used to say that VMMC is for Muslims. Currently, with the coming in of medical circumcision and other talks, people know that it is not just for the Muslims, it is for everyone. I think the Muslims do not cut out much of the foreskin, whilst the medical circumcision does that. That is one way they can protect themselves from sexually transmitted diseases once their foreskin is out and it is dry.
95. I: Okay, so the uptake will not depend on whether one is Muslim or not?
96. R: No, it will not. Even in our households, our children do not tell us that they are going for circumcision, you only notice it when they back. After the first people get circumcised and there is no problem, those are the people who will go out and encourage their friends. Some worry that once they are circumcised, they will not be able to engage in sexual behavior, so that would help.
97. I: Okay, is there anything else that you would like to share with me?
98. R: Aa, no. that is no.
99. I: Okay [chuckles] do you have any question?
100. R: My question is that, if the people chose to be circumcised on one day, would it be possible for all of them to get circumcised? Say 20 men chose the same day, will all of them get assisted on the same day?
101. I: Okay, that is a good question. But, what do you think we could do for that to work out?
102. R: I think that there should be communication when scheduling them because if not, you will find that all of you scheduled the men on the same date. For them to come to the clinic and then be sent back, some men leave work or their businesses to come here and it would not work for them to be away for more than a day [if they came and were told to come on a different day because of the number of men that day].
103. I: Alright, thank you very much, this is the end of what I had.
104. R: Thank you.

THE END
