# Supplementary material for: Acceptability, feasibility and appropriateness of intensified health education, SMS/phone tracing and transport reimbursement for uptake of voluntary medical male circumcision in a sexually transmitted infections clinic in Malawi: A mixed methods study
Source: PLoS One. 2025 Jan 24;20(1):e0301952. doi: 10.1371/journal.pone.0301952 (PMC11760565; doi:10.1371/journal.pone.0301952)
Supplement: S1 Data — (ZIP) [file pone.0301952.s004.zip › Qualitative data/Baseline IDI Transcripts/Transcript 3.docx]

1. I: The consent and the information we will collect while recording are kept in two different places. Apart from that, whatever information you are going to share with me now, while we are recording, will not be saved using your name, it will be saved using the ID that you have been given. None of the things you say will bear your name.
2. R: Alright, you can go ahead.
3. I: Okay, tell me about your role at this clinic, at the STI clinic.
4. R: Am an (Withheld).
5. I: What do you do as an (withheld)?
6. R: We provide counselling on HIV and we test for HIV.
7. I: Okay, when you get to work, briefly walk me through what you do on daily basis, on a normal day.
8. R: Okay, when we arrive, the first thing we do is to give a health talk on HIV. We explain how HIV relates to STI’s. Since we test the patients for HIV before they are assisted, some tend to ask how HIV is related to STI’s and so to avoid receiving such questions from individuals, we give them basic information on HIV and how it is related to STI and we continue with the other things we need to do. From there, we test them for HIV.
9. I: After that, you are done with them?
10. R: From there we send them to receive treatment.
11. I: Okay, how long have you been in this role?
12. R: Since 2016.
13. I: Roughly 5 years right?
14. R: Yes, 5 years.
15. I: Okay, so you give HIV health talks.
16. R: Yes, how it is related to STI.
17. I: Okay, think of the people you talk to, the ones that come to the STI clinic, how open do you think they would be to talk about circumcision?
18. R: [Silence] everything has a beginning and so if we planted that in them, if we combined circumcision in the health talk, I think it would not be a problem to them. The same thing with HIV, it started and people accepted it. We give them the talk and they understand, I do not think circumcision would be difficult.
19. I: Okay, if you were to compare the males and the females; who do you think would be more open to talk about circumcision or would it be the same between the men and the women?
20. R: [Silence]
21. I: Would there be any difference between the two or not in terms of talking about circumcision?
22. R: I think the men would be more interested with the talk because it concerns them more than it concerns the women.
23. I: Okay, so the interest is coming because one is concerned about it and the other is not.
24. R: Yes.
25. I: Okay, is there any other reason why you think the men would be more interested?
26. R: Aa, no.
27. I: Okay, still on the men, apart from them listening and understanding, how would they react to the discussion around circumcision?
28. R: Not everyone would be welcoming to it because we know that most men who live here in town are out looking for jobs to get money. So, because of time, some would still complain, not all would be receptive.
29. I: Okay, so they would complain on time.
30. R: Yes, to say ‘you told us about the HIV and now you are here telling us about circumcision and then we have to go and get tested and all that’. Most people complain on time, because they want to go and fend for their families. However, in terms of the message, I do not think they would complain. They would only complain about time.
31. I: Okay, personally, how open are you to talk about circumcision?
32. R: There is no problem for me because it is part of my job [laughs]. It is my job to give the message to the clients.
33. I: What would you need to give the message to the clients?
34. R: In terms of what?
35. I: I do not know; if you were to talk to people about circumcision, what would you need?
36. R: Okay, we need guidelines to follow… I do not know if I am making sense… okay, we need to have guidelines on what we need to say when we are giving the talks. These need to be written or on a paper we can refer to, to say, from here we will go to this topic and so on. When you say so many things people get bored.
37. I: Okay, we are thinking of having intensified education on circumcision at this clinic. This education will be done in groups regularly, I think in the same context you talked about.
38. R: Yes.
39. I: The education will focus on what circumcision is, its proven benefits and common misconceptions about and we will also allow patients to ask questions about circumcision. We propose to involve men who have successfully undergone circumcision and their spouses as well so that they can share experiences around circumcision. What are your thoughts on using this intensified education as a way of increasing the uptake of VMMC at this clinic?
40. R: I do not understand the question [laughs]
41. I: Okay, you have understood the intensified health education I have explained?
42. R: Yes
43. I: You told me that you give health talks right?
44. R: Yes.
45. I: In this case, we are giving health talks but the topic of this one is circumcision. The aim of this talk is to increase the number of men coming to the clinic for circumcision.
46. R: Like sensitizing.
47. I: Yes, but these are just our thoughts. My question is, what do you think about the thoughts that we have; do you think it would work or what would happen if we used the intensified health education as a strategy to increase uptake?
48. R: Okay, it is good. I am saying it is good because people in the communities have misconceptions around circumcision. So, I think if most people came to the clinic to listen to that talk, they can have some knowledge and realize to say ‘the things we tell each other at home are wrong, what the hospital people are saying is what is true’. In that way, the message will continue to spread since the people talk. even while the men are talking, it could be while they are playing a game or anything, while doing that say the topic of circumcision comes up and they start to talk about the misconceptions that are there. If someone who came to the clinic and heard the talk is there, he can guide the others and tell them what is true. That would bring some curiosity in the other men to say ‘that is what is happening at the hospital, I should also go and experience it.’ In that way, many people would come.
49. I: Okay, so first, the misconceptions are removed and when they go home, they can help to clear out the misconceptions there.
50. R: Yes!
51. I: But, what information do you think should be included in this intensified health education on circumcision?
52. R: I would have loved to hear the information that is already there [chuckles] because I might mention things that are in it already.
53. I: Right now, these are just thoughts, things we are planning to do. That means everything you share would be helpful.
54. R: Okay, the information we can give people on circumcision?
55. I: Yes, what information should be included in the intensified health education?
56. R: Okay, things like what circumcision is, as you said earlier. I think everything needs to have an introduction so that is an important point. The people also need to be told the relationship between HIV, STI and circumcision; they need to know those things. And the other things that you mentioned.
57. I: Things like what?
58. R: [Chuckles] what circumcision is, the misconceptions that are there concerning circumcision; those things need to be included. For instance, people say that ‘when we are circumcised at the hospital, they take the foreskin and use it for other things’ like it is used for satanic purposes. So, when giving the education, they need to be told that some of the things that you have heard like this and this are not true. This is what we do when we cut off the foreskin and things like that.
59. I: Okay, you also talked of the need to explain the relationship between HIV, STI and circumcision. Why should that be included?
60. R: Okay, this study will be conducted at the STI right?
61. I: Yes.
62. R: The person I going to the clinic for STI treatment but before they can do that, they are tested for HIV and therein as well comes circumcision. So, these things need to be well prepared so that they see how the things relate to each other. When they are told to test for HIV, they should not be surprised or confused and when they hear of STI or circumcision, they should not be lost. I do not know how the information would be arranged but the relationship needs to be explained.
63. I: Okay, and the issue of bringing in people who have undergone circumcision or their wives as a way of scaling up uptake. Do you think it is necessary or not?
64. R: It is very necessary.
65. I: Why?
66. R: Because people will be encouraged to do it from them. There are some people who do not undergo circumcision because of their cultures and there are people who have undergone circumcision because of their culture. In our case, we are not leaning on the culture; we just want people to get circumcised. We might find people who are circumcised and yet their culture does not allow it, those people can come to share their experiences to say ‘I am Chewa and we believe that the Chewa do not go through circumcision but I did’. In that way, those who still have it in them that the Chewa do not get circumcised will be encouraged to take part in the circumcision.
67. I: Okay, thank you very much. We also plan to send SMSs as a way of reminding the men who have a VMMC appointment date. This message will be carefully written or coded as a way of ensuring privacy. This will be sent 2 days before circumcision, then again 1 day before circumcision and the final one on the day of circumcision. In total, they will receive 3 SMS reminders; 2 days before, a day before and on the actual day of circumcision.
68. R: Okay.
69. I: What do you think about this method as a way of increasing VMMC uptake at this clinic?
70. R: [Silence] it is a good method.
71. I: You seem hesitant in your response [chuckles]
72. R: [Chuckles] no, I am not.
73. I: Okay.
74. R: But… I was just lost for words [laughs]
75. I: [Chuckles] okay, so tell me why you think it is a good method.
76. R: As I said, most men are busy, that is what we believe, that the men are busy. They came to town to find money to feed their families. So, I do not think that if we told someone today to come on the 26^th^ next month for circumcision, only a few would remember their appointment date. Some would not care; they would say that so long as they have received assistance for their STIs, the rest does not matter. Therefore, I think this is a good method; it will be a way of reminding the people to come to the clinic. For those who do not come, it is because they have decided not to come. However, it is a good method.
77. I: Okay, what challenges can you think of that would come about with this strategy?
78. R: The challenges?
79. I: Yes.
80. R: The challenges that I can think of, I do not know if this would really happen but I think that some people would get bored with the messages. Sending them a message or receiving a message is the same as someone reminding you of something you owe them. If they remind you today, tomorrow they also remind you and they remind you the again the next day, you get bored with them, so it is the same with this strategy. Some would not like it and some would even call the number [chuckles] that was used to send the SMS to scold [to curse]. They would call to say ‘why do you keep sending these’ and many other things. Some would be okay with it and so some would come and others would not.
81. I: Okay [chuckles] I have heard. Is there anything else you can think of?
82. R: Aaa, [sighs] that is all.
83. I: Alright, finally, we are also thinking of reimbursing transport to men who will undergo circumcision to help with expense incurred on the day of circumcision. The reimbursement will be an equivalent of $10 in Malawian Kwacha based on the National Health Sciences Research Ethics Committee guidelines. The reimbursement will be from a designated nurse within the STI clinic. What are your thoughts on this strategy as a way of scaling up the uptake of VMMC at this clinic?
84. R: It is good.
85. I: Okay, why is it good?
86. R: There will be a high turn-up of people because… when people receive money, as I explained at the beginning, after you teach them, they will tell each other what they have learnt and it is the same with the money. When they get the money, they will tell their friends to say ‘we have been given k7000 after circumcision. If you are not circumcised, you can go to the clinic’. Because of the money, people will come for circumcision.
87. I: Okay, so many men would come.
88. R: Yes, they will come because of the money. At the end of the day however, it will not matter why they came so long as they have undergone circumcision.
89. I: Okay, what else would make this strategy good?
90. R: The transport reimbursement one?
91. I: Yes.
92. R: Aaa that is all. The men will come and que here [laughs]. They will no longer go in search of money to buy food, they will find that money here [laughs].
93. I: Okay, and what are the downsides of this method?
94. R: This one would not have any disadvantages. A person would not claim to have never been circumcised because the nurse can easily see and tell if they are circumcised or not. Unlike the other studies… I do not see any problem with this one.
95. I: Okay.
96. R: Are the any challenges you thought about?
97. I: No, which is why I want to hear from you [chuckles]. Lastly, we intend to try implementing all the interventions discussed above together to see how they will impact the number of men who will choose to get medical circumcision. What do you think about combining and using all these strategies at once?
98. R: [Silence]
99. I: We first talked of intensive education on its own, then we went to SMS reminders and then we talked of transport reimbursement. Now, we want to implement all these strategies together; what do you think about that?
100. R: Combining them when giving the information to the people or?
101. I: In this case, what would happen is that when the people come to the clinic, they will have the intensive education and from there, they will be given an appointment date and the SMS reminders will be sent to remind them. When they come to the clinic on their appointment date, they would be reimbursed. What do you think about combining them?
102. R: They are fine.
103. I: Do you think they would work?
104. R: They would work.
105. I: What makes you say that?
106. R: [Chuckles] I have failed to answer that question [laughs]
107. I: [Chuckles] okay, what do you think in terms of workload or do you think it would be too many activities?
108. R: This is work done with other people right? If that is the case then the workload is not there. It will seem to be a lot of work if the number of men who want to get circumcised is a lot. However, if working as a team, there is no problem. One would give the talk, another would be distributing stipend while others get the people to get circumcised. Since there is that team work, it would not be hard except when the number of men who have come is a lot.
109. I: Okay, what about on the part of the patients, do you think it would be too much or not?
110. R: For the patients it would be… yes, that is too much for them.
111. I: Okay, why?
112. R: I said earlier that these men are time conscious so that they should still have enough time to go and look for food. Therefore, we give them… I do not know what will be happening, will the person get medicine where they have been circumcised?
113. I: Medicine like?
114. R: For their STI treatment. I do not know about that. However, if the person has opted for circumcision and they get their STI medication at the same place that will not be a problem. However, if the one undergoing circumcision has to go to another room to get medication, it will be hard and there will be chaos. If we have taken someone who wants to undergo circumcision, if we have taken him and he has agreed to go through it, we need to assist this person fast so that they get assistance from the nurses. If this person comes and finds that people have queued, that would bring chaos and those people who are already in the line would start scolding thinking that this person is cutting in line. However, if they get whatever assistance they need where they are going to get circumcised, I do not think there would be any problem. But if they need to come back and get treatment, that would be a big problem.
115. I: The problem would come in terms of treatment.
116. R: Yes!
117. I: Other than that, it would not be too much for the patient to receive education then get an SMS reminder and then get transport reimbursement?
118. R: It would depend on the patients themselves but in my case, I do not see any problem.
119. I: Okay, if you were to choose which two strategies to combine or maybe if you were to choose one strategy, which you feel would work individually, which one or which ones would you choose?
120. R: [Silence]
121. I: There is intensive education, SMS reminders and transport reimbursement.
122. R: [Silence] the ones I would like to combine are the SMS and the intensive education.
123. I: Okay.
124. R: Because even if there were no stipend, people would come since circumcision started a while back. Of course not a lot of people are getting circumcised, but people are still getting circumcised and everyone knows that circumcision is for free and you do not get anything. If there were no stipend, I still think people would come. The stipend would only help so that the turn up is high but without the stipend, if there was just the SMS and intensive education, I think it would work.
125. I: If you were to compare it with SMS and intensive education, which one do you think would bring about most men?
126. R: The stipend would bring many people.
127. I: Okay, and you opted for SMS and intensive education, how do you think the uptake would be if those two were combined?
128. R: [Sighs] [laughs] that one is also hard
129. I: Okay,
130. R: Am confused [laughs] I think I am contradicting myself.
131. I: [Chuckles] okay, let me hear what you have to say, it is okay to contradict yourself.
132. R: If we took out the stipend and we were left with the SMS and education, the education is necessary and we cannot remove it.
133. I: Why?
134. R: People need to know what circumcision is, its benefits, how it is done if possible. Of all the strategies, the education is the important one. The SMS is just a reminder, so that we do not completely ignore them. To me, even if the stipend were not there and we were just giving out the other two, there would be no problem. People would still come although the turn up would be lower than if there was stipend being given.
135. I: Alright, I understand. Thank you very much [chuckles]. I know you mentioned this earlier that we would need to establish the relationship between STI, circumcision and HIV. However, how do you think the strategies we are talking about fit into what already happens in the STI clinic?
136. R: Which ones?
137. I: The three strategies we talked of; education, SMS and reimbursement. How well do you think they fit into what happens at this clinic? Do you think they fit into what happens or they are completely foreign?
138. R: [Silence] they are not foreign; these are things that happen. We had the Iknow study in the STI clinic and people were being given money. Before enrolling into the study, they were told of the study. The only new thing would be the SMSs because I do not know if they were sending out messages [chuckles].
139. I: Okay, considering our culture and religious beliefs, how well do you think these strategies fit into that?
140. R: [Silence] I do not understand the question.
141. I: Okay, how well do …
142. R: What number is the question you are asking?
143. I: We are almost done, sorry. How do you think these strategies fit into our religious beliefs or culture?
144. R: How they fit?
145. I: Yes, or do you feel these are against our culture and religious beliefs or not?
146. R: They are not against it.
147. I: Why do you say that?
148. R: Because there are religions who believe in circumcision, our Muslim friends for instance. At present, the belief that only Muslims undergo circumcision is not there; even Christians are being circumcised. Therefore, it is fine and it is not against religion or culture.
149. I: Okay,,
150. R: It is okay. Currently, circumcision is not something new. People know about it, they know what it is and they have information about it, they are not completely blank. When it comes or when we are giving the education, it will not be new to them.
151. I: Okay, it is something they already know.
152. R: Yes.
153. I: Alright, is there anything else you would like to share me?
154. R: To what?
155. I: To share with me concerning circumcision or what can be done to increase uptake of VMMC or anything that you would like to share with me.
156. R: Aa, no. there is nothing.
157. I: Okay, do you have any questions?
158. R: Okay, after the SMS is sent to the people, I believe you will have a register right? Where you will record or document anything to show that this one… will you use the ID numbers or you will use the names of the people when this starts?
159. I: Okay, we have not gone that far, but I would like to hear from you. What are your thoughts on us using a register or using IDs?
160. R: Okay, I had a question on the register or on the IDs to say ‘you send a message to someone…okay, let me ask it this way, you have said that you will send the message a total of three times right?
161. I: Yes.
162. R: If the person does not come, will you continue to send the messages or that will be the end?
163. I: Okay, that is a good question but I will turn it back to you to say what do you think we should do? If we have sent three messages to the person and they do not show up, what should we do?
164. R: Okay, I will give you an example of what are doing at HTS. We are doing what we call the VAPN study, which now changed to AIT. When we call the person the first time… we are supposed to call the person three times. If we call the person today for instance asking them to come and the person promises to come on a certain day. If the person does not come on that day, we are supposed to call the person to remind them that ‘you told us you would come on such a day but you did not’ and they might say I will come on such a day instead. If they do not come on that again, we call them back and they might give us another date again. If they do not come on the third attempt, we are supposed to follow the person. We get locator information at the beginning and so we follow that person. It could be that they failed to come because they are busy or because they are just reluctant, so we follow them. That is where my question was coming in to say if you send messages to this person three times and they do not come, what will you do?
165. I: Okay, what would you suggest?
166. R: [Silence]
167. I: [Chuckles] what would you suggest?
168. R: I think following them would be a good thing. With the issue of stipend, people will most likely say ‘so long as I have received the money, I am done’. Am not sure when they will be given the stipend, before or after circumcision?
169. I: After circumcision.
170. R: Okay, now I understand. If they do not come and you have the necessary resources, they can be followed. You would tell them that we sent you a message and you did not come and so we have decided to follow you. However, before you follow them, you must let them know. Do not just show up at their house, people might not like it.
171. I: Alright, any other question?
172. R: Aa, no, I do not have.
173. I: Okay, this is also the end of the questions, which I had. Thank you very much for your time today.
174. R: Thank you.

THE END
