# Supplementary material for: Acceptability, feasibility and appropriateness of intensified health education, SMS/phone tracing and transport reimbursement for uptake of voluntary medical male circumcision in a sexually transmitted infections clinic in Malawi: A mixed methods study
Source: PLoS One. 2025 Jan 24;20(1):e0301952. doi: 10.1371/journal.pone.0301952 (PMC11760565; doi:10.1371/journal.pone.0301952)
Supplement: S1 Data — (ZIP) [file pone.0301952.s004.zip › Qualitative data/Baseline IDI Transcripts/Transcript 10.docx]

1. I: Thank you for taking your time to speak with me today.
2. R: Yes.
3. I: I want us to talk about an intervention being tried at this clinic known as RIT. The R is for reimbursement for transport, I is for intensified health education and the T is for SMS Tracing. We are trying this intervention to see if it can improve men’s desire for medical circumcision and increase the number of men going for circumcision. All your responses are confidential. Your name is not on this form. If you cannot, or do not wish to answer a particular question, tell me and I will go on to the next one. Please answer questions honestly and there are no right or wrong answers to these questions. Do you have any questions before we start?
4. R: No, I do not have.
5. I: Okay, first, tell me about your role at this clinic.
6. R: I work as a (withheld) here at the STI clinic, and I am working with UNC project as well. We help with providing medication to those who have come to the clinic with STIs. In terms of research, we work in HIV related studies.
7. I: Okay, when you arrive, on a normal day, what are the things that you do?
8. R: When we arrive, we first clean our work area. We are supposed to clean the area, make sure we have wiped the surfaces. We also need to ensure that we have the medicine that our clients are supposed to receive as well as making sure that all the tools we need are available in the room. We do that in order to avoid going in and out of the room while the client is there.
9. I: Okay and how long have you done these things? How long have you worked in the STI?
10. R: About 17 years.
11. I: At the STI clinic?
12. R: Yes, since 2003. Of course, I left for about 2 years; I worked for a different study in Tidziwe. Afterwards, I returned to the STI clinic and I have been here to date.
13. I: [Chuckles] Alright. That means you have been seeing the different people that come to the STI clinic not so.
14. R: Yes.
15. I: Okay, on acceptability and appropriateness of circumcision, how open do you think the male or female clients would be to talk about medical circumcision?
16. R: I think they would be open to talk about it because medical circumcision is really accepted. Currently, with the way it is at the moment, medical circumcision is accepted because it offers more hygiene unlike the one done in the communities. Medical circumcision helps to prevent other diseases like HIV. With non-medical circumcision, it is possible that the tools are not taken care of. We do not know what really happens, but from what people say, they can use the same equipment for different people, which makes people prone to contracting HIV. That is what I think.
17. I: Okay, and you said that people would be open.
18. R: Yes, they would be open with medical circumcision.
19. I: Okay, and you continued to say that they would be open to talk about it because of the way things are at the moment.
20. R: Yes.
21. I: Why do you think people would be open to talk about it now than they would have been previously?
22. R: Because circumcision has its benefits. It has many benefits. At the hospital, they are told of the many benefits of circumcision. With non-medical circumcision, they might only be told of a few benefits of circumcision whilst when they come to the hospital, they meet people who are well informed and they tell them of many benefits.
23. I: Okay, if we can compare how open the men would be with how open the women would be, would there be any difference in how open they are to talk about circumcision?
24. R: There could be a difference because of how the women understand it and how the men understand it.
25. I: Okay, please explain what you mean [chuckles]. I have heard what you have said, but I do not understand.
26. R: [Chuckles] The men might be more open because they are the ones who will have to go through the VMMC whilst the women might not be open because it is something the men will have to go through and not they.
27. I: Okay, and at this clinic, how would the men react to talking about medical circumcision, if someone were talking to them about circumcision, what would be their reaction?
28. R: If it were their first time coming to the clinic? Maybe you should ask that question again, I did not understand.
29. I: Okay, how do you think men at this clinic would react to discussing medical circumcision?
30. R: [Silence] some can be open but others would not because there are other people who would like to consult first. For married people for instance, they would like to consult their wives before they can make a decision. They can make a decision on their own but their families might not agree and that would bring about some misunderstandings between the man and the woman as to why he got circumcised without telling her.
31. I: Okay, so they would want to consult first.
32. R: Yes, others would want to consult their relatives, depending on their culture, some might want to consult their uncle or their brother. For the young men, they might consult their mother or their father.
33. I: Okay, and how open are you to talk about circumcision?
34. R: I am open to talk about circumcision because it has benefits. The benefits are; it reduces the risk of contracting STDs like Candida. If someone is not circumcised, most men do not clean up well and so they might have some things that can cause candida. I can also be open to talk about circumcision because it helps reduce the risk of cancer for either the women or the men. Circumcision also helps with… some people say that it helps the men with erection, so that they remain erect until they are sexually satisfied. That is how open I am.
35. I: Alright, let us now talk about some strategies that we want to put in place.
36. R: Yes.
37. I: We are proposing to conduct intensified health education on circumcision at this clinic. Intensified health education will regular group health education talks on circumcision, like in the waiting area for instance. The education will focus on what circumcision is its proven benefits and common misconceptions about circumcision. We will also allow patients to ask questions about circumcision. We propose to involve men who have successfully undergone circumcision and their wives to share experiences around circumcision. What are your thoughts on using intensified education as a way of increasing VMMC uptake at this clinic?
38. R: Ask the question again. I have understood the explanation, but I did not understand the question. If there is another way of asking it [chuckles]
39. I: Okay, I have explained how the education will be right?
40. R: Yes.
41. I: The aim of having this intensified education is to increase the number of men coming to the clinic for circumcision. How do you think using this education as a way of increasing numbers would work out? Do you think it would work? How would it turn out? What would be the challenges? Things like that.
42. R: That is a good strategy and there would be no challenges because people really trust hospital personnel to know their job, and they would explain it very well to them. When the people come to the clinic, they come in a group. Within that group, you can have others who are already circumcised and those people can comment on the benefits of circumcision. They can tell their friends there what happens during circumcision or what happens after. People like those can make the ones who have not been circumcised interested in it. At the end of it all, when the medical personnel explain to them about circumcision, they become interested. It is possible that they heard about it but did not really understand. However, when they come to the clinic and they understand, it will help them make the decision to get circumcised.
43. I: Okay, can you think of any challenges that would come about with this strategy?
44. R: The challenges could be there, like time for instance. The people that come here run businesses and some of them are rushing to work. [Chuckles] maybe they would not be interested to listen, they just want to get assistance for the diseases they have come with and then leave. They would not be interested to listen, they only want help for the problem that they have. I think that would be one challenge. Another challenge that might be there is if the VMMC will not be done on the same day. Normally, when someone has STIs, they are told to go home and finish the treatment they have been given. When they are healed is when they can get circumcised. The challenge that would be there is that once those people are fine, they might be reluctant [lazy] to come to the hospital for VMMC. Another challenge that can be there is transportation, considering our income. Some walk to the hospital on the day they have reported to the clinic with the STIs. When they think of undergoing circumcision and yet they are not sick in any way, they can also get discouraged because they do not have transport money. When they go home after they have received the treatment, they meet friends or family members who would also discourage them. This, is especially from those who are not interested in circumcision, they can discourage their friends.
45. I: Okay, for the first challenge you mentioned the issue of time. You said others would rather get assistance and then leave. What can be done to make sure time is not a challenge when delivering this education? What could be done?
46. R: I think that maybe…maybe if the talk [education] was summarized, it should be brief but understandable. There should not be so much that is said, it should be brief but ensure that the people have understood. That is what I think [chuckles].
47. I: [Chuckles] okay, what important information should be in the brief talk? What type of information should be included?
48. R: Okay, the people need to know what circumcision is. They should also know the place where they can get the circumcision done; they should know the benefits of the circumcision as well. Just the main points and I think those could be the main points.
49. I: Okay.
50. R: Another thing…I do not know. Maybe I will add some irrelevant things [laughs]
51. I: It is fine, let us hear it. So long as that is what you think, let us hear it.
52. R: What I was going to say is after the circumcision has been done and not before, so maybe that is…
53. I: Okay, say it, after circumcision
54. R: Maybe if they experience something bad, something scary and they think they cannot just stay home, even if it is before their review appointment date, they should know what they could do. They need to be told those things. They can experience some scary things, which would disturb them, and if they just stay home, they can scare other people in the community to say ‘he went for circumcision and bad things are happening to him’. Things like those.
55. I: Okay, they should be taught what circumcision is, the benefits and what to do if they experience anything negative.
56. R: Yes.
57. I: Okay, can you think of anything else that we should include in this education?
58. R: No, there is nothing else.
59. I: Okay, you mentioned transport as a challenge. One of the strategies being tried is transport reimbursement on the day of circumcision. This money will be the Malawi Kwacha equivalent of $10 as a way of covering the expenses made on the day. This will be given through set nurses in the STI clinic. How do you think this would do in increasing the number of people coming for VMMC at this clinic?
60. R: The transport will help because people’s earning is different. Most times, the people that come to a government hospital like this one do not earn a lot of money. Most people tell us that they came to the hospital by foot. Those who are financially well can go to private clinics without a problem. However, either the ones that come here run business or some are not even working. They rely on piece works. As such, in terms of the transport, it would help in encouraging the people to say ‘when I go there, they might give me transport money’.
61. I: Okay, what challenges can come up with this strategy?
62. R: The challenges?
63. I: Yes.
64. R: The challenges that might be there… we can say it is a challenge, but it might also not be a challenge [chuckles]. When people hear of transport, they can get coaxed to come for VMMC. It means the VMMC hospital will have an increased number of clients meaning people would have to wait a long time before they are circumcised. That would make them bored, because of the waiting time due to their numbers. On the other hand, some people will get circumcised just for the sake of… what can I say? [chuckles]
65. I: Yes,
66. R: They would only get circumcised because of the money. Because they would know that, they will have some change [leftover money] that they can use to support their families. They would unwillingly come for the circumcision, so long as they are given the money. That is because money is not easy to earn.
67. I: Okay, for that part where there are many people coming in and some are getting bored because of the waiting time; what can be done to correct that?
68. R: They should not stay at the clinic for long and that means the places for VMMC need to be many, they need to be a lot. If we are saying there needs to be more places, then the staff should also be a lot so that people do not wait long at the clinic.
69. I: Okay, is there anything else you can think of?
70. R: [Silence] in terms of giving them transport money, this is a program sort of, and it will run for a short period. When the program ends, I do not think the government would be able to give people money after they have come for circumcision. That would be a problem. As a result, other people in future will be reluctant to come for VMMC and the numbers will go down again.
71. I: Okay, while it is running, people would be there but once it ends, the number may go down?
72. R: It will go down yes because they will say ‘they do not give transport anymore’ and people will be discouraged.
73. I: Okay, I understand. We have talked about intensive education before the circumcision and reimbursement after the circumcision. However, before the circumcision, when they have been given an appointment date, there is another strategy we are thinking of, which is sending SMSs as a reminder for their appointment date. This message will be sent 2 days before the appointment date, a day before the appointment and on the day of the appointment; three times in total.
74. R: Yes.
75. I: This message will be worded carefully or encrypted as a way of ensuring privacy. What are your thoughts on this strategy as a way on increasing VMMC uptake at this clinic?
76. R: [Silence] SMSs
77. I: Yes.
78. R: It will help because when the men have been given the appointment date, they can forget because of the different things they are busy with. Things like businesses, work and other ways of earning money to feed their families. The SMS reminders will help them, because once they receive the message, they will be reminded. That will also make them interested to say ‘those people really trust that I will do circumcision’. That would strengthen them to say ‘it means the hospital personnel are looking out for my health. Let me go and get circumcised.’
79. I: Okay, they would be interested, strengthened and encouraged.
80. R: Yes.
81. I: Okay, how else would this strategy help apart from that?
82. R: [Murmuring] eeh! I think that is the only one I can think of as of now [laughs]
83. I: Or the challenges that may be there with this strategy, what can you think of?
84. R: The challenges could be that the person does not have a phone. they might have had a phone when they gave the number but with time, it is possible the phone got lost or broken. Others give numbers that are not theirs, maybe their relatives. You find that they live very far from the person who has a phone or the person lives in an area that has poor connection. Those are some of the things.
85. I: Okay, what can be done to resolve these challenges?
86. R: The plan is only for SMSs not physical tracing [laughs]
87. I: What if it was there.
88. R: Then we would get locator information on the day they have come to the clinic, they day you have met them and explained VMMC to them. Locator information can be taken for those that are interested so that if the phone does not work, they can be reached through physical tracing.
89. I: How would the people react to that? The patients that is.
90. R: When getting the locator information, we are supposed to tell them that such a person will come to visit you. When going for tracing they are not supposed to wear a uniform, so that the people in the area do not recognize the people who have come. When taking the locator, we tell them that it is confidential and you get their consent on whether you can go to them or not. Apart from that, you ask them how you should identify yourself. Some people do not want the people around them to know that you are hospital personnel, so you ask the client to tell you how you should introduce yourself.
91. I: Okay, and on sending the SMS to the client, how do you think they would react to receiving a message from the clinic? Apart from being encouraged, how else would they react?
92. R: A negative or positive reaction?
93. I: Any kind of reaction; what kind of negative reaction would they have or what positive rection would they have?
94. R: I do not think they would react negatively. When getting their phone number, it means you talked about it. As such, I do not think they would react negatively, but positively. As I said, they would get interested and say ‘the people still remember me. It means they want what is best for my life.
95. I: Okay, finally, we would like to try and implement all the strategies we have talked about at once, to see how they would impact the number of men opting for medical circumcision. What are your thoughts on implementing all these strategies at once as a way of increasing the uptake of VMMC?
96. R: [Sighs] this will help
97. I: [Chuckles] and you have let out a very big sigh [laughs]
98. R: [Laughs] it would help and the reasons are the ones I have already given. With the SMS, they would think that the people are interested in them and they want what is best for their health. With the reimbursement, people would be interested because travelling here needs you to use transportation. Most people use buses to get the hospital, not so many of them live nearby. Giving them the transport money will help with their coming to the clinic, for them to be interested to come for VMMC. What was the third thing?
99. I: Education.
100. R: Yes, I already said that the intensive education would help the people who come here to understand the information on circumcision. Things like the benefits of VMMC, where to get it done, the challenges that may be there and other things concerning VMMC as well as where to go when they experience the challenges.
101. I: Okay, for the clinic, what are your thoughts on the workload or the number of things that need to be done? Is it okay; what would happen?
102. R: What do you mean by workload?
103. I: The clients come in, they are educated, then the messages have to be sent and later on reimbursed, all those need someone to do them. In terms of handling those activities, how do you think that would work?
104. R: On that, I do not know… the staff who will be doing that are the same ones or there will be extra staff.
105. I: If it is the same staff, what would happen?
106. R: If is the same [chuckles] if it is the same, it is just a matter of delivering the information. When they have agreed, you get their phone number and send the messages when the times comes. I do not think it is a major problem.
107. I: It is not a major problem.
108. R: Yes [laughs] it is not a big problem.
109. I: Why do you say that?
110. R: The VMMC will be done here?
111. I: At the VMMC clinic.
112. R: What will be done here is the…
113. I: The three things I mentioned.
114. R: Okay, I do not think it is a big problem.
115. I: Why do you say that?
116. R: [Laughs] the things are not many. Teaching does not take long; it is for a short period. Even sending the SMS…maybe as the numbers of people increases. When the numbers start to increase, when they have understood and they are interested in the VMMC, I think that is where the challenge will be. If it is the same staff and the numbers are increasing, it means there will be need to add more people.
117. I: Okay, if you were to choose one strategy of the three, which you feel would be most effective in increasing VMMC uptake or two that you fell would work better; which one or which ones would you choose?
118. R: Mainly the education. A patient needs to know, to be taught and they should understand the information on VMMC. Combining that with [chuckles] maybe the SMSs.
119. I: Education and SMS reminders?
120. R: Yes. For the reimbursement, we already said it would be a challenge after the program ends and there is no more money for transport.
121. I: Okay, so for continuity, you are opting for education and SMSs?
122. R: Yes.
123. I: If the government were able to do the reimbursements, which combination would you opt for?
124. R: It means I would choose the reimbursement and education.
125. I: [Chuckles] okay, I understand.
126. R: [Laughs]
127. I: In implementing these strategies, how well do you thee strategies fit into what already happens in the STI clinic?
128. R: [Silence] I do not understand that one.
129. I: Implementing these strategies into what already happens in the STI clinic; do you think they will be so foreign or they will easily blend into what already happens or … do you understand it?
130. R: It would not be foreign, because the education already happens. Every morning, people learn about STIs. There is some education on research as well and people are used to that. The people that come here are frequenters. Therefore, they know that when we go there, this is what we will learn.
131. I: Okay, and how well do you think they would fit into our culture or religious beliefs?
132. R: In terms of our religion and culture… fitting into them like what?
133. I: Would they cause any chaos or would they easily fit in?
134. R: People will accept these without a problem. That is because previously, a long time ago, people believed in non-medical circumcision. With the way things have changed, people are opting for medical circumcision. Even the religious groups, the Muslims for instance, previously they were doing the non-medical circumcision. Currently, we see them taking their Muslim children and bringing them for medical circumcision. After the hospital, they perform their ceremonies according to their cultures. Currently, the religious groups understand it because of the benefits that medical circumcision has as I said at the beginning.
135. I: Okay, so it would not be a problem.
136. R: Yes.
137. I: Alright, is there anything you would like to share with me on circumcision and increasing VMMC uptake; anything you feel we did not discuss.
138. R: Mm, no. we have talked of a lot of things [chuckles]
139. I: There is nothing?
140. R: We have discussed and there is mothing to add or subtract.
141. I: Okay, is there any question?
142. R: Aaa, there is no question.
143. I: Alright, this is also the end of the questions that I had.
144. R: Alright.
145. I: Thank you very much for your time.
146. R: Thank you.

THE END
