# Supplementary material for: Acceptability, feasibility and appropriateness of intensified health education, SMS/phone tracing and transport reimbursement for uptake of voluntary medical male circumcision in a sexually transmitted infections clinic in Malawi: A mixed methods study
Source: PLoS One. 2025 Jan 24;20(1):e0301952. doi: 10.1371/journal.pone.0301952 (PMC11760565; doi:10.1371/journal.pone.0301952)
Supplement: S2 Data — (ZIP) [file pone.0301952.s005.zip › Survey data.docx]

**VMMC RITE: Acceptability, Feasibility, Appropriateness Results (Compressed)**

1. **Men**
2. Intensified health education
3. Acceptability – Baseline

| Acceptability | Completely disagree  1 | Disagree  2 | Neither agree nor disagree  3 | Agree  4 | Completely Agree  5 |
| --- | --- | --- | --- | --- | --- |
| I would like to learn about VMMC | 0(0) | 1(1.5) | 0(0) | 1(1.5) | 67(97) |
| I find learning about VMMC appealing | 1(1.4) | 0(0) | 0(0) | 2(2.9) | 66(95.7) |
| I approve learning about VMMC | 0(0) | 0(0) | 2(3.0) | 2(2.9) | 65(94.2) |
| Learning about VMMC at this clinic is a welcome idea to me | 0(0) | 2(2.9) | 0(0) | 2(2.9) | 65(94.2) |

1. Acceptability – Endline

| Acceptability | Completely disagree  1 | Disagree  2 | Neither agree nor disagree  3 | Agree  4 | Completely Agree  5 |
| --- | --- | --- | --- | --- | --- |
| I liked learning about VMMC | 0(0) | 0(0) | 0(0) | 1(5.3) | 18(94.7) |
| I found learning about VMMC appealing | 0(0) | 0(0) | 0(0) | 1(5.3) | 18(94.7) |
| I approve learning about VMMC | 0(0) | 0(0) | 0(0) | 2(10.5) | 17(89.5) |
| Learning about VMMC at this clinic was a welcome idea to me | 0(0) | 0(0) | 0(0) | 0(0) | 19(100) |

1. Appropriateness – Baseline

| Appropriateness | Completely disagree  1 | Disagree  2 | Neither agree nor disagree  3 | Agree  4 | Completely Agree  5 |
| --- | --- | --- | --- | --- | --- |
| 1. Openly learning about VMMC is a good match to this clinic | 1(1.5) | 0(0) | 0(0) | 1(1.5) | 67(97.1) |
| 1. Openly learning men about VMMC is applicable to this clinic | 3(4.4) | 0(0) | 15(21.7) | 7(10.1) | 44 (63.8) |
| 1. Openly learning about VMMC is a good fit to me/for this clinic (probe-culture) | 12(17.4) | 14(20.3) | 0(0) | 2(2.9) | 41(59.4) |
| 1. Openly learning about VMMC is suitable for me/this clinic (probe-religion) | 10(14.5) | 13(18.8) | 2(2.9) | 2(2.9) | 42(60.87) |

1. Appropriateness – Endline

| Appropriateness | Completely disagree  1 | Disagree  2 | Neither agree nor disagree  3 | Agree  4 | Completely Agree  5 |
| --- | --- | --- | --- | --- | --- |
| 1. Openly learning about VMMC is a good match to this clinic | 0(0) | 0(0) | 0(0) | 1(5.3) | 18(94.7) |
| 1. Openly learning about VMMC is applicable to this clinic | 0(0) | 0(0) | 6(31.6) | 2(10.5) | 11(57.9) |
| 1. Openly learning about VMMC is a good fit to me/for this clinic (probe-culture) | 3(15.8) | 8(42.1) | 0(0) | 2(10.5) | 6(31.6) |
| 1. Openly learning about VMMC is suitable for me/this clinic (probe-religion) | 1(5.3) | 10(52.6) | 0(0) | 2(10.5) | 6(31.6) |

1. Men
2. SMS
3. Acceptability – Baseline

| Acceptability | Completely disagree  1 | Disagree  2 | Neither agree nor disagree  3 | Agree  4 | Completely Agree  5 |
| --- | --- | --- | --- | --- | --- |
| 1. I would like to receive SMS reminders/calls about VMMC appointments | 0(0) | 0(0) | 0(0) | 4(9.3) | 39(90.7) |
| 1. I approve SMS reminders/calls for VMMC appointments at this clinic | 0(0) | 0(0) | 0(0) | 2(4.7) | 41(95.4) |
| 1. I welcome SMS reminders/calls for VMMC | 0(0) | 0(0) | 0(0) | 5(11.6) | 38(88.4) |
| 1. Receiving SMS reminders/calls about VMMC appointments is appealing to me | 1(2.3) | 2(4.7) | 0(0) | 19(44.2) | 21(48.9) |

1. Acceptability – Endline

| Acceptability | Completely disagree  1 | Disagree  2 | Neither agree nor disagree  3 | Agree  4 | Completely Agree  5 |
| --- | --- | --- | --- | --- | --- |
| 1. I liked to receive SMS reminders/calls about VMMC appointments | 0(0) | 1(2.3) | 0(0) | 27(62.8) | 15(34.9) |
| 1. I approve SMS reminders/calls for VMMC appointments at this clinic | 0(0) | 0(0) | 0(0) | 25(58.1) | 18(41.9) |
| 1. I welcomed SMS reminders/calls for VMMC | 0(0) | 1(2.3) | 0(0) | 31(72.1) | 11(25.6) |
| 1. Receiving SMS reminders/calls about VMMC appointments was appealing to me | 0(0) | 2(4.7) | 2(4.7) | 14(32.6) | 25(58.1) |

1. Appropriateness – Baseline

| Appropriateness | Completely disagree  1 | Disagree  2 | Neither agree nor disagree  3 | Agree  4 | Completely Agree  5 |
| --- | --- | --- | --- | --- | --- |
| 1. Receiving SMS reminders/calls about VMMC appointments is a good match for this clinic | 0(0) | 0(0) | 0(0) | 1(2.3) | 42(97.7) |
| 1. Receiving SMS reminders/calls about VMMC appointments is a good fit to me/for this clinic (probe-culture) | 9(20.9) | 22(51.2) | 0(0) | 5(11.6) | 7(16.3) |
| 1. Receiving SMS reminders/calls about VMMC appointments is suitable to me/this clinic (probe-religion) | 12(27.9) | 19(44.2) | 0(0) | 3(7) | 9(21) |
| 1. Receiving SMS reminders/calls about VMMC appointments is applicable to me/this clinic | 4(9.3) | 0(0) | 0(0) | 16(37.2) | 23(53.5) |

1. Appropriateness – Endline

| Appropriateness | Completely disagree  1 | Disagree  2 | Neither agree nor disagree  3 | Agree  4 | Completely Agree  5 |
| --- | --- | --- | --- | --- | --- |
| 1. Receiving SMS reminders/calls about VMMC appointments was a good match for this clinic | 0(0) | 0(0) | 0(0) | 32(74.4) | 11(25.6) |
| 1. Receiving SMS reminders/calls about VMMC appointments was a good fit to me/for this clinic (probe-culture) | 0(0) | 0(0) | 0(0) | 32(74.4) | 11(25.6) |
| 1. Receiving SMS reminders/calls about VMMC appointments was suitable to me/this clinic (probe-religion) | 0(0) | 2(4.7) | 1(2.3) | 23(53.5) | 17(39.5) |
| 1. Receiving SMS reminders/calls about VMMC appointments was applicable to me/this clinic | 0(0) | 0(0) | 0(0) | 15(34.9) | 28(65.1) |

1. Men
2. Transport reimbursement
3. Acceptability – Baseline

| Acceptability | Completely disagree  1 | Disagree  2 | Neither agree nor disagree  3 | Agree  4 | Completely Agree  5 |
| --- | --- | --- | --- | --- | --- |
| 1. I would like to receive transport reimbursement for my VMMC appointment. | 0(0) | 0(0) | 1(2.9) | 13(38.2) | 20(58.8) |
| 1. I approve transport reimbursements for VMMC at this clinic. | 0(0) | 0(0) | 0(0) | 12(35.3) | 22(64.7) |
| 1. Transport reimbursements are a welcome idea to me. | 0(0) | 0(0) | 0(0) | 10(29.4) | 24(70.6) |
| 1. Receiving transport reimbursement for my VMMC appointment is appealing. | 0(0) | 0(0) | 0(0) | 20(58.8) | 14(41.2) |

1. Acceptability – Endline

| Acceptability | Completely disagree  1 | Disagree  2 | Neither agree nor disagree  3 | Agree  4 | Completely Agree  5 |
| --- | --- | --- | --- | --- | --- |
| 1. I liked receiving transport reimbursement for my VMMC appointment. | 0(0) | 0(0) | 1(3.3) | 9(30) | 20(66.7) |
| 1. Transport reimbursements were a welcome idea to me. | 0(0) | 0(0) | 0(0) | 17(56.7) | 13(43.3) |
| 1. I approved transport reimbursements for VMMC at this clinic. | 0(0) | 0(0) | 0(0) | 11(36.7) | 19(63.3) |
| 1. Receiving transport reimbursement for my VMMC appointment was appealing. | 0(0) | 0(0) | 1(3.3) | 17(56.7) | 13(43.3 |

1. Appropriateness – Baseline

| Appropriateness | Completely disagree  1 | Disagree  2 | Neither agree nor disagree  3 | Agree  4 | Completely Agree  5 |
| --- | --- | --- | --- | --- | --- |
| 1. Receiving transport reimbursement for VMMC appointment is a good match for me/this clinic | 0(0) | 0(0) | 0(0) | 12(35.3) | 22(64.7) |
| 1. Receiving transport reimbursement for VMMC appointment is a good fit to me/this clinic (probe-culture) | 11(32.4) | 19(55.9) | 0(0) | 1(2.9) | 3(8.9) |
| 1. Receiving transport reimbursement for VMMC appointment is suitable to me/this clinic (probe-religion) | 8(23.5) | 22(64.7) | 0(0) | 0(0) | 4(11.8) |
| 1. Receiving transport reimbursement for VMMC appointment is applicable to me/this clinic | 1(2.9) | 0(0) | 0(0) | 15(44.1) | 18(52.9) |

1. Appropriateness – Endline

| Appropriateness | Completely disagree  1 | Disagree  2 | Neither agree nor disagree  3 | Agree  4 | Completely Agree  5 |
| --- | --- | --- | --- | --- | --- |
| 1. Receiving transport reimbursement for VMMC appointment was a good match for me/this clinic | 0(0) | 0(0) | 0(0) | 12(40) | 18(60) |
| 1. Receiving transport reimbursement for VMMC appointment was a good fit to me/this clinic (probe-culture) | 0(0) | 0(0) | 0(0) | 21(70) | 9(30) |
| 1. Receiving transport reimbursement for VMMC appointment was suitable to me/this clinic (probe-religion) | 1(3.3) | 0(0) | 0(0) | 15(50) | 14(46.7) |
| 1. Receiving transport reimbursement for VMMC appointment was applicable to me/this clinic | 0(0) | 0(0) | 0(0) | 15(50) | 15(50) |

1. **Healthcare workers**
2. Intensified health education
3. Acceptability – Baseline

| Acceptability | Completely disagree  1 | Disagree  2 | Neither agree nor disagree  3 | Agree  4 | Completely Agree  5 |
| --- | --- | --- | --- | --- | --- |
| 1. I would like to educate men about VMMC | 1(8.3) | 0(0) | 0(0) | 6(50) | 5(41.7) |
| 1. I find educating men about VMMC appealing | 0(0) | 0(0) | 0(0) | 5(41.7) | 7(58.3) |
| 1. I approve educating men about VMMC | 0(0) | 0(0) | 0(0) | 5(41.7) | 7(58.3) |
| 1. Educating men about VMMC at this clinic is a welcome idea to me | 0(0) | 0(0) | 0(0) | 5(41.7) | 7(58.3) |

1. Acceptability – Endline

| Acceptability | Completely disagree  1 | Disagree  2 | Neither agree nor disagree  3 | Agree  4 | Completely Agree  5 |
| --- | --- | --- | --- | --- | --- |
| 1. I liked to educating men about VMMC | 0(0) | 0(0) | 0(0) | 5(50) | 5(50) |
| 1. I found educating men about VMMC appealing | 0(0) | 0(0) | 0(0) | 5(50) | 5(50) |
| 1. I approved educating men about VMMC | 0(0) | 0(0) | 0(0) | 3(30) | 7(70) |
| 1. Educating men about VMMC at this clinic was a welcome idea to me | 0(0) | 0(0) | 0(0) | 0(0) | 10(100) |

1. Appropriateness – Baseline

| Appropriateness | Completely disagree  1 | Disagree  2 | Neither agree nor disagree  3 | Agree  4 | Completely Agree  5 |
| --- | --- | --- | --- | --- | --- |
| 1. Openly educating men about VMMC is a good match to this clinic | 0(0) | 0(0) | 0(0) | 4(33.3) | 8(66.7) |
| 1. Openly educating men about VMMC is a good fit to me/for this clinic (probe-culture) | 1(8.3) | 0(0) | 0(0) | 5(41.7) | 6(60) |
| 1. Openly educating men about VMMC is suitable for me/this clinic (probe-religion) | 1(8.3) | 0(0) | 0(0) | 5(41.7) | 6(60) |
| 1. Openly educating men about VMMC is applicable to this clinic | 0(0) | 1(8.3) | 3(25) | 3(25) | 5(41.7) |

1. Appropriateness – Endline

| Appropriateness | Completely disagree  1 | Disagree  2 | Neither agree nor disagree  3 | Agree  4 | Completely Agree  5 |
| --- | --- | --- | --- | --- | --- |
| 1. Openly educating men about VMMC was a good match to this clinic | 0(0) | 0(0) | 0(0) | 5(50) | 5(50) |
| 1. Openly educating men about VMMC was a good fit to me/for this clinic (probe-culture) | 1(10) | 0(0) | 0(0) | 6(60) | 3(30) |
| 1. Openly educating men about VMMC was suitable for me/this clinic (probe-religion) | 0(0) | 0(0) | 0(0) | 5(50) | 5(50) |
| 1. Openly educating men about VMMC was applicable to this clinic | 0(0) | 0(0) | 2(20) | 4(40) | 4(40) |

1. Feasibility

| Feasibility | Completely disagree  1 | Disagree  2 | Neither agree nor disagree  3 | Agree  4 | Completely Agree  5 |
| --- | --- | --- | --- | --- | --- |
| 1. Intensified health education was implementable at this clinic | 0(0) | 0(0) | 0(0) | 4(40) | 6(60) |
| 1. It was possible to routinely conduct intensified health education at this clinic. | 1(10) | 0(0) | 0(0) | 6(60) | 3(30) |
| 1. Intensified health education was easy to conduct. | 0(0) | 1(10) | 0(0) | 6(60) | 3(30) |
| 1. Intensified health education was doable at this clinic | 0(0) | 0(0) | 1(10) | 7(70) | 2(20) |

1. Healthcare workers
2. SMS
3. Acceptability – Baseline

| Acceptability | Completely disagree  1 | Disagree  2 | Neither agree nor disagree  3 | Agree  4 | Completely Agree  5 |
| --- | --- | --- | --- | --- | --- |
| 1. I would like to send patients SMS reminders/calling patients about their VMMC appointment | 0(0) | 0(0) | 0(0) | 4(57.1) | 3(42.9) |
| 1. Sending SMS/calling patients about their VMMC appointment is appealing to me | 0(0) | 0(0) | 1(14.3) | 1(14.3) | 5(71.4) |
| 1. I approve SMS reminders/calling patients for VMMC appointments | 0(0) | 0(0) | 1(14.3) | 4(57.1) | 2(28.6) |
| 1. Sending SMS reminders/calling patients for VMMC appointments is a welcome idea to me | 0(0) | 0(0) | 0(0) | 5(71.4) | 2(28.6) |

5(71.4)

1. Acceptability – Endline

| Acceptability | Completely disagree  1 | Disagree  2 | Neither agree nor disagree  3 | Agree  4 | Completely Agree  5 |
| --- | --- | --- | --- | --- | --- |
| 1. I liked to send patients SMS reminders/calling patients about their VMMC appointment | 0(0) | 0(0) | 0(0) | 2(100) | 0(0) |
| 1. Sending SMS/calling patients about their VMMC appointment was appealing to me | 0(0) | 0(0) | 0(0) | 1(50) | 1(50) |
| 1. I approved SMS reminders/calling patients for VMMC appointments | 0(0) | 0(0) | 0(0) | 2(100) | 0(0) |
| 1. Sending SMS reminders/calling patients for VMMC appointments was a welcome idea to me | 0(0) | 0(0) | 0(0) | 1(50) | 1(50) |

1. Appropriateness – Baseline

| Appropriateness | Completely disagree  1 | Disagree  2 | Neither agree nor disagree  3 | Agree  4 | Completely Agree  5 |
| --- | --- | --- | --- | --- | --- |
| 1. Sending SMS reminders/calling patients about VMMC appointments is a good match for this clinic | 0(0) | 0(0) | 0(0) | 5(71.4) | 2(28.6) |
| 1. Sending SMS reminders/calling patients about VMMC appointments is a good fit to me/for this clinic (probe-culture) | 0(0) | 1(14.3) | 2(28.6) | 3(42.8) | 1(14.3) |
| 1. Sending SMS reminders/calling patients about VMMC appointments is suitable to me/this clinic (probe-religion) | 0(0) | 1(14.3) | 2(28.6) | 3(42.8) | 1(14.3) |
| 1. Sending) SMS reminders/calls about VMMC appointments is applicable to me/this clinic | 1(14.3) | 0(0) | 0(0) | 2(28.6) | 4(57.1) |

1. Appropriateness – Endline

| Appropriateness | Completely disagree  1 | Disagree  2 | Neither agree nor disagree  3 | Agree  4 | Completely Agree  5 |
| --- | --- | --- | --- | --- | --- |
| 1. Sending SMS reminders/calling patients about VMMC appointments was a good match for this clinic | 0(0) | 0(0) | 0(0) | 7(100) | 0(0) |
| 1. Sending SMS reminders/calling patients about VMMC appointments was a good fit to me/for this clinic (probe-culture) | 0(0) | 0(0) | 0(0) | 7(100) | 0(0) |
| 1. Sending SMS reminders/calling patients about VMMC appointments was suitable to me/this clinic (probe-religion) | 0(0) | 0(0) | 0(0) | 7(100) | 0(0) |
| 1. Sending) SMS reminders/calls about VMMC appointments was applicable to me/this clinic | 0(0) | 0(0) | 0(0) | 7(100) | 0(0) |

1. Feasibility

| Feasibility | Completely disagree  1 | Disagree  2 | Neither agree nor disagree  3 | Agree  4 | Completely Agree  5 |
| --- | --- | --- | --- | --- | --- |
| 1. SMS/phone reminders were implementable at this clinic | 0(0) | 0(0) | 0(0) | 6(100) | 0(0) |
| 1. It was possible to routinely conduct SMS/phone reminders at this clinic | 0(0) | 0(0) | 0(0) | 6(100) | 0(0) |
| 1. SMS/phone reminders were easy to conduct | 0(0) | 0(0) | 0(0) | 3(50) | 3(50) |
| 1. SMS/phone reminders was doable at this clinic | 0(0) | 3(50) | 3(50) | 0(0) | 0(0) |

1. Healthcare workers
2. Transport Reimbursement
3. Acceptability – Baseline

| Acceptability | Completely disagree  1 | Disagree  2 | Neither agree nor disagree  3 | Agree  4 | Completely Agree  5 |
| --- | --- | --- | --- | --- | --- |
| 1. I like the idea of providing transport reimbursement for men who will undergo VMMC. | 0(0) | 0(0) | 0(0) | 5(71.4) | 2(28.6) |
| 1. Providing transport reimbursement for VMMC appointments is appealing | 0(0) | 1(14.3) | 0(0) | 5(71.4) | 1(14.3) |
| 1. I approve transport reimbursements for VMMC at this clinic. | 0(0) | 0(0) | 0(0) | 5(71.4) | 2(28.6) |
| 1. Transport reimbursements are a welcome idea to me. | 0(0) | 0(0) | 0(0) | 5(71.4) | 2(28.6) |

1. Acceptability - Endline

| Acceptability | Completely disagree  1 | Disagree  2 | Neither agree nor disagree  3 | Agree  4 | Completely Agree  5 |
| --- | --- | --- | --- | --- | --- |
| 1. I liked providing transport reimbursement for VMMC appointments | 0(0) | 0(0) | 0(0) | 5(100) | 0(0) |
| 1. Receiving transport reimbursement for my VMMC appointment was appealing | 0(0) | 0(0) | 0(0) | 1(20) | 4(80) |
| 1. I approve transport reimbursements for VMMC appointments | 0(0) | 0(0) | 0(0) | 5(100) | 0(0) |
| 1. Providing transport reimbursements for VMMC appointments is welcome to me | 0(0) | 0(0) | 0(0) | 5(100) | 0(0) |

1. Appropriateness – Baseline

| Appropriateness | Completely disagree  1 | Disagree  2 | Neither agree nor disagree  3 | Agree  4 | Completely Agree  5 |
| --- | --- | --- | --- | --- | --- |
| 1. Providing transport reimbursement for VMMC appointment is a good match for me/this clinic | 0(0) | 0(0) | 0(0) | 6(85.7) | 1(14.3) |
| 1. Providing transport reimbursement for VMMC appointment is a good fit to me/this clinic (probe-culture) | 0(0) | 1(14.3) | 0(0) | 5(71.4) | 1(14.3) |
| 1. Providing transport reimbursement for VMMC appointment is suitable to me/this clinic (probe-religion) | 0(0) | 1(14.3) | 0(0) | 5(71.4) | 1(14.3) |
| 1. Providing transport reimbursement for VMMC appointment is applicable to me/this clinic | 0(0) | 0(0) | 2(28.6) | 2(28.6) | 3(42.8) |

1. Appropriateness – Endline

| Appropriateness | Completely disagree  1 | Disagree  2 | Neither agree nor disagree  3 | Agree  4 | Completely Agree  5 |
| --- | --- | --- | --- | --- | --- |
| 1. Providing transport reimbursement for VMMC appointment was a good match for me/this clinic | 0(0) | 0(0) | 0(0) | 5(100) | 0(0) |
| 1. Providing transport reimbursement for VMMC appointment was a good fit to me/this clinic (probe-culture) | 0(0) | 0(0) | 0(0) | 5(100) | 0(0) |
| 1. Providing transport reimbursement for VMMC appointment was suitable to me/this clinic (probe-religion) | 0(0) | 0(0) | 0(0) | 5(100) | 0(0) |
| 1. Providing transport reimbursement for VMMC appointment was applicable to me/this clinic | 0(0) | 0(0) | 0(0) | 3(60) | 2(40) |

1. Feasibility

| Feasibility | Completely disagree  1 | Disagree  2 | Neither agree nor disagree  3 | Agree  4 | Completely Agree  5 |
| --- | --- | --- | --- | --- | --- |
| 1. Transport reimbursements were implementable at this clinic | 0(0) | 1(20) | 1(20) | 3(60) | 0(0) |
| 1. It was possible to routinely provide transport reimbursements at this clinic. | 1(20) | 0(0) | 1(20) | 3(60) | 0(0) |
| 1. Proving transport reimbursements was easy. | 0(0) | 2(40) | 0(0) | 3(60) | 0(0) |
| 1. Proving transport reimbursements was doable at this clinic. | 0(0) | 3(60) | 0(0) | 2(40) | 0(0) |
